# Supplementary material for: The Intra-European Union Food Trade with the Relation to the Notifications in the Rapid Alert System for Food and Feed
Source: Int J Environ Res Public Health. 2021 Feb 8;18(4):1623. doi: 10.3390/ijerph18041623 (PMC7915521; doi:10.3390/ijerph18041623)
Supplement: Supplementary file 1 [file ijerph-18-01623-s001.pdf]

Supplementary Material

The intra-European Union food flow with the relation to the food safety notifications in the Rapid Alert System for Food and Feed

Table S1. The intra-EU food trade in total trade in 1999-2018 for all EU countries according to the Eurostat (SITC).

Year	Export						Import					
	Billions of kg		Percentage in total	Billions of euros		Percentage in total	Billions of kg		Percentage in total	Billions of euros		Percentage in total
	Food	Total		Food	Total		Food	Total		Food	Total	
1999	195,5	1309,5	14,9%	143,0	1526,3	9,4%	182,8	1251,5	14,6%	140,5	1448,5	9,7%
2000	199,8	1400,4	14,3%	154,1	1805,3	8,5%	182,1	1348,1	13,5%	149,0	1717,8	8,7%
2001	215,7	1454,8	14,8%	164,5	1874,5	8,8%	197,6	1342,8	14,7%	160,3	1768,8	9,1%
2002	213,9	1456,3	14,7%	171,3	1904,8	9,0%	205,8	1396,3	14,7%	166,9	1793,8	9,3%
2003	230,7	1485,9	15,5%	176,7	1923,4	9,2%	213,9	1441,9	14,8%	172,5	1828,1	9,4%
2004	234,7	1579,3	14,9%	185,6	2081,2	8,9%	227,5	1553,7	14,6%	183,5	2000,8	9,2%
2005	248,2	1600,4	15,5%	198,6	2220,9	8,9%	244,2	1576,4	15,5%	196,1	2138,4	9,2%
2006	238,1	1571,8	15,1%	213,3	2502,3	8,5%	250,0	1570,2	15,9%	212,1	2393,2	8,9%
2007	250,4	1637,8	15,3%	236,3	2667,9	8,9%	251,7	1606,8	15,7%	234,0	2604,0	9,0%
2008	263,1	1584,6	16,6%	258,2	2726,5	9,5%	269,0	1580,8	17,0%	254,7	2656,1	9,6%
2009	275,3	1446,3	19,0%	242,1	2202,2	11,0%	275,8	1417,3	19,5%	241,6	2141,6	11,3%
2010	289,5	1641,0	17,6%	262,6	2548,2	10,3%	290,8	1645,8	17,7%	259,6	2481,4	10,5%
2011	286,9	1687,1	17,0%	288,7	2811,4	10,3%	289,6	1716,7	16,9%	285,8	2749,6	10,4%
2012	300,8	1694,1	17,8%	303,6	2824,5	10,7%	295,3	1706,8	17,3%	300,1	2762,8	10,9%
2013	307,9	1699,5	18,1%	317,5	2822,9	11,2%	298,6	1711,2	17,4%	312,1	2763,7	11,3%
2014	305,7	1768,4	17,3%	320,4	2919,6	11,0%	309,6	1957,9	15,8%	315,8	2853,2	11,1%
2015	320,8	1776,8	18,1%	334,4	3061,4	10,9%	325,6	1800,7	18,1%	331,7	2992,2	11,1%
2016	331,6	1818,9	18,2%	345,1	3108,4	11,1%	335,1	1850,7	18,1%	342,3	3042,3	11,2%
2017	332,3	1883,8	17,6%	365,7	3341,8	10,9%	338,9	1901,1	17,8%	364,1	3279,0	11,1%
2018	332,6	1892,2	17,6%	369,8	3517,5	10,5%	341,2	1924,7	17,7%	369,1	3451,7	10,7%
Mean	268,7	1619,5	16,6%	252,6	2519,6	10,0%	266,3	1615,1	16,5%	249,6	2443,3	10,2%
Increase	70,1%			158,6%			86,6%			162,8%		

Table S2. The intra-EU food trade in total trade in 1999-2018 for Austria according to the Eurostat (SITC).

Year	Export						Import					
	Billions of kg		Percentage in total	Billions of euros		Percentage in total	Billions of kg		Percentage in total	Billions of euros		Percentage in total
	Food	Total		Food	Total		Food	Total		Food	Total	
1999	3,7	31,5	11,8%	2,7	47,9	5,6%	3,8	42,1	9,1%	3,6	55,3	6,6%
2000	4,1	34,0	12,2%	3,0	55,5	5,5%	4,1	45,4	9,1%	3,8	63,1	6,1%
2001	4,2	36,2	11,7%	3,4	60,0	5,7%	4,5	47,9	9,4%	4,3	67,5	6,3%
2002	4,9	38,7	12,6%	3,7	63,3	5,8%	5,0	50,1	9,9%	4,5	67,3	6,7%
2003	5,0	40,2	12,5%	4,2	65,7	6,4%	5,1	53,3	9,6%	4,7	72,5	6,5%
2004	4,9	42,1	11,6%	4,5	71,2	6,4%	5,3	57,2	9,3%	5,2	80,4	6,4%
2005	5,6	43,0	12,9%	4,9	73,5	6,7%	5,9	60,7	9,6%	5,5	82,9	6,6%
2006	5,8	45,8	12,7%	5,4	79,7	6,7%	6,2	66,2	9,3%	5,9	87,7	6,7%
2007	6,2	50,7	12,2%	6,0	88,1	6,8%	6,3	68,6	9,1%	6,6	95,1	7,0%
2008	6,6	51,2	12,9%	6,7	90,6	7,4%	6,5	64,8	10,0%	7,4	98,6	7,5%
2009	7,0	44,2	15,8%	6,2	71,5	8,6%	6,6	59,4	11,1%	7,0	80,4	8,7%
2010	6,9	47,7	14,4%	6,6	83,1	7,9%	7,1	65,5	10,8%	7,6	93,5	8,1%
2011	7,0	48,9	14,2%	7,1	90,9	7,8%	7,3	68,2	10,8%	8,4	106,4	7,9%
2012	7,3	47,5	15,3%	7,4	90,8	8,1%	7,3	67,4	10,8%	8,9	106,3	8,4%
2013	7,1	47,0	15,2%	7,5	92,4	8,2%	7,7	66,4	11,6%	9,1	105,7	8,6%
2014	7,4	48,2	15,5%	7,7	93,7	8,3%	8,0	64,9	12,4%	9,3	105,2	8,8%
2015	7,8	49,1	15,9%	8,0	96,5	8,2%	8,8	65,8	13,4%	9,6	108,0	8,9%
2016	7,8	50,7	15,5%	8,1	97,0	8,4%	8,5	68,8	12,4%	9,9	111,2	8,9%
2017	8,0	51,6	15,6%	8,7	105,8	8,2%	8,9	71,3	12,5%	10,4	120,3	8,7%
2018	8,0	52,7	15,2%	9,0	111,6	8,0%	8,8	72,9	12,1%	10,6	127,3	8,3%
Mean	6,3	45,1	13,9%	6,0	81,4	7,4%	6,6	61,3	10,7%	7,1	91,7	7,7%
Increase	114,9%			234,9%			131,8%			190,5%		

Table S3. The intra-EU food trade in total trade in 1999-2018 for Belgium according to the Eurostat (SITC).

Year	Export						Import					
	Billions of kg		Percentage in total	Billions of euros		Percentage in total	Billions of kg		Percentage in total	Billions of euros		Percentage in total
	Food	Total		Food	Total		Food	Total		Food	Total	
1999	17,7	141,6	12,5%	15,1	132,4	11,4%	25,1	165,8	15,2%	11,7	111,8	10,5%
2000	17,8	157,2	11,3%	16,1	156,7	10,3%	25,8	177,5	14,5%	12,2	136,2	8,9%
2001	18,7	151,9	12,3%	17,3	165,8	10,4%	26,3	178,8	14,7%	13,2	143,6	9,2%
2002	19,3	161,0	12,0%	17,7	172,6	10,3%	27,1	180,3	15,0%	13,6	152,9	8,9%
2003	20,0	169,8	11,8%	18,0	174,6	10,3%	26,2	185,7	14,1%	13,7	152,9	9,0%
2004	20,1	181,3	11,1%	18,7	190,3	9,8%	26,9	196,3	13,7%	14,1	167,3	8,4%
2005	20,7	182,0	11,4%	19,5	206,5	9,5%	29,3	199,6	14,7%	14,8	184,7	8,0%
2006	20,2	148,3	13,6%	20,3	223,4	9,1%	26,9	168,8	16,0%	15,2	200,1	7,6%
2007	21,3	152,2	14,0%	22,1	239,5	9,2%	26,8	167,7	16,0%	17,0	211,9	8,0%
2008	22,1	154,4	14,3%	24,2	246,8	9,8%	27,9	171,5	16,3%	18,9	221,6	8,5%
2009	21,2	132,5	16,0%	22,9	201,3	11,4%	28,9	159,2	18,2%	17,4	179,0	9,7%
2010	20,8	141,9	14,7%	23,4	224,6	10,4%	30,5	166,9	18,3%	18,4	204,0	9,0%
2011	21,8	146,5	14,9%	25,8	246,0	10,5%	29,8	171,5	17,3%	21,1	227,0	9,3%
2012	25,5	146,5	17,4%	27,3	243,1	11,2%	29,8	174,2	17,1%	22,1	230,9	9,6%
2013	24,7	147,5	16,8%	28,0	247,6	11,3%	29,3	172,9	17,0%	23,0	225,9	10,2%
2014	25,8	193,4	13,3%	28,1	250,6	11,2%	33,2	396,3	8,4%	23,0	221,8	10,4%
2015	26,3	192,4	13,7%	29,5	256,5	11,5%	32,6	213,5	15,3%	23,1	212,6	10,9%
2016	28,0	195,9	14,3%	30,9	259,1	11,9%	35,7	219,0	16,3%	24,9	218,6	11,4%
2017	28,3	201,9	14,0%	32,4	275,3	11,8%	35,6	222,0	16,0%	26,2	233,7	11,2%
2018	29,1	201,3	14,5%	32,1	289,4	11,1%	36,6	227,1	16,1%	26,1	248,6	10,5%
Mean	22,5	165,0	13,6%	23,5	220,1	10,7%	29,5	195,7	15,1%	18,5	194,3	9,5%
Increase	64,8%			112,3%			45,7%			122,7%		

Table S4. The intra-EU food trade in total trade in 1999-2018 for Bulgaria according to the Eurostat (SITC).

Year	Export						Import					
	Billions of kg		Percentage in total	Billions of euros		Percentage in total	Billions of kg		Percentage in total	Billions of euros		Percentage in total
	Food	Total		Food	Total		Food	Total		Food	Total	
1999	0,5	4,5	10,4%	0,3	2,1	11,8%	0,3	2,2	12,2%	0,1	2,8	4,7%
2000	0,4	5,7	6,9%	0,2	3,0	7,2%	0,3	3,1	10,9%	0,2	3,8	5,0%
2001	0,5	7,4	7,1%	0,3	3,5	7,8%	0,4	2,9	12,7%	0,2	4,6	4,8%
2002	1,6	7,9	20,2%	0,4	3,8	10,8%	0,5	2,9	16,5%	0,3	4,9	5,3%
2003	0,8	6,4	12,8%	0,4	4,3	8,9%	0,5	3,5	13,2%	0,3	5,6	4,9%
2004	1,0	6,9	14,0%	0,4	5,0	8,5%	0,4	4,0	10,3%	0,3	6,7	4,7%
2005	1,2	6,9	17,7%	0,5	5,7	9,0%	0,5	5,0	9,1%	0,4	7,9	4,7%
2006	1,7	8,2	20,3%	0,6	7,3	7,9%	0,5	5,8	9,3%	0,5	9,5	4,9%
2007	1,0	7,5	13,2%	0,7	8,3	8,5%	1,1	7,4	14,3%	1,0	12,9	7,4%
2008	2,0	8,2	24,2%	1,1	9,2	12,0%	1,2	7,0	17,0%	1,3	14,3	9,2%
2009	4,0	8,6	47,2%	1,4	7,7	18,6%	1,2	5,6	20,9%	1,3	10,1	12,6%
2010	4,5	10,0	45,3%	1,9	9,5	19,4%	1,4	6,3	22,8%	1,5	11,3	13,0%
2011	5,2	11,7	45,0%	2,4	12,7	18,7%	1,5	6,8	22,7%	1,8	13,9	12,8%
2012	4,9	11,3	43,3%	2,3	12,2	19,0%	1,6	7,1	22,6%	1,9	14,9	12,9%
2013	6,4	12,9	50,0%	2,6	13,3	19,8%	1,5	7,2	21,3%	1,9	15,4	12,6%
2014	5,8	12,6	45,8%	2,4	13,7	17,2%	1,6	7,8	20,7%	1,9	16,1	12,1%
2015	5,2	12,8	40,5%	2,3	14,8	15,9%	1,9	8,3	23,3%	2,1	16,8	12,4%
2016	6,6	14,6	45,1%	2,7	15,8	17,4%	1,9	8,7	22,1%	2,2	17,3	12,7%
2017	6,9	15,0	46,3%	2,9	17,5	16,6%	2,2	9,5	22,7%	2,4	19,2	12,7%
2018	7,8	16,1	48,3%	3,2	19,0	16,6%	2,1	9,5	22,7%	2,5	20,3	12,3%
Mean	3,4	9,8	34,9%	1,4	9,4	15,4%	1,1	6,0	18,8%	1,2	11,4	10,5%
Increase	1562,5%			1152,4%			717,3%			1766,1%		

Table S5. The intra-EU food trade in total trade in 1999-2018 for Croatia according to the Eurostat (SITC).

Year	Export						Import					
	Billions of kg		Percentage in total	Billions of euros		Percentage in total	Billions of kg		Percentage in total	Billions of euros		Percentage in total
	Food	Total		Food	Total		Food	Total		Food	Total	
1999	0,0	0,0	-	0,0	0,0	-	0,0	0,0	-	0,0	0,0	-
2000	0,0	8,2	0,0%	0,0	3,3	0,0%	0,0	4,6	0,0%	0,0	6,1	0,0%
2001	1,6	10,2	15,3%	0,2	3,5	4,8%	1,0	5,8	17,6%	0,7	7,4	9,0%
2002	1,8	9,1	19,4%	0,2	3,4	6,6%	0,9	6,3	14,2%	0,7	8,1	8,8%
2003	2,6	9,6	26,9%	0,3	3,7	7,9%	1,6	8,1	19,3%	0,8	9,2	8,4%
2004	2,0	10,9	18,6%	0,2	4,2	5,0%	1,7	8,9	18,7%	0,8	9,5	8,5%
2005	1,8	10,2	17,4%	0,4	4,5	8,2%	1,7	8,7	19,9%	0,9	10,2	8,8%
2006	2,2	12,4	17,4%	0,5	5,3	9,1%	1,9	8,9	20,8%	1,0	11,5	8,9%
2007	2,4	13,1	18,1%	0,4	5,4	7,6%	1,5	8,2	18,0%	1,0	12,2	8,4%
2008	2,3	12,0	19,0%	0,4	5,8	6,7%	1,4	8,7	16,5%	1,1	13,3	8,5%
2009	2,5	11,5	21,9%	0,4	4,5	8,9%	1,1	6,5	17,2%	1,0	9,5	10,9%
2010	1,4	9,9	13,8%	0,4	5,4	7,7%	1,3	6,6	19,1%	1,1	9,1	11,8%
2011	1,7	9,2	18,3%	0,4	5,7	7,8%	1,3	7,4	17,3%	1,2	10,1	11,9%
2012	2,1	8,5	24,9%	0,5	5,6	9,0%	1,2	7,4	15,6%	1,3	10,1	12,7%
2013	2,0	9,7	20,2%	0,5	5,9	8,8%	1,4	7,5	18,8%	1,5	11,1	13,8%
2014	1,7	9,9	16,8%	0,6	6,6	9,6%	1,8	8,3	21,6%	1,8	13,1	14,0%
2015	2,1	10,5	20,4%	0,8	7,7	10,5%	1,8	9,0	20,1%	2,0	14,4	14,0%
2016	2,5	11,6	21,2%	1,1	8,2	12,9%	2,0	9,8	20,4%	2,1	15,2	14,1%
2017	2,7	11,6	23,0%	1,1	9,1	12,4%	2,2	10,6	20,8%	2,4	17,1	14,2%
2018	2,7	11,8	22,9%	1,3	10,0	12,7%	2,3	11,1	21,1%	2,6	18,6	13,9%
Mean	1,9	10,0	18,9%	0,5	5,4	9,0%	1,4	7,6	18,4%	1,2	10,8	11,2%
Increase	73,3%			644,0%			129,2%			289,0%		

Table S6. The intra-EU food trade in total trade in 1999-2018 for Cyprus according to the Eurostat (SITC).

Year	Export						Import					
	Billions of kg		Percentage in total	Billions of euros		Percentage in total	Billions of kg		Percentage in total	Billions of euros		Percentage in total
	Food	Total		Food	Total		Food	Total		Food	Total	
1999	0,2	0,5	36,6%	0,1	0,2	36,4%	0,5	1,2	37,1%	0,2	1,7	13,3%
2000	0,1	0,8	19,8%	0,1	0,3	31,8%	0,5	1,3	35,8%	0,2	2,0	12,5%
2001	0,2	0,7	23,3%	0,1	0,3	34,3%	0,3	1,4	21,5%	0,3	2,2	11,4%
2002	0,2	0,8	22,9%	0,1	0,3	34,7%	0,2	1,2	20,7%	0,3	2,2	11,9%
2003	0,2	0,9	20,3%	0,1	0,3	40,5%	0,3	1,2	21,3%	0,3	2,2	12,5%
2004	0,2	0,6	33,8%	0,1	0,5	26,1%	0,5	2,3	19,9%	0,4	3,0	13,2%
2005	0,2	0,7	25,1%	0,1	0,7	16,4%	0,6	2,7	21,9%	0,5	3,4	13,4%
2006	0,2	0,7	24,2%	0,1	0,6	20,8%	0,6	2,6	23,9%	0,5	3,7	13,8%
2007	0,2	0,6	29,0%	0,1	0,6	22,8%	0,6	2,8	22,5%	0,6	4,3	13,8%
2008	0,2	0,5	29,3%	0,1	0,6	22,7%	5,7	8,3	68,3%	0,7	4,9	13,6%
2009	0,1	0,4	32,4%	0,1	0,5	25,3%	3,8	6,7	57,0%	0,7	4,1	16,1%
2010	0,1	0,4	29,7%	0,1	0,6	22,4%	0,8	3,5	23,2%	0,7	4,5	16,0%
2011	0,2	0,5	33,9%	0,2	0,7	21,2%	0,8	3,3	24,8%	0,8	4,3	17,7%
2012	0,1	0,5	28,2%	0,1	0,6	21,4%	0,8	2,9	26,8%	0,8	3,9	20,0%
2013	0,2	0,4	35,4%	0,2	0,7	23,2%	0,8	2,6	29,5%	0,8	3,3	23,1%
2014	0,2	0,4	39,2%	0,2	1,1	14,7%	0,8	3,0	27,6%	0,8	3,9	21,0%
2015	0,1	0,4	32,2%	0,2	1,2	14,0%	0,7	3,1	22,3%	0,8	4,0	19,6%
2016	0,2	0,5	34,2%	0,2	1,2	17,6%	0,7	3,5	21,4%	0,8	4,7	17,7%
2017	0,2	0,5	33,5%	0,2	1,0	24,1%	0,8	3,9	20,3%	0,9	4,9	18,1%
2018	0,2	0,6	30,6%	0,3	1,1	25,5%	0,8	3,9	21,2%	0,9	5,3	17,7%
Mean	0,2	0,6	28,6%	0,1	0,6	22,0%	1,0	3,1	33,5%	0,6	3,6	16,3%
Increase	-13,2%			232,6%			80,0%			326,3%		

Table S7. The intra-EU food trade in total trade in 1999-2018 for Czech Republic according to the Eurostat (SITC).

Year	Export						Import					
	Billions of kg		Percentage in total	Billions of euros		Percentage in total	Billions of kg		Percentage in total	Billions of euros		Percentage in total
	Food	Total		Food	Total		Food	Total		Food	Total	
1999	2,8	37,9	7,5%	0,8	21,9	3,9%	2,1	20,4	10,1%	1,1	20,5	5,3%
2000	3,4	38,5	8,8%	1,1	27,2	3,9%	2,3	22,8	10,3%	1,2	26,1	4,8%
2001	2,9	38,4	7,5%	1,2	32,4	3,6%	2,4	24,5	9,9%	1,4	30,3	4,6%
2002	6,0	39,7	15,1%	1,2	35,1	3,4%	2,6	24,9	10,5%	1,6	31,2	5,1%
2003	7,0	42,0	16,8%	1,3	37,8	3,3%	2,9	26,7	11,0%	1,7	32,7	5,3%
2004	7,4	46,6	16,0%	1,7	48,6	3,4%	3,6	38,1	9,4%	2,4	45,2	5,3%
2005	8,8	46,0	19,2%	2,3	54,0	4,2%	4,0	34,1	11,8%	3,0	50,1	5,9%
2006	8,9	40,5	21,9%	2,5	65,1	3,8%	4,2	31,4	13,2%	3,5	59,8	5,9%
2007	8,9	41,9	21,3%	3,1	76,6	4,1%	4,3	34,0	12,5%	4,1	69,1	6,0%
2008	9,4	43,1	21,8%	3,9	85,0	4,5%	4,1	33,2	12,4%	4,6	73,9	6,3%
2009	11,6	50,3	23,0%	3,5	68,9	5,0%	5,0	37,3	13,4%	4,5	58,5	7,7%
2010	11,5	55,6	20,7%	3,7	84,5	4,4%	5,2	41,4	12,6%	4,9	71,2	6,9%
2011	11,2	56,9	19,8%	4,4	97,5	4,5%	5,3	44,3	12,0%	5,6	81,1	6,9%
2012	12,7	56,9	22,3%	5,3	99,3	5,3%	5,3	43,3	12,3%	6,1	82,4	7,4%
2013	12,7	57,8	22,0%	5,5	99,0	5,5%	5,5	46,2	12,0%	6,3	82,8	7,6%
2014	14,0	60,2	23,3%	5,8	108,3	5,4%	6,0	48,0	12,5%	6,4	89,3	7,2%
2015	14,7	63,8	23,0%	6,6	118,5	5,6%	6,5	52,0	12,5%	7,2	97,8	7,4%
2016	14,6	63,1	23,1%	6,6	122,9	5,4%	6,5	52,4	12,4%	7,4	101,1	7,3%
2017	14,2	62,9	22,6%	6,6	135,1	4,9%	6,5	50,8	12,9%	7,7	111,2	7,0%
2018	13,4	66,8	20,1%	6,5	144,4	4,5%	6,9	52,9	13,0%	7,9	118,2	6,7%
Mean	9,8	50,4	19,4%	3,7	78,1	4,7%	4,6	37,9	12,0%	4,4	66,6	6,7%
Increase	372,3%			664,4%			232,0%			629,3%		

Table S8. The intra-EU food trade in total trade in 1999-2018 for Denmark according to the Eurostat (SITC).

Year	Export						Import					
	Billions of kg		Percentage in total	Billions of euros		Percentage in total	Billions of kg		Percentage in total	Billions of euros		Percentage in total
	Food	Total		Food	Total		Food	Total		Food	Total	
1999	4,9	29,3	16,8%	6,9	33,5	20,5%	3,9	26,4	14,9%	3,2	31,6	10,2%
2000	4,9	36,3	13,5%	7,4	39,2	18,8%	4,1	25,5	15,9%	3,4	35,6	9,6%
2001	5,0	33,6	14,7%	8,2	40,2	20,3%	4,4	26,3	16,8%	3,7	36,8	10,1%
2002	5,0	34,0	14,8%	8,1	42,5	19,0%	4,6	27,5	16,9%	4,0	39,8	10,2%
2003	5,3	34,7	15,4%	7,8	41,4	18,8%	4,8	25,9	18,5%	4,1	37,3	10,9%
2004	4,3	35,6	12,2%	7,9	43,6	18,1%	4,0	24,1	16,8%	4,4	38,8	11,2%
2005	4,7	36,0	13,0%	8,4	48,1	17,4%	4,3	24,3	17,6%	4,7	43,2	10,9%
2006	5,4	36,2	15,0%	9,2	52,4	17,6%	4,5	26,1	17,2%	5,3	49,2	10,8%
2007	6,0	38,0	15,7%	9,2	52,6	17,4%	5,3	27,6	19,2%	5,7	52,1	10,8%
2008	6,0	36,7	16,4%	9,7	55,2	17,6%	6,4	31,5	20,3%	6,2	53,3	11,7%
2009	6,9	33,6	20,7%	9,2	45,4	20,2%	5,6	25,7	21,6%	5,7	41,7	13,7%
2010	7,2	33,2	21,6%	10,2	47,6	21,3%	5,4	26,2	20,4%	5,8	43,8	13,3%
2011	7,3	32,2	22,6%	10,6	52,3	20,3%	5,5	27,6	19,8%	6,4	48,4	13,3%
2012	6,8	29,5	22,9%	10,8	52,8	20,4%	5,5	26,9	20,5%	6,8	50,2	13,6%
2013	6,9	29,2	23,5%	11,0	52,7	21,0%	5,5	27,9	19,7%	7,1	51,5	13,8%
2014	7,2	29,0	24,8%	11,1	53,3	20,9%	5,4	27,6	19,5%	7,2	52,1	13,8%
2015	7,2	29,6	24,4%	10,9	52,5	20,8%	5,7	27,3	20,9%	7,4	53,6	13,8%
2016	6,9	28,1	24,7%	11,4	52,8	21,5%	5,5	26,4	20,8%	7,4	55,1	13,5%
2017	7,3	29,3	24,7%	12,0	55,8	21,4%	5,6	28,5	19,6%	7,8	57,3	13,6%
2018	6,7	27,3	24,7%	11,6	56,8	20,5%	6,3	30,8	20,4%	8,1	60,8	13,4%
Mean	6,1	32,6	18,7%	9,6	48,5	19,7%	5,1	27,0	18,9%	5,7	46,6	12,3%
Increase	37,2%			69,4%			59,5%			153,3%		

Table S9. The intra-EU food trade in total trade in 1999-2018 for Estonia according to the Eurostat (SITC).

Year	Export						Import					
	Billions of kg		Percentage in total	Billions of euros		Percentage in total	Billions of kg		Percentage in total	Billions of euros		Percentage in total
	Food	Total		Food	Total		Food	Total		Food	Total	
1999	0,1	6,7	2,1%	0,1	1,9	5,5%	0,4	2,1	20,7%	0,3	2,4	11,1%
2000	0,2	7,7	2,1%	0,1	3,0	4,7%	0,6	2,1	26,8%	0,3	3,3	9,5%
2001	0,2	7,0	2,8%	0,2	3,0	5,5%	0,6	2,3	24,0%	0,4	3,2	11,2%
2002	0,2	7,4	3,3%	0,2	3,0	6,7%	0,6	2,9	19,2%	0,4	3,5	10,9%
2003	0,2	7,9	3,1%	0,2	3,3	6,5%	0,6	2,9	20,5%	0,4	3,7	10,7%
2004	0,3	7,4	4,1%	0,3	3,8	7,1%	0,7	4,1	17,0%	0,5	4,9	10,1%
2005	0,5	7,4	6,3%	0,3	4,8	6,8%	0,6	4,5	13,9%	0,6	6,3	9,7%
2006	0,5	7,4	6,7%	0,4	5,0	7,2%	0,7	5,6	11,9%	0,7	8,0	8,7%
2007	0,5	7,6	6,1%	0,4	5,6	7,6%	0,7	6,3	11,7%	0,9	9,0	10,4%
2008	0,6	7,8	8,1%	0,5	5,9	8,9%	0,8	6,1	12,7%	1,0	8,7	11,5%
2009	0,6	6,4	9,5%	0,5	4,5	10,2%	0,7	5,4	13,6%	0,8	5,8	14,1%
2010	0,7	8,4	8,3%	0,5	5,9	9,0%	0,8	5,8	14,3%	0,9	7,4	12,3%
2011	0,8	9,3	8,2%	0,7	7,8	8,7%	1,0	6,0	16,8%	1,1	9,6	11,9%
2012	0,9	9,0	10,3%	0,8	8,2	10,0%	0,9	6,0	14,7%	1,3	11,0	11,4%
2013	1,0	9,4	10,1%	0,9	8,7	9,8%	1,0	6,3	15,2%	1,4	11,4	11,9%
2014	1,0	9,7	10,3%	0,9	8,7	10,0%	0,9	6,2	14,8%	1,3	11,3	11,8%
2015	1,0	9,5	10,4%	0,8	8,7	9,4%	0,9	5,7	14,9%	1,3	10,7	11,8%
2016	1,1	9,7	10,9%	0,8	8,8	9,0%	0,8	6,1	13,5%	1,3	11,0	11,7%
2017	1,1	10,9	10,5%	0,9	9,2	9,7%	1,0	6,8	14,1%	1,4	12,0	11,7%
2018	1,1	12,0	9,0%	0,9	9,8	9,5%	0,9	7,1	13,4%	1,4	12,4	11,2%
Mean	0,6	8,4	7,5%	0,5	6,0	8,7%	0,8	5,0	15,1%	0,9	7,8	11,3%
Increase	664,2%			764,5%			115,2%			428,4%		

Table S10. The intra-EU food trade in total trade in 1999-2018 for Finland according to the Eurostat (SITC).

Year	Export						Import					
	Billions of kg		Percentage in total	Billions of euros		Percentage in total	Billions of kg		Percentage in total	Billions of euros		Percentage in total
	Food	Total		Food	Total		Food	Total		Food	Total	
1999	0,3	27,4	1,2%	0,4	25,7	1,6%	1,5	21,6	7,1%	1,4	20,3	6,9%
2000	0,4	27,2	1,5%	0,5	31,3	1,5%	1,5	23,8	6,2%	1,5	24,1	6,2%
2001	0,5	26,6	1,9%	0,5	29,0	1,7%	1,4	23,6	6,1%	1,6	24,5	6,3%
2002	0,5	27,5	1,7%	0,5	29,2	1,7%	1,6	23,3	6,6%	1,7	25,3	6,7%
2003	0,5	28,1	1,9%	0,5	28,3	1,9%	1,5	24,6	6,3%	1,8	25,7	7,1%
2004	0,6	28,8	2,1%	0,5	28,7	1,8%	1,7	24,0	7,2%	1,9	27,9	6,9%
2005	0,6	26,7	2,2%	0,5	29,9	1,8%	1,8	23,8	7,5%	2,0	31,5	6,4%
2006	0,6	29,6	2,0%	0,6	35,2	1,8%	1,9	25,9	7,2%	2,2	35,4	6,2%
2007	0,8	30,2	2,7%	0,7	37,3	1,7%	1,9	27,2	6,8%	2,4	38,2	6,3%
2008	0,8	28,3	2,9%	0,7	36,7	1,9%	2,0	26,9	7,4%	2,8	38,7	7,1%
2009	0,7	23,3	3,1%	0,6	25,1	2,3%	2,1	18,6	11,2%	2,7	28,4	9,5%
2010	0,9	27,3	3,4%	0,7	28,5	2,3%	2,1	24,9	8,5%	2,9	33,3	8,6%
2011	1,1	28,3	3,9%	0,8	31,5	2,5%	2,2	25,2	8,6%	3,3	37,2	8,7%
2012	0,9	27,1	3,5%	0,8	30,3	2,5%	2,4	23,6	10,2%	3,6	37,4	9,5%
2013	0,8	28,2	3,0%	0,8	30,8	2,5%	2,5	23,6	10,7%	3,9	38,7	10,0%
2014	1,0	28,2	3,5%	0,8	31,9	2,5%	2,5	22,9	11,1%	3,9	39,4	9,8%
2015	1,1	27,8	4,0%	0,9	31,6	2,8%	2,5	22,0	11,3%	3,8	39,8	9,6%
2016	1,0	29,1	3,4%	0,9	30,5	2,8%	2,5	21,4	11,6%	3,9	40,2	9,7%
2017	1,0	30,0	3,2%	1,0	35,6	2,9%	2,6	22,0	12,0%	4,1	44,8	9,3%
2018	0,9	31,3	2,7%	1,0	37,6	2,7%	2,7	24,3	11,1%	4,2	46,7	9,0%
Mean	0,8	28,1	2,7%	0,7	31,2	2,2%	2,0	23,7	8,6%	2,8	33,9	8,2%
Increase	154,8%			150,2%			75,4%			198,2%		

Table S11. The intra-EU food trade in total trade in 1999-2018 for France according to the Eurostat (SITC).

Year	Export						Import					
	Billions of kg		Percentage in total	Billions of euros		Percentage in total	Billions of kg		Percentage in total	Billions of euros		Percentage in total
	Food	Total		Food	Total		Food	Total		Food	Total	
1999	47,5	143,9	33,0%	25,3	198,9	12,7%	18,1	146,9	12,3%	19,4	202,9	9,6%
2000	48,4	153,5	31,5%	25,9	229,4	11,3%	19,0	159,0	12,0%	20,5	246,5	8,3%
2001	45,9	147,3	31,2%	25,6	231,4	11,1%	19,1	154,2	12,4%	21,2	247,5	8,6%
2002	44,2	143,3	30,9%	26,7	227,8	11,7%	18,8	153,3	12,3%	21,6	238,4	9,0%
2003	47,0	150,5	31,2%	27,7	230,6	12,0%	20,9	161,1	13,0%	22,3	247,1	9,0%
2004	47,3	154,9	30,5%	28,0	238,9	11,7%	28,0	181,7	15,4%	23,0	263,0	8,7%
2005	49,2	162,0	30,4%	28,2	235,7	12,0%	28,2	201,1	14,0%	23,6	272,9	8,6%
2006	43,9	142,3	30,8%	29,8	258,5	11,5%	27,5	182,7	15,1%	25,1	298,3	8,4%
2007	41,2	132,1	31,2%	32,5	265,4	12,2%	23,8	148,1	16,1%	27,2	319,5	8,5%
2008	40,1	128,6	31,2%	34,0	265,5	12,8%	29,6	147,3	20,1%	29,5	331,6	8,9%
2009	40,7	113,2	36,0%	30,2	214,8	14,1%	25,1	126,1	19,9%	29,1	280,0	10,4%
2010	47,2	145,0	32,5%	31,8	240,2	13,2%	31,0	196,3	15,8%	30,6	315,6	9,7%
2011	47,8	145,9	32,8%	36,4	260,3	14,0%	32,4	200,5	16,2%	33,5	348,5	9,6%
2012	46,7	140,8	33,2%	36,8	260,2	14,1%	31,6	200,3	15,8%	34,3	352,0	9,7%
2013	47,2	140,6	33,6%	37,7	259,0	14,5%	33,3	201,1	16,6%	36,3	348,5	10,4%
2014	46,7	144,7	32,3%	36,4	262,8	13,9%	30,9	192,9	16,0%	36,4	347,7	10,5%
2015	46,3	144,1	32,1%	36,0	268,3	13,4%	32,2	195,7	16,4%	37,9	355,6	10,6%
2016	44,7	140,1	31,9%	36,0	269,0	13,4%	30,8	196,3	15,7%	39,8	358,8	11,1%
2017	45,4	146,1	31,1%	37,4	278,2	13,4%	27,0	195,8	13,8%	41,8	381,4	11,0%
2018	48,3	149,0	32,4%	37,9	290,2	13,1%	26,6	194,6	13,7%	42,1	392,4	10,7%
Mean	45,8	143,4	31,9%	32,0	249,3	12,8%	26,7	176,7	15,1%	29,8	307,4	9,7%
Increase	1,5%			49,8%			46,8%			117,0%		

Table S12. The intra-EU food trade in total trade in 1999-2018 for Germany according to the Eurostat (SITC).

Year	Export						Import					
	Billions of kg		Percentage in total	Billions of euros		Percentage in total	Billions of kg		Percentage in total	Billions of euros		Percentage in total
	Food	Total		Food	Total		Food	Total		Food	Total	
1999	35,7	210,7	17,0%	19,0	335,2	5,7%	32,7	273,8	11,9%	27,8	294,3	9,4%
2000	37,3	227,7	16,4%	21,4	388,0	5,5%	34,2	302,9	11,3%	29,1	340,8	8,5%
2001	41,7	233,6	17,8%	23,7	407,6	5,8%	34,7	291,5	11,9%	30,9	351,2	8,8%
2002	41,8	242,0	17,3%	24,4	414,5	5,9%	36,5	294,6	12,4%	31,7	341,2	9,3%
2003	43,6	256,1	17,0%	25,5	433,1	5,9%	38,0	309,8	12,3%	32,7	353,9	9,2%
2004	44,9	282,4	15,9%	27,3	474,4	5,7%	37,9	332,0	11,4%	33,2	378,4	8,8%
2005	48,7	277,3	17,6%	30,5	502,8	6,1%	40,8	318,2	12,8%	35,6	403,3	8,8%
2006	44,1	290,8	15,2%	33,0	562,4	5,9%	43,8	334,7	13,1%	39,3	460,9	8,5%
2007	43,1	309,8	13,9%	37,5	625,3	6,0%	46,8	345,3	13,6%	43,0	498,0	8,6%
2008	44,1	308,3	14,3%	41,3	624,6	6,6%	48,2	356,4	13,5%	46,0	513,5	8,9%
2009	45,4	264,0	17,2%	39,1	501,5	7,8%	50,6	308,4	16,4%	43,7	429,5	10,2%
2010	48,1	283,8	17,0%	41,2	572,9	7,2%	51,3	358,0	14,3%	46,4	503,6	9,2%
2011	44,8	294,4	15,2%	45,3	629,9	7,2%	52,4	373,1	14,1%	50,8	572,5	8,9%
2012	45,3	275,8	16,4%	47,5	619,5	7,7%	53,5	361,5	14,8%	53,6	571,2	9,4%
2013	48,1	277,8	17,3%	50,5	618,3	8,2%	54,0	382,6	14,1%	56,8	575,1	9,9%
2014	47,8	288,5	16,6%	51,2	648,3	7,9%	55,9	395,7	14,1%	57,4	594,8	9,7%
2015	48,8	304,5	16,0%	52,1	692,3	7,5%	58,1	406,9	14,3%	60,4	621,6	9,7%
2016	51,2	305,0	16,8%	53,5	705,4	7,6%	59,5	405,1	14,7%	63,6	632,3	10,1%
2017	49,4	314,4	15,7%	56,0	749,6	7,5%	61,2	409,0	15,0%	68,5	682,2	10,0%
2018	48,7	320,4	15,2%	55,3	778,3	7,1%	61,3	412,2	14,9%	68,8	722,5	9,5%
Mean	45,1	278,4	16,2%	38,8	564,2	6,9%	47,6	348,6	13,6%	46,0	492,0	9,3%
Increase	36,1%			190,5%			87,5%			147,6%		

Table S13. The intra-EU food trade in total trade in 1999-2018 for Greece according to the Eurostat (SITC).

Year	Export						Import					
	Billions of kg		Percentage in total	Billions of euros		Percentage in total	Billions of kg		Percentage in total	Billions of euros		Percentage in total
	Food	Total		Food	Total		Food	Total		Food	Total	
1999	2,4	10,7	22,4%	2,2	6,9	31,8%	3,3	13,4	24,3%	3,1	20,0	15,7%
2000	2,1	11,4	18,2%	2,0	7,9	25,4%	3,6	13,6	26,7%	3,4	23,5	14,4%
2001	3,3	11,7	28,3%	2,2	8,1	27,5%	3,8	15,4	24,4%	3,4	22,6	15,0%
2002	2,4	9,0	26,8%	2,1	7,8	26,8%	3,2	12,1	26,3%	3,7	23,8	15,6%
2003	2,3	8,8	26,2%	2,2	8,4	26,5%	3,5	13,6	25,6%	3,8	25,7	14,9%
2004	2,0	8,9	22,3%	2,1	8,9	23,0%	3,6	14,1	25,7%	4,0	28,8	14,0%
2005	2,9	10,4	27,7%	2,6	9,5	27,5%	3,8	14,4	26,1%	4,2	28,6	14,5%
2006	2,8	10,4	27,0%	2,8	11,4	24,5%	3,9	15,1	25,6%	4,4	31,7	13,9%
2007	2,6	12,6	20,5%	2,8	12,3	22,9%	4,2	15,6	27,0%	5,0	36,1	13,7%
2008	6,8	17,6	38,8%	3,1	12,9	24,0%	3,7	14,3	26,1%	5,3	36,7	14,4%
2009	6,8	14,9	45,7%	3,0	10,2	29,6%	4,1	12,8	31,6%	5,1	30,3	17,0%
2010	3,1	11,2	27,8%	3,2	11,6	27,4%	4,2	12,7	33,0%	5,0	27,4	18,3%
2011	3,0	10,7	28,1%	3,4	12,5	27,2%	4,0	12,4	32,2%	5,0	25,2	20,0%
2012	2,9	10,0	29,1%	3,4	12,0	28,3%	3,6	11,5	30,9%	4,8	22,8	21,1%
2013	2,9	10,2	28,0%	3,6	12,3	29,6%	3,7	11,8	31,4%	4,9	22,2	22,0%
2014	2,9	11,1	26,6%	3,5	12,6	27,9%	3,8	12,0	31,5%	4,9	23,3	21,2%
2015	2,9	12,7	22,7%	4,1	14,0	29,2%	3,7	12,0	30,5%	4,8	23,1	20,7%
2016	3,4	13,8	24,5%	4,4	14,3	30,7%	4,0	12,6	32,1%	5,0	24,2	20,8%
2017	3,0	13,6	21,8%	4,4	15,5	28,2%	4,3	13,3	32,5%	5,3	26,2	20,3%
2018	3,3	14,7	22,5%	4,6	17,7	26,2%	4,3	13,9	31,0%	5,3	28,4	18,7%
Mean	3,2	11,7	27,2%	3,1	11,3	27,2%	3,8	13,3	28,6%	4,5	26,5	17,1%
Increase	37,5%			110,6%			32,1%			69,1%		

Table S14. The intra-EU food trade in total trade in 1999-2018 for Hungary according to the Eurostat (SITC).

Year	Export						Import					
	Billions of kg		Percentage in total	Billions of euros		Percentage in total	Billions of kg		Percentage in total	Billions of euros		Percentage in total
	Food	Total		Food	Total		Food	Total		Food	Total	
1999	3,7	14,9	24,6%	1,5	20,0	7,7%	0,7	12,2	6,0%	0,5	18,9	2,4%
2000	3,2	15,5	20,4%	1,7	25,7	6,5%	1,0	13,4	7,6%	0,6	23,1	2,7%
2001	4,4	17,0	25,7%	2,0	28,7	7,0%	1,1	14,3	8,0%	0,7	24,8	3,0%
2002	4,5	17,5	25,9%	2,0	31,2	6,3%	1,2	15,4	7,6%	0,9	26,0	3,3%
2003	4,4	17,4	25,2%	2,1	32,4	6,3%	1,4	16,4	8,8%	1,0	27,3	3,5%
2004	4,8	18,6	25,8%	2,3	37,7	6,1%	2,0	17,7	11,6%	1,6	33,4	4,7%
2005	5,9	22,3	26,4%	2,4	41,6	5,8%	2,3	18,8	12,4%	2,0	37,5	5,3%
2006	6,2	24,7	25,2%	2,6	48,4	5,4%	2,5	19,9	12,6%	2,3	43,9	5,2%
2007	9,7	30,0	32,3%	3,9	56,0	7,0%	2,9	21,2	13,8%	2,7	48,7	5,5%
2008	9,4	29,5	32,0%	4,6	58,8	7,8%	3,3	22,0	15,2%	3,3	50,8	6,5%
2009	10,2	28,0	36,4%	4,0	47,7	8,4%	3,1	17,9	17,2%	2,9	38,4	7,6%
2010	11,1	28,7	38,6%	4,8	56,5	8,5%	3,5	20,7	16,9%	3,3	45,3	7,4%
2011	10,3	29,6	34,7%	5,9	62,5	9,5%	3,7	21,9	16,9%	4,0	51,3	7,7%
2012	11,6	31,1	37,4%	6,7	62,4	10,7%	3,7	22,6	16,4%	3,9	52,4	7,5%
2013	10,9	31,3	34,8%	6,5	63,0	10,3%	3,7	23,9	15,6%	3,9	54,1	7,3%
2014	11,4	32,4	35,1%	6,2	66,6	9,3%	4,1	27,7	14,8%	4,1	59,4	7,0%
2015	13,0	34,4	37,7%	6,4	72,2	8,8%	4,3	28,2	15,2%	4,3	63,5	6,8%
2016	12,0	33,8	35,5%	6,3	75,0	8,4%	4,3	28,9	14,9%	4,6	65,9	7,0%
2017	14,6	39,0	37,4%	7,1	81,8	8,6%	4,3	31,1	13,8%	5,0	72,4	6,8%
2018	11,9	39,4	30,2%	7,0	87,1	8,0%	4,3	35,1	12,4%	5,2	77,1	6,7%
Mean	8,7	26,8	32,3%	4,3	52,8	8,1%	2,9	21,5	13,4%	2,8	45,7	6,2%
Increase	225,9%			353,0%			491,7%			1045,5%		

Table S15. The intra-EU food trade in total trade in 1999-2018 for Ireland according to the Eurostat (SITC).

Year	Export						Import					
	Billions of kg		Percentage in total	Billions of euros		Percentage in total	Billions of kg		Percentage in total	Billions of euros		Percentage in total
	Food	Total		Food	Total		Food	Total		Food	Total	
1999	2,1	9,5	22,6%	4,7	44,3	10,5%	3,4	18,7	18,1%	2,8	26,3	10,5%
2000	1,8	7,6	23,3%	4,6	51,7	8,9%	2,3	16,0	14,7%	2,5	32,1	7,8%
2001	1,9	8,6	21,8%	4,8	57,4	8,3%	2,8	17,3	16,4%	3,1	35,5	8,7%
2002	2,3	9,7	23,3%	5,3	60,9	8,7%	3,4	19,2	17,9%	3,5	36,0	9,6%
2003	2,2	9,9	22,7%	5,4	51,2	10,6%	3,5	19,3	18,0%	3,5	29,1	11,9%
2004	2,3	10,8	21,8%	5,6	52,9	10,7%	3,2	20,9	15,1%	3,5	31,8	11,1%
2005	2,6	11,0	23,3%	5,9	56,2	10,5%	3,6	23,9	14,9%	4,0	35,7	11,2%
2006	2,5	11,4	21,8%	6,6	54,8	12,0%	4,0	25,2	15,9%	4,4	38,8	11,3%
2007	2,8	11,2	25,4%	6,9	56,3	12,2%	3,9	27,7	14,1%	4,9	41,7	11,8%
2008	2,7	11,8	22,6%	6,6	53,7	12,2%	4,1	27,3	15,2%	5,1	38,9	13,2%
2009	2,6	10,0	25,8%	5,9	50,8	11,7%	4,6	22,9	20,3%	4,8	28,7	16,6%
2010	2,7	11,0	24,6%	6,4	51,2	12,6%	4,7	23,3	20,3%	4,9	31,4	15,6%
2011	3,0	12,1	25,2%	7,0	53,2	13,1%	4,4	22,6	19,2%	5,3	34,8	15,3%
2012	2,9	12,6	22,7%	7,1	54,7	12,9%	5,2	21,6	24,0%	5,9	35,3	16,7%
2013	3,4	12,3	27,7%	7,4	50,3	14,8%	5,9	23,7	24,8%	6,2	37,2	16,5%
2014	3,5	12,7	27,2%	7,7	50,1	15,4%	5,7	23,0	24,9%	6,3	41,4	15,3%
2015	3,8	14,6	25,9%	8,2	58,9	13,9%	6,3	24,1	25,9%	6,9	45,9	15,0%
2016	3,8	15,6	24,4%	8,1	58,9	13,7%	6,3	23,8	26,5%	6,8	47,9	14,3%
2017	4,0	14,7	27,2%	9,0	60,6	14,8%	6,7	25,7	26,3%	7,3	53,9	13,6%
2018	4,2	14,8	28,2%	9,2	69,9	13,2%	7,9	28,0	28,2%	7,7	58,0	13,3%
Mean	2,9	11,6	24,6%	6,6	54,9	12,1%	4,6	22,7	20,3%	5,0	38,0	13,1%
Increase	94,5%			98,8%			133,1%			180,9%		

Table S16. The intra-EU food trade in total trade in 1999-2018 for Italy according to the Eurostat (SITC).

Year	Export						Import					
	Billions of kg		Percentage in total	Billions of euros		Percentage in total	Billions of kg		Percentage in total	Billions of euros		Percentage in total
	Food	Total		Food	Total		Food	Total		Food	Total	
1999	17,3	74,7	23,2%	10,7	142,6	7,5%	18,5	97,5	19,0%	16,3	136,7	11,9%
2000	17,0	78,2	21,8%	11,1	161,5	6,9%	17,8	105,3	16,9%	16,9	158,5	10,6%
2001	19,2	82,3	23,3%	12,0	168,2	7,2%	18,4	102,2	18,0%	17,5	164,2	10,7%
2002	16,0	77,5	20,7%	12,6	165,9	7,6%	17,5	103,1	17,0%	17,8	165,1	10,8%
2003	17,1	81,6	20,9%	12,6	167,1	7,5%	19,7	103,2	19,0%	18,9	167,6	11,3%
2004	17,2	83,6	20,5%	13,4	178,1	7,5%	19,4	107,8	18,0%	20,1	178,9	11,3%
2005	17,4	85,4	20,3%	14,2	185,9	7,6%	19,4	106,6	18,2%	20,9	185,2	11,3%
2006	10,7	72,8	14,7%	15,2	205,7	7,4%	18,8	85,2	22,1%	22,3	204,4	10,9%
2007	18,8	84,7	22,2%	16,6	224,8	7,4%	18,8	84,9	22,1%	23,3	216,9	10,8%
2008	17,9	78,1	22,9%	18,1	219,7	8,2%	18,3	80,4	22,7%	24,0	210,2	11,4%
2009	16,1	63,8	25,2%	17,1	170,0	10,0%	20,4	70,9	28,8%	22,9	172,2	13,3%
2010	20,4	87,6	23,3%	18,7	195,3	9,6%	24,8	104,1	23,8%	25,2	202,9	12,4%
2011	19,3	86,1	22,4%	19,8	212,2	9,4%	24,0	104,9	22,9%	27,0	216,2	12,5%
2012	20,8	85,2	24,4%	20,5	211,1	9,7%	23,6	97,8	24,1%	26,9	201,8	13,3%
2013	22,6	82,9	27,2%	21,3	209,2	10,2%	23,8	98,7	24,1%	27,5	199,0	13,8%
2014	14,6	75,7	19,3%	21,8	218,2	10,0%	25,5	103,7	24,6%	28,0	203,5	13,7%
2015	20,7	84,6	24,5%	23,3	225,6	10,3%	24,5	104,0	23,6%	28,1	217,0	13,0%
2016	22,3	92,1	24,3%	24,3	233,0	10,4%	25,1	105,0	23,9%	28,6	223,1	12,8%
2017	17,9	91,3	19,6%	26,0	249,8	10,4%	25,9	109,0	23,8%	30,4	241,3	12,6%
2018	16,1	89,3	18,0%	26,6	261,7	10,2%	27,0	105,7	25,5%	30,2	250,5	12,1%
Mean	18,0	81,9	21,9%	17,8	200,3	8,9%	21,6	99,0	21,8%	23,6	195,8	12,1%
Increase	-7,4%			147,7%			45,4%			85,9%		

Table S17. The intra-EU food trade in total trade in 1999-2018 for Latvia according to the Eurostat (SITC).

Year	Export						Import					
	Billions of kg		Percentage in total	Billions of euros		Percentage in total	Billions of kg		Percentage in total	Billions of euros		Percentage in total
	Food	Total		Food	Total		Food	Total		Food	Total	
1999	0,1	6,9	1,6%	0,1	1,3	4,7%	0,5	2,4	19,2%	0,3	2,1	13,5%
2000	0,1	8,3	1,3%	0,1	1,6	4,5%	0,5	2,3	21,3%	0,4	2,6	13,7%
2001	0,2	8,1	2,2%	0,1	1,8	5,9%	0,5	2,3	21,4%	0,4	3,0	13,7%
2002	0,3	9,0	2,8%	0,1	1,9	6,6%	0,6	2,9	19,7%	0,5	3,3	13,7%
2003	0,3	9,9	2,8%	0,1	2,0	6,5%	0,6	3,1	17,9%	0,5	3,5	12,9%
2004	0,3	10,3	2,7%	0,2	2,4	8,0%	0,6	4,0	15,2%	0,5	4,2	13,0%
2005	0,9	11,4	7,7%	0,3	3,2	10,9%	0,8	5,5	13,9%	0,7	5,3	13,0%
2006	0,7	6,6	10,7%	0,4	3,6	11,6%	0,8	4,9	15,4%	0,9	7,0	12,2%
2007	0,8	6,6	11,7%	0,6	4,4	12,9%	0,8	5,6	14,6%	1,1	8,7	12,2%
2008	1,3	11,3	11,3%	0,7	4,7	14,3%	1,3	7,4	18,2%	1,3	8,3	15,3%
2009	1,4	10,7	13,1%	0,6	3,7	16,0%	1,5	4,7	32,2%	1,1	5,3	20,9%
2010	1,7	14,4	12,0%	0,8	4,8	15,6%	1,6	5,1	31,1%	1,3	6,7	19,0%
2011	1,4	14,6	9,6%	0,9	6,2	14,2%	1,5	6,2	24,3%	1,5	9,1	16,5%
2012	2,1	15,1	14,3%	1,2	6,9	17,8%	1,8	7,6	23,7%	1,8	10,5	16,9%
2013	1,9	14,0	13,7%	1,2	7,2	17,3%	1,8	7,6	23,2%	1,9	10,8	17,9%
2014	1,8	14,3	12,5%	1,1	7,6	14,0%	1,8	7,9	23,0%	1,9	10,9	17,1%
2015	2,2	14,5	15,3%	1,2	7,7	15,1%	1,7	8,0	21,6%	1,8	10,5	16,7%
2016	2,5	15,4	16,4%	1,3	7,8	16,1%	2,2	7,7	27,9%	1,9	10,5	18,4%
2017	2,4	16,0	15,3%	1,4	8,3	17,0%	2,4	8,5	27,8%	2,3	11,8	19,6%
2018	2,4	17,5	13,9%	1,4	9,1	15,6%	2,3	9,1	25,6%	2,3	12,5	18,7%
Mean	1,2	11,7	10,6%	0,7	4,8	14,2%	1,3	5,6	22,5%	1,2	7,3	16,5%
Increase	2161,8%			2291,0%			401,9%			720,5%		

Table S18. The intra-EU food trade in total trade in 1999-2018 for Lithuania according to the Eurostat (SITC).

Year	Export						Import					
	Billions of kg		Percentage in total	Billions of euros		Percentage in total	Billions of kg		Percentage in total	Billions of euros		Percentage in total
	Food	Total		Food	Total		Food	Total		Food	Total	
1999	0,2	6,6	3,5%	0,2	1,9	8,4%	0,4	1,9	21,6%	0,3	2,6	12,0%
2000	0,3	7,6	3,8%	0,3	2,9	8,8%	0,5	2,3	20,7%	0,3	3,1	10,8%
2001	0,4	9,8	4,2%	0,3	3,5	9,1%	0,5	2,7	19,1%	0,4	3,7	10,2%
2002	0,6	10,3	5,5%	0,4	3,8	9,3%	0,6	3,1	19,5%	0,4	4,5	9,2%
2003	0,6	8,7	6,4%	0,4	3,9	10,5%	0,6	3,2	19,5%	0,4	4,8	9,0%
2004	0,8	11,3	7,2%	0,6	5,0	12,2%	0,8	4,1	19,8%	0,6	6,3	10,0%
2005	1,7	13,2	13,0%	0,9	6,2	14,2%	1,0	4,8	19,9%	0,8	7,4	10,6%
2006	1,3	12,9	9,8%	1,0	7,2	14,0%	1,5	6,7	23,1%	1,1	9,7	11,0%
2007	1,5	13,2	11,5%	1,4	8,1	17,3%	1,8	7,9	22,2%	1,4	12,2	11,3%
2008	1,9	14,8	12,8%	1,4	9,7	14,9%	2,0	7,6	26,9%	1,9	12,2	15,3%
2009	2,4	13,9	17,6%	1,4	7,6	19,0%	1,7	5,5	30,2%	1,5	7,8	19,7%
2010	2,3	14,9	15,3%	1,6	9,6	17,2%	1,9	7,0	27,6%	1,9	10,0	19,0%
2011	2,2	16,9	13,0%	1,9	12,4	15,2%	2,2	8,1	26,8%	2,2	13,0	17,2%
2012	2,9	18,0	15,9%	2,2	13,9	15,8%	2,4	8,8	26,9%	2,6	14,3	18,4%
2013	2,6	17,5	15,1%	2,3	13,6	17,1%	2,5	9,1	27,9%	3,0	15,8	19,1%
2014	2,8	17,2	16,2%	2,4	13,3	18,1%	2,8	10,0	27,8%	3,0	17,0	17,4%
2015	3,5	18,7	18,8%	2,7	14,0	19,5%	3,0	9,6	31,8%	2,8	17,2	16,2%
2016	3,8	17,9	21,5%	2,8	13,7	20,7%	2,5	9,7	26,0%	2,6	17,6	14,9%
2017	3,9	19,1	20,4%	3,1	15,4	20,2%	2,7	10,9	24,8%	3,0	20,1	14,7%
2018	3,5	19,0	18,5%	3,1	16,6	18,6%	2,6	11,5	22,8%	3,0	21,3	14,2%
Mean	2,0	14,1	13,9%	1,5	9,1	16,7%	1,7	6,7	25,3%	1,7	11,0	15,1%
Increase	1445,8%			1830,4%			534,7%			863,1%		

Table S19. The intra-EU food trade in total trade in 1999-2018 for Luxembourg according to the Eurostat (SITC).

Year	Export						Import					
	Billions of kg		Percentage in total	Billions of euros		Percentage in total	Billions of kg		Percentage in total	Billions of euros		Percentage in total
	Food	Total		Food	Total		Food	Total		Food	Total	
1999	0,6	8,4	6,9%	0,5	6,8	7,3%	0,6	16,9	3,8%	1,0	8,7	12,1%
2000	0,5	14,4	3,8%	0,6	7,9	7,3%	0,7	18,4	3,7%	1,2	10,2	11,3%
2001	0,6	14,6	4,2%	0,6	9,6	6,4%	0,7	18,0	4,0%	1,2	11,2	10,6%
2002	0,6	12,9	5,0%	0,6	9,4	6,9%	0,7	16,8	3,9%	1,2	11,1	11,1%
2003	0,7	8,5	8,7%	0,7	10,3	6,5%	0,8	18,6	4,1%	1,3	11,1	12,1%
2004	0,6	10,5	6,1%	0,6	11,6	5,4%	0,7	20,9	3,5%	1,4	12,2	11,4%
2005	0,4	6,8	6,0%	0,7	13,4	5,3%	0,6	15,7	3,6%	1,4	13,2	10,9%
2006	0,5	7,8	5,8%	0,7	16,1	4,4%	0,6	17,4	3,4%	1,5	15,2	9,7%
2007	0,4	8,1	5,4%	0,7	14,4	5,0%	0,6	17,1	3,6%	1,6	15,1	10,3%
2008	0,5	7,8	5,8%	0,8	15,4	5,1%	0,6	16,2	3,8%	1,7	16,3	10,1%
2009	0,5	6,1	8,4%	0,8	12,9	6,0%	0,6	13,9	4,6%	1,6	13,0	12,5%
2010	0,7	7,6	8,7%	0,9	11,6	7,4%	0,9	18,3	5,2%	1,7	15,0	11,4%
2011	0,7	7,7	9,0%	0,9	11,9	7,8%	1,0	19,6	5,0%	1,8	16,9	10,6%
2012	0,7	6,8	10,2%	0,9	11,4	8,2%	1,0	17,9	5,8%	1,9	16,3	11,5%
2013	0,7	6,6	10,3%	0,9	11,1	8,5%	1,1	17,3	6,3%	2,0	15,9	12,3%
2014	0,7	6,9	10,9%	1,1	11,8	9,2%	1,3	17,8	7,4%	2,1	16,1	12,8%
2015	0,8	6,8	12,2%	1,1	12,8	8,8%	1,4	19,5	7,1%	2,1	15,2	13,5%
2016	0,8	7,1	11,8%	1,0	11,7	9,0%	1,4	19,0	7,1%	2,1	15,3	13,9%
2017	0,8	7,3	11,4%	1,1	11,7	9,5%	1,4	19,6	7,0%	2,2	16,9	13,1%
2018	0,9	7,4	12,2%	1,2	11,6	10,1%	1,4	20,2	7,0%	2,3	17,8	13,1%
Mean	0,6	8,5	7,6%	0,8	11,7	7,1%	0,9	18,0	5,0%	1,7	14,1	11,8%
Increase	54,5%			138,4%			118,9%			122,6%		

Table S20. The intra-EU food trade in total trade in 1999-2018 for Malta according to the Eurostat (SITC).

Year	Export						Import					
	Billions of kg		Percentage in total	Billions of euros		Percentage in total	Billions of kg		Percentage in total	Billions of euros		Percentage in total
	Food	Total		Food	Total		Food	Total		Food	Total	
1999	0,01	0,03	28,2%	0,01	0,91	1,3%	0,2	1,2	18,7%	0,2	1,8	12,3%
2000	0,01	0,04	23,0%	0,01	0,90	1,4%	0,2	1,5	15,8%	0,2	2,2	10,7%
2001	0,01	0,03	29,5%	0,01	1,07	1,2%	0,2	1,0	19,8%	0,2	1,8	13,2%
2002	0,01	0,04	25,3%	0,01	1,02	1,3%	0,2	1,1	17,2%	0,2	1,9	13,0%
2003	0,01	0,06	12,4%	0,01	0,97	1,4%	0,2	1,2	16,3%	0,2	1,9	12,4%
2004	0,01	0,06	16,1%	0,02	0,99	1,6%	0,4	1,6	25,9%	0,3	2,1	13,7%
2005	0,02	0,07	27,7%	0,02	0,99	2,1%	0,4	1,7	21,5%	0,3	2,3	13,9%
2006	0,01	0,07	18,1%	0,02	1,14	1,6%	0,3	1,9	17,7%	0,3	2,4	13,7%
2007	0,02	0,11	22,7%	0,02	1,09	1,8%	0,4	2,1	16,5%	0,4	2,6	15,4%
2008	0,06	0,12	52,3%	0,03	0,93	3,2%	0,3	2,2	15,2%	0,4	2,7	15,3%
2009	0,01	0,25	4,2%	0,02	0,80	2,1%	0,4	2,5	14,7%	0,4	2,4	17,0%
2010	0,01	0,38	3,1%	0,02	1,07	1,6%	0,4	2,5	14,4%	0,4	2,7	15,2%
2011	0,02	0,60	2,8%	0,03	1,24	2,1%	0,4	3,2	11,7%	0,4	3,3	13,1%
2012	0,03	0,50	5,8%	0,03	1,24	2,1%	0,4	3,9	9,1%	0,5	4,0	12,3%
2013	0,01	0,55	2,7%	0,02	1,18	1,8%	0,4	2,9	14,2%	0,5	3,3	15,7%
2014	0,02	0,38	5,1%	0,03	1,03	2,7%	0,4	3,2	12,5%	0,5	3,2	16,1%
2015	0,03	0,45	6,0%	0,04	1,02	3,4%	0,4	4,3	10,4%	0,5	3,6	15,1%
2016	0,03	0,72	4,3%	0,03	1,16	2,6%	0,4	4,3	9,7%	0,6	3,2	17,6%
2017	0,03	0,84	3,9%	0,03	1,35	2,1%	0,5	4,0	11,4%	0,6	3,3	18,2%
2018	0,01	0,61	1,3%	0,02	1,37	1,5%	0,5	4,1	12,6%	0,6	3,9	15,2%
Mean	0,02	0,30	6,3%	0,02	1,07	1,9%	0,3	2,5	13,8%	0,4	2,7	14,6%
Increase	-15,9%			63,3%			127,5%			170,1%		

Table S21. The intra-EU food trade in total trade in 1999-2018 for the Netherlands according to the Eurostat (SITC).

Year	Export						Import					
	Billions of kg		Percentage in total	Billions of euros		Percentage in total	Billions of kg		Percentage in total	Billions of euros		Percentage in total
	Food	Total		Food	Total		Food	Total		Food	Total	
1999	25,2	213,7	11,8%	27,0	168,3	16,0%	27,4	137,1	20,0%	12,7	110,1	11,5%
2000	24,8	208,5	11,9%	29,0	205,4	14,1%	20,6	135,9	15,2%	13,4	125,3	10,7%
2001	32,4	254,5	12,7%	30,9	210,2	14,7%	27,1	124,8	21,7%	14,7	121,7	12,1%
2002	27,4	237,6	11,6%	32,0	207,7	15,4%	28,3	152,8	18,5%	15,1	127,4	11,9%
2003	31,8	235,1	13,5%	32,2	210,6	15,3%	26,5	150,8	17,6%	15,7	128,1	12,2%
2004	35,1	260,4	13,5%	34,1	229,7	14,8%	27,9	159,7	17,5%	16,6	136,2	12,2%
2005	33,3	272,2	12,2%	35,6	260,8	13,6%	29,3	164,3	17,8%	16,4	144,4	11,4%
2006	34,9	284,7	12,3%	37,5	291,5	12,8%	33,2	194,0	17,1%	17,6	161,4	10,9%
2007	36,0	302,1	11,9%	41,4	313,7	13,2%	34,6	212,0	16,3%	19,5	176,4	11,1%
2008	35,7	238,4	15,0%	46,3	342,5	13,5%	31,8	155,0	20,5%	22,2	189,8	11,7%
2009	38,1	262,7	14,5%	43,0	276,4	15,5%	34,6	179,0	19,3%	20,5	155,6	13,1%
2010	39,3	309,8	12,7%	46,5	334,5	13,9%	35,9	187,8	19,1%	22,2	181,3	12,2%
2011	38,8	326,2	11,9%	50,0	369,1	13,5%	36,9	215,9	17,1%	25,7	199,0	12,9%
2012	40,4	366,8	11,0%	51,9	385,9	13,4%	38,2	231,9	16,5%	27,4	207,3	13,2%
2013	40,4	352,8	11,5%	54,9	380,2	14,4%	36,1	205,8	17,5%	28,1	205,2	13,7%
2014	38,8	342,6	11,3%	54,2	382,0	14,2%	36,7	195,6	18,8%	27,2	203,0	13,4%
2015	38,5	308,0	12,5%	55,1	388,0	14,2%	38,7	185,1	20,9%	29,5	211,2	13,9%
2016	43,5	334,0	13,0%	58,4	391,4	14,9%	41,1	207,1	19,9%	29,3	211,9	13,8%
2017	44,7	347,5	12,9%	62,1	432,9	14,3%	42,7	219,8	19,4%	31,7	233,3	13,6%
2018	44,4	326,7	13,6%	61,1	455,3	13,4%	42,9	214,4	20,0%	32,2	248,8	12,9%
Mean	36,2	289,2	12,5%	44,2	311,8	14,2%	33,5	181,4	18,5%	21,9	173,9	12,6%
Increase	75,8%			126,7%			56,5%			153,5%		

Table S22. The intra-EU food trade in total trade in 1999-2018 for Poland according to the Eurostat (SITC).

Year	Export						Import					
	Billions of kg		Percentage in total	Billions of euros		Percentage in total	Billions of kg		Percentage in total	Billions of euros		Percentage in total
	Food	Total		Food	Total		Food	Total		Food	Total	
1999	2,3	49,7	4,6%	1,5	21,0	7,2%	3,8	24,9	15,2%	1,7	31,1	5,4%
2000	2,3	54,8	4,1%	1,8	28,0	6,4%	5,1	27,3	18,7%	2,0	36,6	5,6%
2001	2,6	54,3	4,7%	2,1	32,7	6,5%	4,5	27,3	16,3%	2,2	39,1	5,7%
2002	2,7	54,9	4,9%	2,2	35,4	6,2%	7,7	30,8	25,1%	2,2	40,8	5,5%
2003	3,0	57,0	5,3%	2,6	39,1	6,6%	7,0	31,1	22,7%	2,1	42,0	4,9%
2004	4,0	60,9	6,6%	3,8	48,7	7,8%	8,6	38,5	22,3%	3,0	54,4	5,5%
2005	6,0	61,7	9,7%	5,3	56,7	9,3%	9,4	42,2	22,3%	4,0	61,5	6,5%
2006	6,4	63,0	10,1%	6,6	69,9	9,4%	10,6	46,9	22,7%	4,6	73,9	6,3%
2007	7,0	62,9	11,2%	8,0	81,0	9,9%	12,6	58,6	21,6%	5,9	88,7	6,6%
2008	7,5	60,0	12,5%	9,2	90,5	10,2%	13,4	64,2	20,9%	7,8	102,1	7,6%
2009	9,4	55,1	17,0%	9,0	78,2	11,6%	12,1	54,0	22,5%	7,0	77,9	9,1%
2010	9,5	66,1	14,4%	10,5	95,5	11,0%	13,8	60,2	22,9%	8,3	95,1	8,8%
2011	8,8	66,0	13,3%	11,5	105,9	10,9%	14,3	66,3	21,5%	9,5	105,9	9,0%
2012	11,3	69,2	16,4%	13,6	109,8	12,4%	14,8	61,3	24,1%	10,3	104,9	9,8%
2013	13,7	78,5	17,4%	15,6	115,6	13,5%	14,5	59,7	24,2%	11,0	107,8	10,2%
2014	14,8	83,3	17,8%	17,0	128,2	13,3%	15,3	64,4	23,7%	11,6	117,3	9,9%
2015	16,3	88,1	18,5%	19,2	142,4	13,5%	19,1	67,0	28,4%	12,2	125,3	9,7%
2016	15,6	88,3	17,7%	19,3	146,8	13,1%	19,6	70,4	27,8%	13,2	130,5	10,1%
2017	16,4	91,0	18,1%	22,3	165,8	13,4%	20,3	74,8	27,1%	14,8	148,3	10,0%
2018	16,8	95,5	17,6%	23,8	179,8	13,3%	20,6	78,1	26,4%	15,3	159,4	9,6%
Mean	8,8	68,0	13,0%	10,2	88,6	11,6%	12,4	52,4	23,6%	7,4	87,1	8,5%
Increase	631,3%			1481,5%			442,5%			802,0%		

Table S23. The intra-EU food trade in total trade in 1999-2018 for Portugal according to the Eurostat (SITC).

Year	Export						Import					
	Billions of kg		Percentage in total	Billions of euros		Percentage in total	Billions of kg		Percentage in total	Billions of euros		Percentage in total
	Food	Total		Food	Total		Food	Total		Food	Total	
1999	1,0	11,7	8,8%	1,2	19,4	6,2%	5,1	25,0	20,5%	3,2	29,6	10,9%
2000	1,2	12,4	9,5%	1,3	21,5	6,3%	4,9	25,5	19,1%	3,4	33,1	10,3%
2001	1,3	12,4	10,4%	1,4	21,9	6,5%	5,4	26,8	20,2%	3,8	33,8	11,2%
2002	1,6	14,1	11,2%	1,6	22,3	7,0%	5,1	28,1	18,3%	3,8	33,9	11,3%
2003	1,5	15,2	9,6%	1,6	22,8	7,0%	5,1	25,1	20,4%	3,9	33,2	11,9%
2004	1,5	15,3	9,8%	1,7	23,0	7,2%	5,0	23,7	21,2%	4,0	34,1	11,7%
2005	1,9	20,0	9,5%	2,0	25,0	8,1%	6,4	28,1	22,9%	4,6	39,9	11,4%
2006	2,0	22,2	8,9%	2,2	27,8	8,0%	6,2	26,8	23,1%	5,1	43,4	11,7%
2007	2,2	23,7	9,4%	2,6	29,5	8,9%	6,0	26,6	22,6%	5,5	45,9	12,0%
2008	2,8	22,8	12,2%	3,0	28,9	10,4%	6,4	27,2	23,5%	5,9	48,0	12,3%
2009	2,6	17,6	14,9%	2,9	23,7	12,1%	8,3	27,5	30,2%	5,9	40,4	14,7%
2010	2,7	19,3	13,7%	3,0	27,8	10,9%	8,1	29,4	27,7%	6,3	44,8	14,1%
2011	2,8	19,9	13,9%	3,3	31,4	10,4%	7,1	27,5	25,8%	6,6	43,7	15,0%
2012	2,8	19,7	14,3%	3,4	31,6	10,9%	7,3	26,3	27,7%	6,5	40,3	16,2%
2013	2,9	22,2	12,9%	3,6	32,8	11,1%	6,9	27,1	25,6%	6,8	41,1	16,6%
2014	3,1	22,5	13,9%	3,9	33,6	11,7%	7,4	29,9	24,8%	6,9	44,1	15,6%
2015	3,0	23,2	13,1%	4,3	35,7	12,0%	7,5	29,6	25,3%	7,1	46,2	15,4%
2016	3,0	22,9	13,3%	4,4	37,3	11,8%	7,9	30,5	25,9%	7,4	47,8	15,5%
2017	3,3	24,5	13,6%	4,7	40,3	11,6%	8,2	31,7	25,9%	8,1	53,2	15,3%
2018	3,6	25,8	14,1%	5,0	43,4	11,5%	8,2	31,5	26,1%	8,4	57,1	14,6%
Mean	2,3	19,4	12,1%	2,9	29,0	9,9%	6,6	27,7	24,0%	5,7	41,7	13,6%
Increase	252,3%			316,7%			60,0%			158,6%		

Table S24. The intra-EU food trade in total trade in 1999-2018 for Romania according to the Eurostat (SITC).

Year	Export						Import					
	Billions of kg		Percentage in total	Billions of euros		Percentage in total	Billions of kg		Percentage in total	Billions of euros		Percentage in total
	Food	Total		Food	Total		Food	Total		Food	Total	
1999	0,8	7,9	9,9%	0,2	5,8	4,2%	0,6	5,2	12,4%	0,4	6,7	6,1%
2000	0,5	7,9	6,7%	0,2	8,2	2,8%	1,0	5,1	20,0%	0,5	9,3	5,8%
2001	0,7	8,4	7,9%	0,3	9,6	3,5%	1,2	6,6	18,6%	0,7	11,7	6,3%
2002	0,6	10,0	6,4%	0,3	10,9	2,9%	1,0	6,4	15,0%	0,7	12,9	5,5%
2003	0,7	9,4	7,3%	0,4	11,9	3,1%	2,4	8,3	28,6%	0,9	14,5	6,1%
2004	0,9	10,8	8,4%	0,4	14,3	3,0%	1,4	9,0	15,2%	0,9	17,4	5,1%
2005	1,3	10,9	12,1%	0,5	15,8	3,1%	1,3	10,9	12,3%	1,1	20,6	5,2%
2006	1,6	11,5	14,2%	0,6	18,3	3,2%	1,5	12,6	12,1%	1,3	25,9	5,0%
2007	1,3	11,5	10,9%	0,8	21,4	3,8%	2,9	17,1	16,7%	2,3	36,6	6,4%
2008	2,3	12,6	18,1%	1,4	23,9	6,0%	3,8	18,8	20,4%	3,3	39,9	8,3%
2009	4,7	13,6	34,5%	1,8	21,7	8,1%	4,1	14,8	28,0%	3,0	28,5	10,4%
2010	5,1	15,6	32,7%	2,3	27,1	8,4%	4,2	16,8	24,9%	3,0	34,0	8,9%
2011	4,9	16,6	29,4%	2,9	32,3	9,0%	4,0	17,2	23,1%	3,3	40,0	8,3%
2012	4,3	14,6	29,5%	2,8	31,7	8,7%	4,5	18,3	24,4%	3,6	40,2	9,0%
2013	5,0	16,6	30,3%	3,1	34,5	8,9%	4,3	18,0	24,1%	3,8	41,9	9,1%
2014	6,6	19,0	34,8%	3,4	37,3	9,1%	4,5	18,3	24,8%	4,0	44,1	9,0%
2015	6,8	18,6	36,6%	3,8	40,2	9,5%	6,5	21,9	29,7%	4,7	48,6	9,6%
2016	6,8	18,9	36,2%	3,7	43,1	8,7%	7,5	23,7	31,5%	5,4	51,9	10,4%
2017	8,5	22,1	38,4%	4,2	47,5	8,8%	6,7	23,7	28,4%	5,9	57,3	10,3%
2018	10,2	25,8	39,4%	4,5	52,0	8,6%	6,1	24,0	25,4%	6,1	61,9	9,9%
Mean	3,7	14,1	26,1%	1,9	25,4	7,4%	3,5	14,8	23,5%	2,8	32,2	8,5%
Increase	1189,4%			1730,2%			840,4%			1381,4%		

Table S25. The intra-EU food trade in total trade in 1999-2018 for Slovakia according to the Eurostat (SITC).

Year	Export						Import					
	Billions of kg		Percentage in total	Billions of euros		Percentage in total	Billions of kg		Percentage in total	Billions of euros		Percentage in total
	Food	Total		Food	Total		Food	Total		Food	Total	
1999	1,2	17,5	6,9%	0,3	8,6	3,8%	0,9	10,6	8,8%	0,6	7,9	7,2%
2000	0,9	18,9	5,0%	0,4	11,6	3,1%	1,2	10,8	10,8%	0,6	9,7	6,6%
2001	1,0	20,2	4,9%	0,4	12,8	3,5%	1,5	12,3	11,9%	0,8	11,9	6,5%
2002	1,1	19,8	5,6%	0,5	13,7	3,5%	1,2	12,4	9,3%	0,8	12,8	6,1%
2003	1,3	20,5	6,3%	0,5	16,7	3,2%	1,2	13,4	9,1%	0,8	14,9	5,1%
2004	1,2	20,7	5,9%	0,8	19,4	3,9%	2,2	17,7	12,3%	1,1	18,9	5,7%
2005	1,7	21,0	8,0%	1,1	22,4	4,8%	1,8	16,7	10,8%	1,6	21,7	7,3%
2006	2,7	24,6	11,1%	1,3	29,1	4,6%	2,2	19,3	11,4%	1,8	27,0	6,5%
2007	2,3	27,9	8,2%	1,6	37,2	4,3%	2,5	22,0	11,6%	2,3	33,1	6,8%
2008	2,1	26,0	8,2%	1,7	41,5	4,2%	2,8	22,8	12,5%	2,6	36,8	7,2%
2009	2,7	23,0	11,6%	1,8	34,7	5,1%	2,5	19,5	13,0%	2,6	29,9	8,8%
2010	2,9	25,4	11,5%	2,1	42,3	5,0%	3,1	22,4	13,9%	3,1	36,5	8,5%
2011	3,4	27,4	12,2%	2,7	48,7	5,6%	3,3	22,3	14,8%	3,6	42,2	8,4%
2012	4,1	27,0	15,3%	3,4	52,7	6,5%	3,2	22,4	14,1%	3,8	44,4	8,5%
2013	4,1	29,7	13,8%	3,0	53,4	5,6%	3,0	22,7	13,2%	3,7	45,7	8,1%
2014	4,4	31,8	13,8%	2,5	54,7	4,6%	3,5	23,9	14,7%	3,6	46,9	7,7%
2015	4,7	31,9	14,8%	2,6	57,8	4,4%	3,5	25,0	14,2%	3,7	51,7	7,1%
2016	4,8	32,7	14,8%	2,6	59,4	4,4%	3,6	25,5	14,3%	3,9	54,0	7,3%
2017	4,8	33,2	14,4%	2,6	63,0	4,2%	3,7	27,2	13,6%	4,0	57,7	7,0%
2018	4,4	33,9	13,0%	2,6	67,6	3,9%	3,9	28,0	13,8%	4,2	62,8	6,7%
Mean	2,8	25,7	10,9%	1,7	37,4	4,6%	2,5	19,8	12,8%	2,5	33,3	7,4%
Increase	264,3%			698,0%			314,8%			642,2%		

Table S26. The intra-EU food trade in total trade in 1999-2018 for Slovenia according to the Eurostat (SITC).

Year	Export						Import					
	Billions of kg		Percentage in total	Billions of euros		Percentage in total	Billions of kg		Percentage in total	Billions of euros		Percentage in total
	Food	Total		Food	Total		Food	Total		Food	Total	
1999	2,3	6,1	38,2%	0,2	6,6	2,6%	0,9	9,1	9,8%	0,5	7,8	6,1%
2000	2,6	6,7	38,7%	0,2	7,6	2,1%	1,0	9,4	10,7%	0,5	8,9	5,8%
2001	2,2	6,6	33,1%	0,2	8,2	1,9%	2,4	10,7	22,1%	0,6	9,2	6,0%
2002	2,1	6,7	31,6%	0,2	8,5	2,0%	2,3	10,6	22,0%	0,6	9,4	6,0%
2003	2,5	7,4	34,4%	0,2	8,7	2,2%	2,9	11,6	24,6%	0,6	9,8	5,8%
2004	2,3	7,7	30,1%	0,2	10,1	2,0%	2,9	12,8	23,0%	0,7	12,2	5,9%
2005	2,4	8,9	26,4%	0,4	12,0	2,9%	2,3	12,0	19,3%	0,9	13,7	6,3%
2006	3,2	10,9	29,7%	0,5	14,3	3,8%	2,5	14,1	17,9%	1,0	15,8	6,3%
2007	3,2	11,8	27,6%	0,7	17,0	4,1%	2,9	17,7	16,4%	1,2	18,1	6,7%
2008	4,0	12,7	31,8%	0,8	17,7	4,5%	2,9	16,0	18,4%	1,4	19,0	7,1%
2009	4,1	11,8	34,9%	0,8	14,4	5,3%	1,5	12,7	12,2%	1,3	14,3	9,2%
2010	3,9	12,7	31,1%	1,0	17,1	5,8%	1,5	12,7	12,0%	1,4	16,5	8,4%
2011	3,9	13,6	28,4%	1,0	19,3	5,4%	1,6	12,8	12,6%	1,5	18,4	8,2%
2012	4,6	14,1	32,8%	1,3	18,8	6,8%	1,7	12,7	13,5%	1,6	18,0	8,8%
2013	4,0	14,2	28,3%	1,1	19,2	5,9%	2,0	12,7	15,5%	1,6	17,6	9,2%
2014	3,4	15,2	22,3%	1,0	20,4	5,0%	2,0	12,9	15,2%	1,6	17,7	9,3%
2015	3,8	16,6	22,7%	1,2	21,9	5,4%	2,2	14,6	15,0%	1,7	18,8	9,1%
2016	3,7	17,2	21,5%	1,2	22,4	5,6%	2,1	15,7	13,1%	1,8	19,6	9,1%
2017	3,8	18,3	21,0%	1,3	25,8	5,1%	2,5	16,0	15,5%	1,9	22,1	8,6%
2018	4,2	19,2	22,1%	1,5	28,5	5,2%	2,2	16,0	13,7%	2,0	24,1	8,3%
Mean	3,3	11,9	27,9%	0,7	15,9	4,7%	2,1	13,1	16,1%	1,2	15,5	7,8%
Increase	83,8%			783,4%			147,3%			324,5%		

Table S27. The intra-EU food trade in total trade in 1999-2018 for Spain according to the Eurostat (SITC).

Year	Export						Import					
	Billions of kg		Percentage in total	Billions of euros		Percentage in total	Billions of kg		Percentage in total	Billions of euros		Percentage in total
	Food	Total		Food	Total		Food	Total		Food	Total	
1999	12,1	49,3	24,6%	11,1	71,6	15,5%	10,2	55,2	18,4%	7,6	88,8	8,6%
2000	14,4	58,7	24,6%	13,6	90,0	15,1%	11,2	68,2	16,4%	9,0	114,5	7,8%
2001	15,9	58,8	27,0%	15,5	95,9	16,2%	11,9	72,3	16,5%	10,2	118,6	8,6%
2002	16,5	61,6	26,7%	16,5	98,6	16,8%	13,4	76,1	17,6%	10,8	120,8	8,9%
2003	17,5	65,3	26,8%	17,5	103,0	17,0%	12,8	79,7	16,1%	11,2	127,9	8,8%
2004	17,3	67,2	25,7%	17,7	108,1	16,4%	14,1	82,4	17,1%	12,2	141,1	8,6%
2005	16,6	65,3	25,4%	18,2	110,4	16,5%	19,7	86,3	22,9%	13,4	149,0	9,0%
2006	17,1	56,0	30,5%	19,1	119,3	16,0%	17,6	76,4	23,1%	13,6	161,8	8,4%
2007	17,8	59,9	29,7%	20,3	129,7	15,6%	12,9	76,6	16,8%	14,4	179,0	8,1%
2008	20,9	80,4	26,0%	22,2	131,4	16,9%	14,2	95,2	15,0%	15,2	169,6	9,0%
2009	19,9	69,3	28,8%	21,3	112,7	18,9%	19,8	79,0	25,1%	15,3	131,1	11,7%
2010	20,6	75,2	27,4%	23,4	130,4	18,0%	18,3	81,1	22,6%	16,1	145,6	11,0%
2011	22,3	78,2	28,6%	25,0	144,8	17,2%	16,6	73,6	22,5%	16,9	154,0	10,9%
2012	22,9	79,3	28,9%	26,6	142,6	18,6%	16,4	68,5	23,9%	16,8	142,4	11,8%
2013	23,1	82,8	27,9%	28,2	147,6	19,1%	15,3	67,6	22,7%	16,7	141,7	11,8%
2014	25,0	88,5	28,2%	29,0	153,0	18,9%	18,1	77,3	23,4%	17,1	154,8	11,1%
2015	25,4	91,2	27,9%	31,6	163,3	19,3%	20,3	80,1	25,3%	18,4	170,8	10,8%
2016	25,2	92,5	27,3%	33,0	173,0	19,1%	21,6	79,8	27,1%	19,3	173,9	11,1%
2017	25,8	100,1	25,8%	35,2	185,0	19,0%	20,9	82,8	25,2%	20,5	186,0	11,0%
2018	26,0	103,5	25,1%	36,0	191,8	18,8%	19,2	80,9	23,7%	21,0	194,3	10,8%
Mean	20,1	74,2	27,1%	23,0	130,1	17,7%	16,2	77,0	21,1%	14,8	148,3	10,0%
Increase	114,4%			225,4%			88,7%			175,4%		

Table S28. The intra-EU food trade in total trade in 1999-2018 for Sweden according to the Eurostat (SITC).

Year	Export						Import					
	Billions of kg		Percentage in total	Billions of euros		Percentage in total	Billions of kg		Percentage in total	Billions of euros		Percentage in total
	Food	Total		Food	Total		Food	Total		Food	Total	
1999	1,3	50,6	2,6%	1,3	48,2	2,7%	2,9	35,5	8,1%	3,1	42,6	7,2%
2000	1,5	52,4	2,8%	1,5	55,1	2,7%	2,9	39,3	7,4%	3,2	50,3	6,4%
2001	1,7	50,5	3,4%	1,6	48,3	3,2%	3,1	36,8	8,6%	3,5	46,7	7,5%
2002	1,4	50,1	2,8%	1,7	49,3	3,5%	3,4	40,6	8,3%	4,0	48,3	8,2%
2003	1,9	52,6	3,7%	1,9	51,9	3,7%	4,2	43,8	9,5%	4,2	51,3	8,3%
2004	2,0	54,9	3,7%	2,2	57,1	3,8%	4,3	44,9	9,6%	4,4	56,6	7,9%
2005	1,8	56,8	3,1%	2,5	61,9	4,0%	4,2	44,7	9,5%	4,7	63,3	7,5%
2006	2,2	61,2	3,6%	3,0	70,5	4,2%	4,9	45,2	10,8%	5,2	70,8	7,4%
2007	2,6	61,8	4,2%	3,2	75,1	4,2%	4,9	48,4	10,0%	5,6	79,5	7,1%
2008	2,7	60,9	4,4%	3,5	74,6	4,7%	5,4	48,2	11,2%	6,2	79,3	7,9%
2009	2,5	50,7	5,0%	3,4	54,6	6,3%	5,6	38,6	14,6%	5,8	58,4	10,0%
2010	2,7	56,7	4,8%	4,2	68,0	6,2%	6,1	45,9	13,3%	6,4	75,4	8,6%
2011	2,6	56,8	4,6%	4,5	74,8	6,0%	4,5	48,1	9,4%	7,2	86,7	8,3%
2012	2,9	59,1	4,8%	4,8	75,8	6,3%	4,8	49,1	9,8%	7,8	86,2	9,1%
2013	2,7	57,0	4,8%	5,3	72,3	7,3%	4,8	45,9	10,5%	8,2	83,3	9,9%
2014	3,2	59,5	5,4%	5,4	71,9	7,5%	4,9	45,8	10,6%	8,2	84,0	9,7%
2015	3,6	60,0	5,9%	5,9	73,5	8,1%	5,0	45,6	11,0%	8,6	87,4	9,9%
2016	3,7	62,2	6,0%	6,7	74,1	9,0%	5,2	49,5	10,4%	9,0	90,6	10,0%
2017	3,6	64,3	5,6%	6,6	79,5	8,3%	5,2	50,3	10,3%	9,4	97,6	9,6%
2018	3,3	62,7	5,2%	6,9	83,2	8,3%	5,6	54,4	10,3%	9,5	101,2	9,4%
Mean	2,5	57,0	4,4%	3,8	66,0	5,8%	4,6	45,0	10,2%	6,2	72,0	8,7%
Increase	148,5%			424,2%			94,5%			211,7%		

Table S29. The intra-EU food trade in total trade in 1999-2018 for the United Kingdom according to the Eurostat (SITC).

Year	Export						Import					
	Billions of kg		Percentage in total	Billions of euros		Percentage in total	Billions of kg		Percentage in total	Billions of euros		Percentage in total
	Food	Total		Food	Total		Food	Total		Food	Total	
1999	9,1	137,3	6,7%	9,6	152,4	6,3%	14,9	82,1	18,1%	17,4	163,3	10,7%
2000	9,5	140,2	6,8%	10,0	181,0	5,5%	15,2	86,0	17,7%	18,3	187,2	9,8%
2001	6,8	144,0	4,7%	9,4	179,7	5,2%	17,3	92,8	18,7%	19,1	190,5	10,0%
2002	8,3	144,2	5,7%	10,1	180,9	5,6%	17,8	97,7	18,2%	20,4	200,7	10,1%
2003	10,6	132,5	8,0%	10,3	159,4	6,5%	19,2	96,5	19,9%	20,8	192,9	10,8%
2004	9,2	130,0	7,1%	10,4	163,8	6,3%	19,6	101,3	19,3%	22,3	207,1	10,8%
2005	9,8	124,6	7,9%	10,4	172,8	6,0%	20,2	97,2	20,8%	23,9	212,9	11,2%
2006	9,9	128,7	7,7%	10,9	218,3	5,0%	20,3	100,0	20,3%	25,6	235,8	10,9%
2007	9,7	126,1	7,7%	12,0	181,0	6,6%	20,2	102,7	19,7%	27,3	242,6	11,2%
2008	10,3	120,7	8,6%	12,2	173,7	7,0%	20,5	94,8	21,6%	27,6	223,2	12,3%
2009	10,4	107,2	9,7%	11,4	136,3	8,4%	20,1	82,3	24,4%	25,7	180,2	14,2%
2010	11,4	121,3	9,4%	12,8	161,0	8,0%	20,6	94,5	21,8%	28,1	213,6	13,2%
2011	10,7	120,4	8,9%	13,9	177,6	7,8%	20,8	100,3	20,7%	30,2	230,6	13,1%
2012	10,4	111,6	9,3%	14,4	179,3	8,0%	23,1	104,1	22,2%	33,5	251,4	13,3%
2013	9,8	109,5	8,9%	14,1	171,1	8,3%	26,2	112,5	23,3%	34,2	251,9	13,6%
2014	10,1	110,6	9,1%	14,8	178,1	8,3%	24,6	111,1	22,1%	35,4	271,9	13,0%
2015	11,1	108,2	10,2%	15,7	183,8	8,5%	25,8	112,6	22,9%	39,1	301,2	13,0%
2016	11,8	105,4	11,2%	15,3	174,7	8,7%	25,8	119,8	21,5%	37,0	288,8	12,8%
2017	10,4	106,8	9,7%	15,6	185,3	8,4%	26,9	122,3	22,0%	38,0	293,4	13,0%
2018	10,6	107,2	9,9%	16,2	192,8	8,4%	26,8	123,7	21,7%	38,6	299,4	12,9%
Mean	10,0	121,8	8,2%	12,5	175,2	7,1%	21,3	101,7	20,9%	28,1	231,9	12,1%
Increase	16,2%			67,9%			80,4%			121,6%		

Table S30. The total food production in billions of US$ in 1999-2018 for EU countries according to the Faostat

Year	Country													
	**Austria**	**Belgium**	**Bulgaria**	**Croatia**	**Cyprus**	**Czech Republic**	**Denmark**	**Estonia**	**Finland**	**France**	**Germany**	**Greece**	**Hungary**	**Ireland**
1999	4,1	6,9	1,9	1,4	0,5	2,9	6,6	0,3	2,3	50,0	30,7	10,6	3,7	4,4
2000	3,7	6,0	1,7	0,9	0,5	2,8	5,7	0,3	2,0	43,3	28,2	9,6	3,6	4,0
2001	3,9	6,2	1,6	1,0	0,5	3,2	6,0	0,3	2,0	41,2	29,4	9,6	4,2	4,1
2002	3,9	6,3	1,6	1,2	0,5	3,1	5,6	0,3	2,2	43,3	27,9	10,7	4,3	4,0
2003	4,8	8,0	1,8	1,2	0,6	3,2	6,5	0,4	2,5	50,1	33,5	12,5	4,7	5,0
2004	5,2	8,4	2,8	1,5	0,7	4,4	7,5	0,5	2,8	53,9	40,5	13,7	6,1	5,6
2005	5,0	8,5	2,2	1,6	0,7	4,0	7,2	0,6	2,8	53,7	37,8	15,1	5,5	5,2
2006	5,4	9,1	2,4	1,7	0,7	4,1	7,5	0,6	2,8	57,6	41,3	15,2	5,7	5,4
2007	6,9	10,1	2,9	2,1	0,8	5,3	9,0	0,8	3,6	68,3	52,0	17,0	7,4	6,6
2008	7,6	10,3	4,5	2,5	0,9	7,0	10,6	0,8	4,2	74,5	60,5	19,5	9,4	7,1
2009	6,4	9,1	3,2	2,0	0,8	4,4	8,1	0,6	3,4	63,4	45,5	18,5	6,4	5,4
2010	6,5	9,3	3,8	2,0	0,8	4,3	8,3	0,7	3,1	69,3	48,8	18,3	6,3	6,2
2011	7,7	10,4	4,7	2,3	0,8	6,1	10,3	0,9	3,9	78,2	60,1	18,6	8,6	7,9
2012	7,3	9,9	4,5	2,2	0,7	5,1	10,4	0,9	3,7	76,0	58,0	17,7	7,7	7,3
2013	7,4	11,0	4,9	2,1	0,7	5,9	10,8	1,0	4,2	75,9	62,8	15,2	8,0	8,2
2014	7,2	9,9	4,8	1,9	0,7	6,2	10,6	1,0	3,8	76,7	60,4	17,3	8,2	8,2
2015	5,8	7,8	3,8	1,7	0,6	4,5	7,9	0,8	3,0	65,4	45,0	15,6	6,8	6,8
2016	5,9	7,8	3,8	1,8	0,6	4,5	7,7	0,6	2,9	63,5	43,5	14,7	7,3	6,7
2017	6,4	8,4	4,0	1,8	0,7	4,8	9,1	0,8	3,0	66,1	49,3	15,7	7,6	8,0
2018	7,1	8,9	4,2	2,0	0,7	5,1	8,4	0,8	3,3	74,4	49,9	12,1	7,5	8,3
Percentage increase	71,5%	29,4%	124,0%	40,1%	25,3%	75,0%	27,6%	195,4%	44,4%	49,0%	62,6%	14,5%	99,5%	87,9%

Year	Country													
	**Italy**	**Latvia**	**Lithuania**	**Luxembourg**	**Malta**	**Netherlands**	**Poland**	**Portugal**	**Romania**	**Slovakia**	**Slovenia**	**Spain**	**Sweden**	**United Kingdom**
1999	40,3	0,4	1,0	-	0,1	12,3	11,1	5,0	7,4	1,3	0,7	27,4	3,3	19,1
2000	34,3	0,4	0,9	0,2	0,1	10,4	11,2	4,4	6,8	1,1	0,6	27,3	3,0	16,6
2001	33,6	0,4	0,9	0,2	0,1	10,8	12,4	4,2	8,6	1,2	0,6	27,7	2,7	16,3
2002	33,8	0,5	1,0	0,2	0,1	10,8	11,6	4,7	8,1	1,3	0,7	28,4	2,8	16,1
2003	40,8	0,5	1,2	0,2	0,1	13,0	12,0	5,7	10,6	1,4	0,8	38,6	3,3	18,7
2004	48,2	0,6	1,3	0,3	0,1	13,9	15,1	6,8	14,4	1,8	0,9	39,6	3,6	21,6
2005	44,4	0,7	1,5	0,2	0,1	13,6	15,5	6,5	13,1	1,8	0,8	37,9	3,3	20,9
2006	47,5	0,8	1,4	0,2	0,1	14,3	16,5	6,1	13,8	1,8	0,9	39,9	3,4	22,3
2007	53,4	1,2	2,4	0,3	0,1	16,8	23,7	6,8	15,5	2,2	1,0	47,9	4,4	27,3
2008	60,2	1,3	2,7	0,3	0,2	17,8	26,4	8,0	20,7	3,0	1,2	50,8	5,0	31,7
2009	53,0	0,9	1,9	0,2	0,2	15,2	19,5	7,1	16,0	2,0	0,9	42,6	3,6	24,8
2010	50,5	1,0	2,0	0,3	0,1	16,5	21,9	7,3	16,7	1,9	0,9	45,4	4,2	26,7
2011	55,3	1,3	2,8	0,3	0,2	17,5	27,0	7,0	22,0	2,6	1,0	46,9	5,0	32,7
2012	54,0	1,5	3,0	0,3	0,1	16,9	26,7	7,1	16,1	2,1	0,9	42,1	4,9	31,5
2013	54,7	1,4	2,9	0,3	0,2	19,5	27,2	7,4	19,8	2,2	1,0	51,2	5,1	33,7
2014	51,1	1,3	2,7	0,3	0,2	18,7	26,8	7,3	19,3	2,3	1,0	47,0	5,0	35,1
2015	45,5	1,2	2,4	0,2	0,1	15,5	20,8	7,2	15,1	1,6	0,8	41,4	4,1	29,5
2016	44,3	1,1	2,1	0,2	0,1	15,8	20,0	6,8	15,0	1,8	0,8	43,0	3,9	25,6
2017	46,4	1,3	2,4	0,3	0,1	17,4	24,3	7,4	17,4	1,7	0,9	44,6	4,2	27,2
2018	49,8	1,3	2,3	0,3	0,1	15,7	24,7	6,9	19,3	1,9	1,0	51,8	3,9	15,6
Percentage increase	23,6%	217,1%	131,0%	81,4%	0,7%	27,9%	122,3%	38,6%	162,7%	45,7%	39,0%	88,9%	16,7%	-18,3%

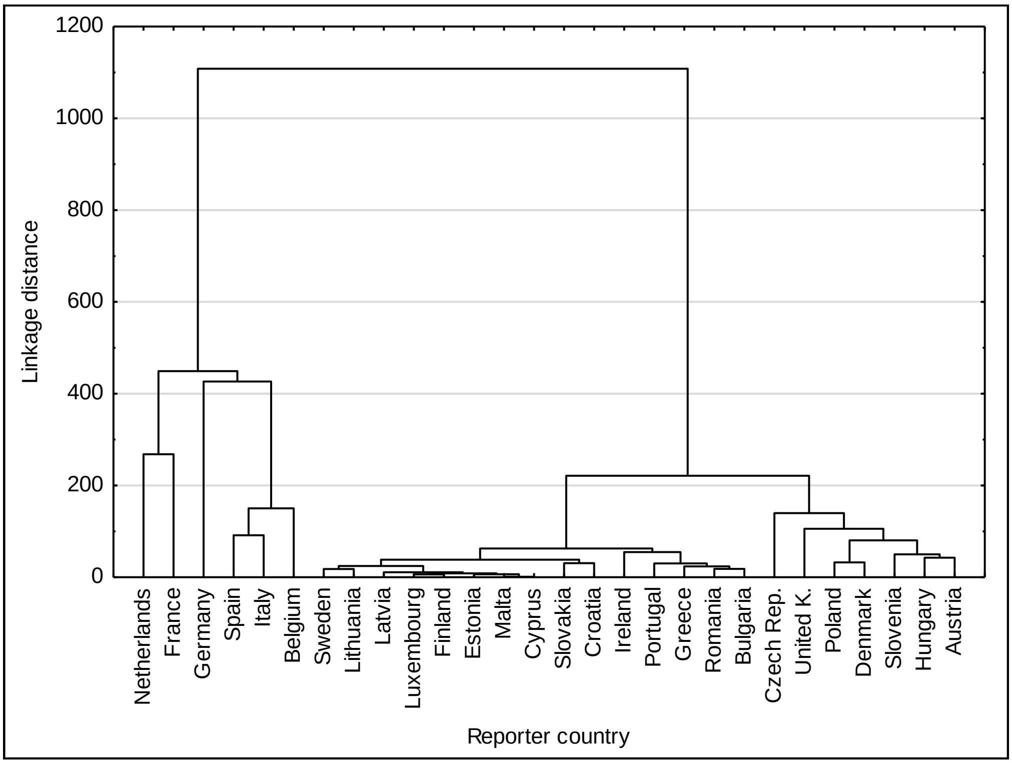

(a) joining

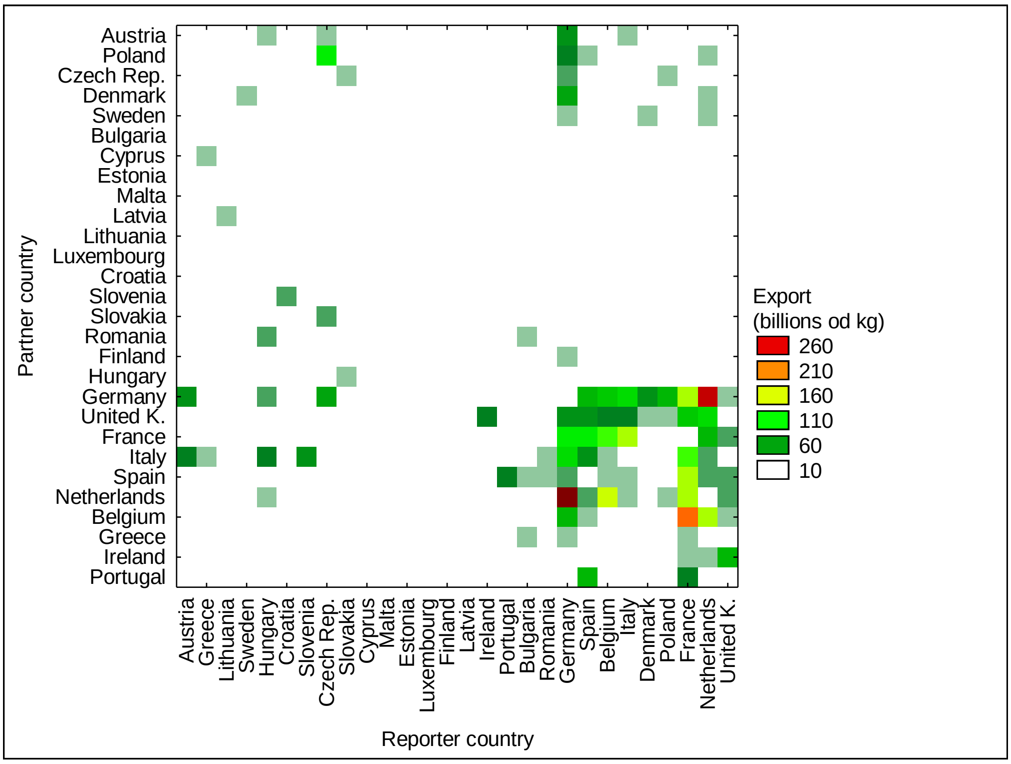

(b) two-way joining

Figure S1. Total export of food in the European Union in 1999-2018 (billions of kg)

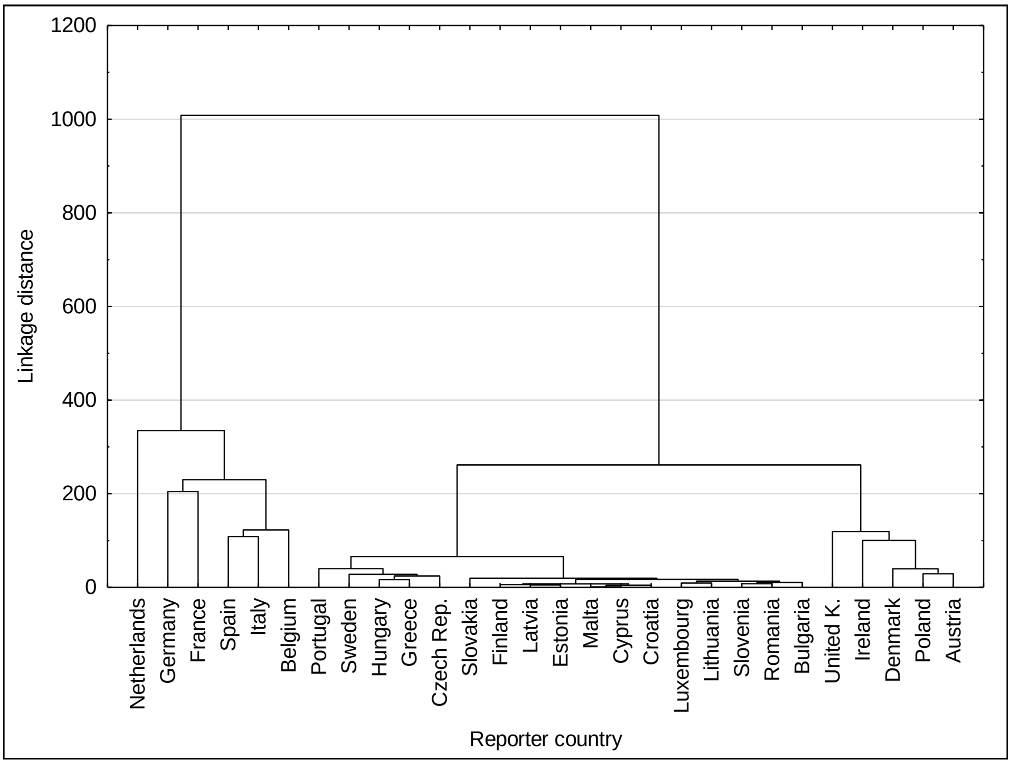

(a) joining

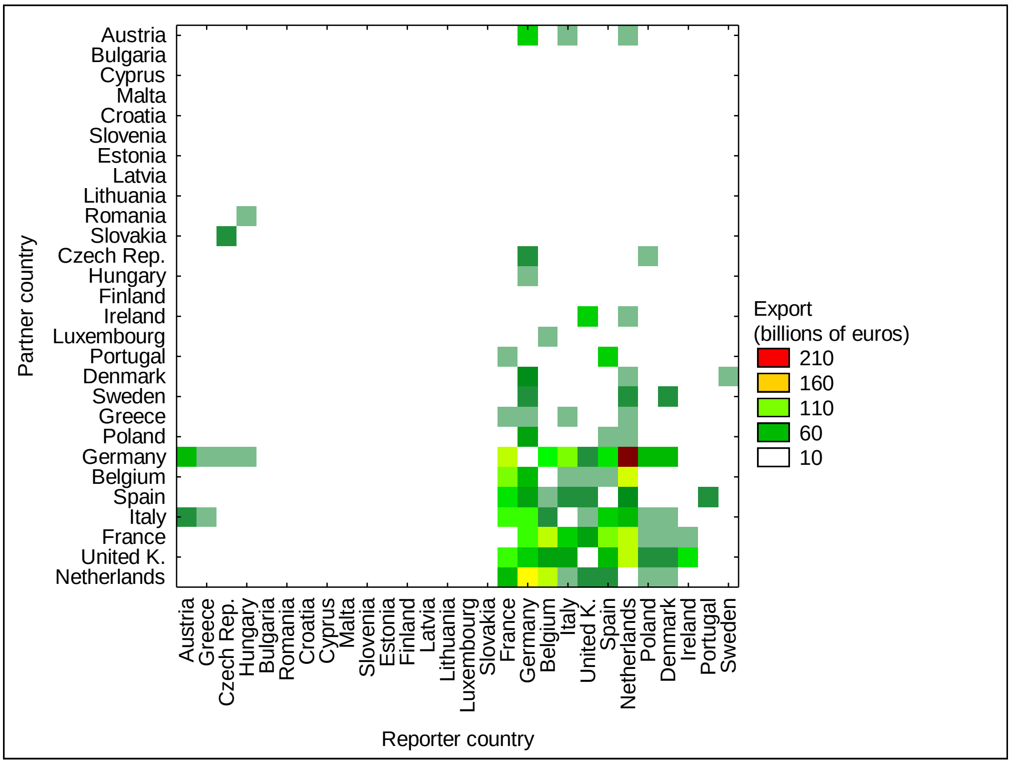

(b) two-way joining

Figure S2. Total export of food in the European Union in 1999-2018 (billions of euros)

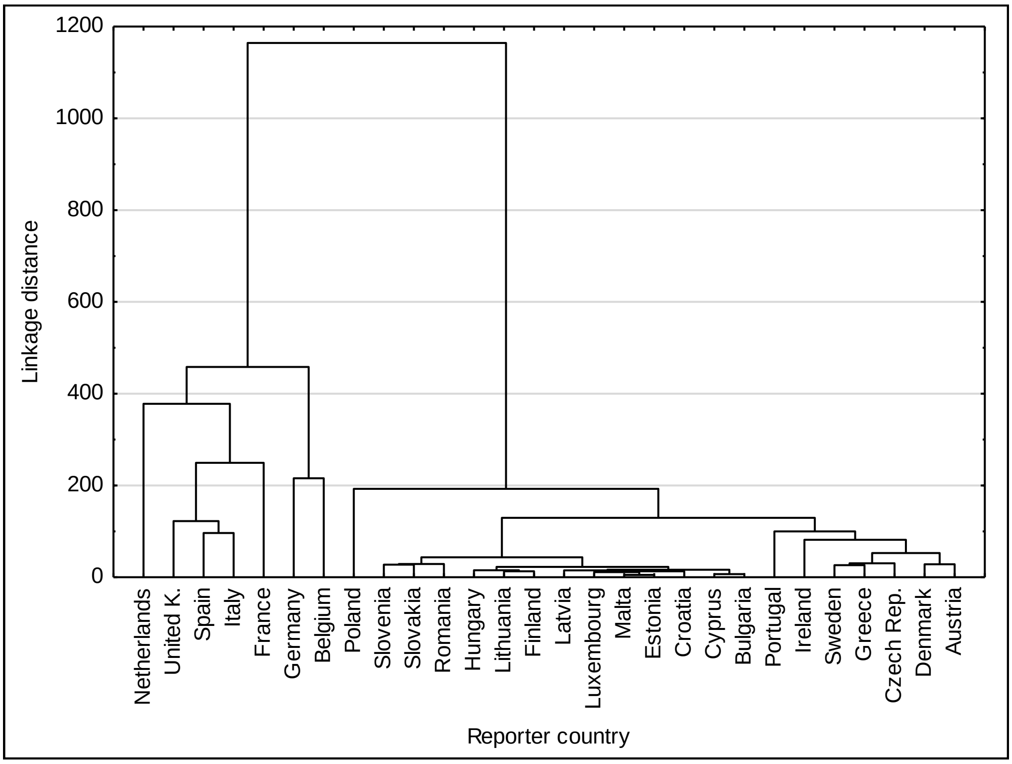

(a) joining

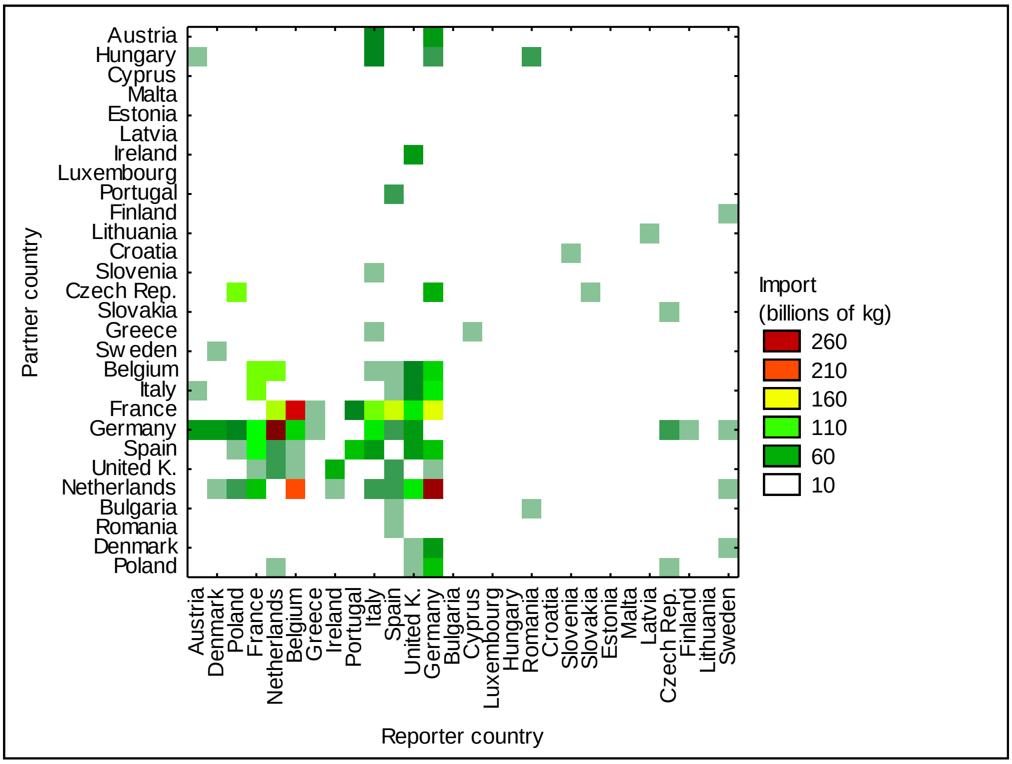

(b) two-way joining

Figure S3. Total import of food in the European Union in 1999-2018 (billions of kg)

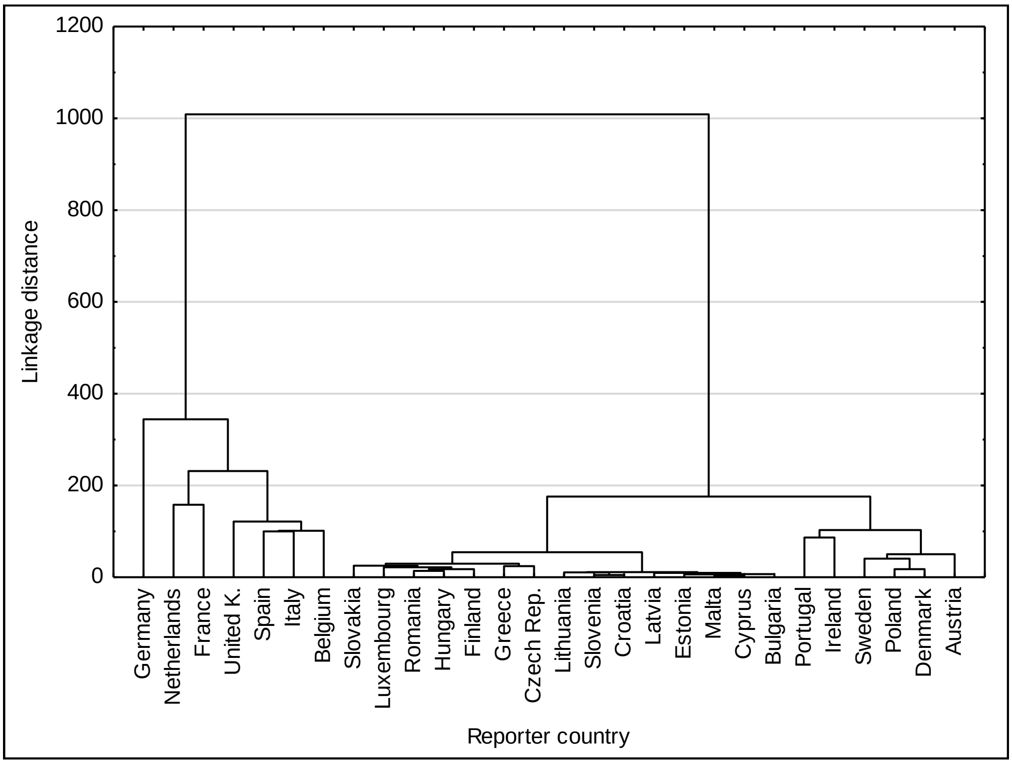

(a) joining

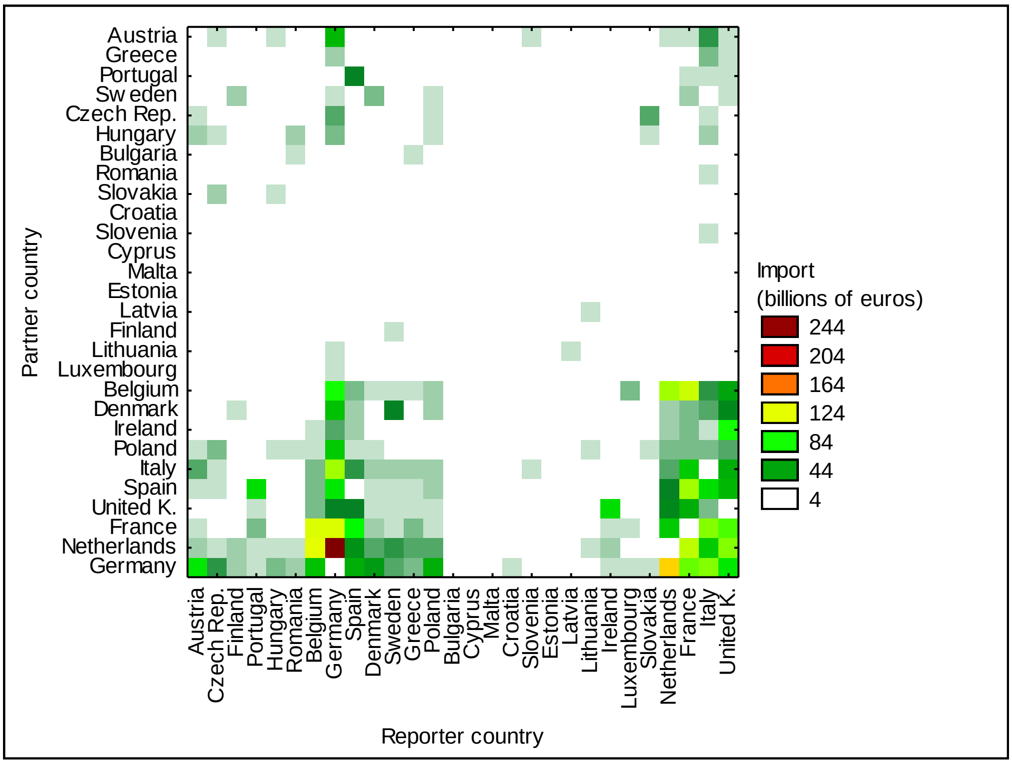

(b) two-way joining

Figure S4. Total import of food in the European Union in 1999-2018 (billions of euros)

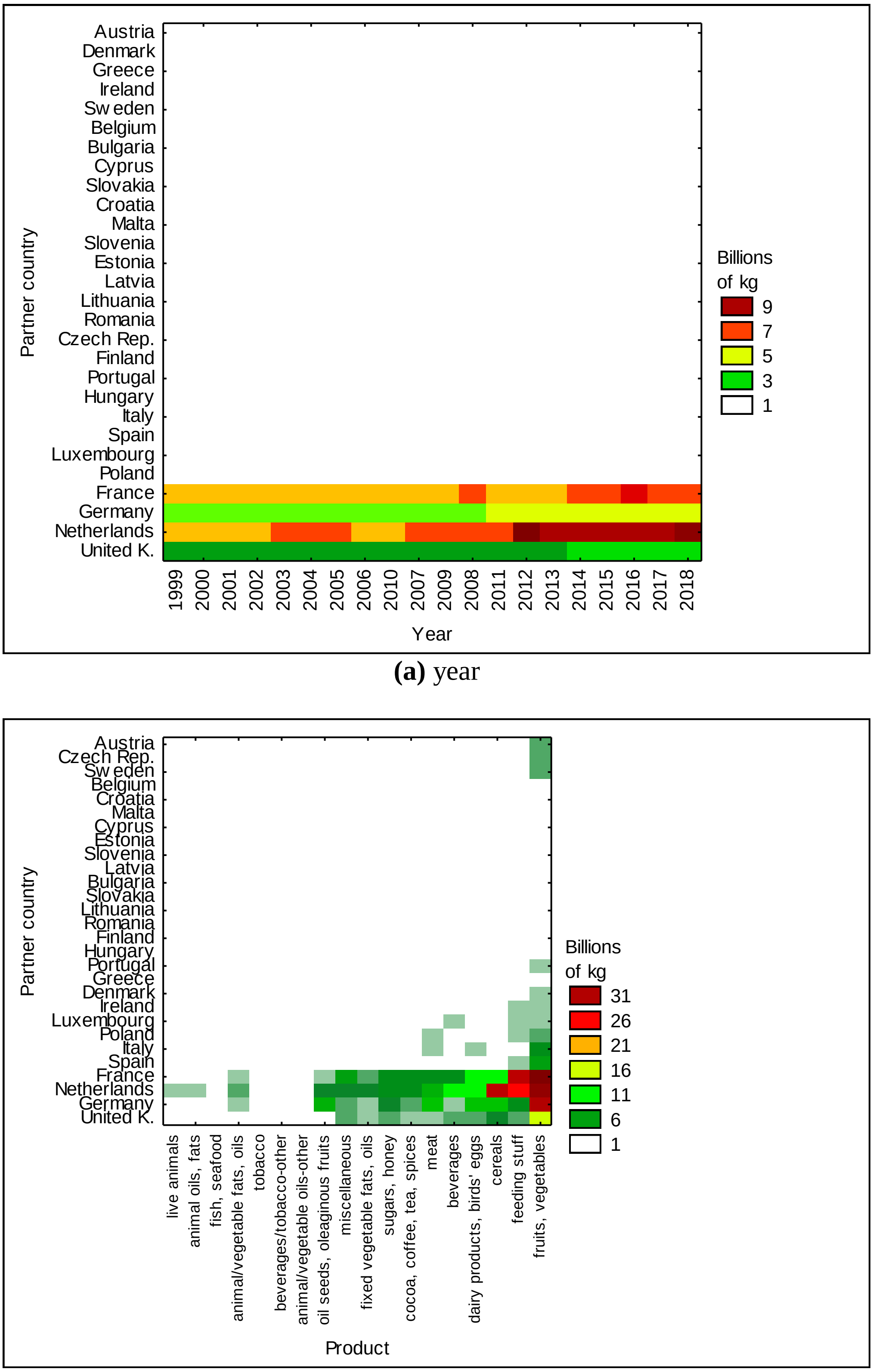

(a) year

(b) product

Figure S5. Export of food from Belgium in 1999-2018 (billions of kg)

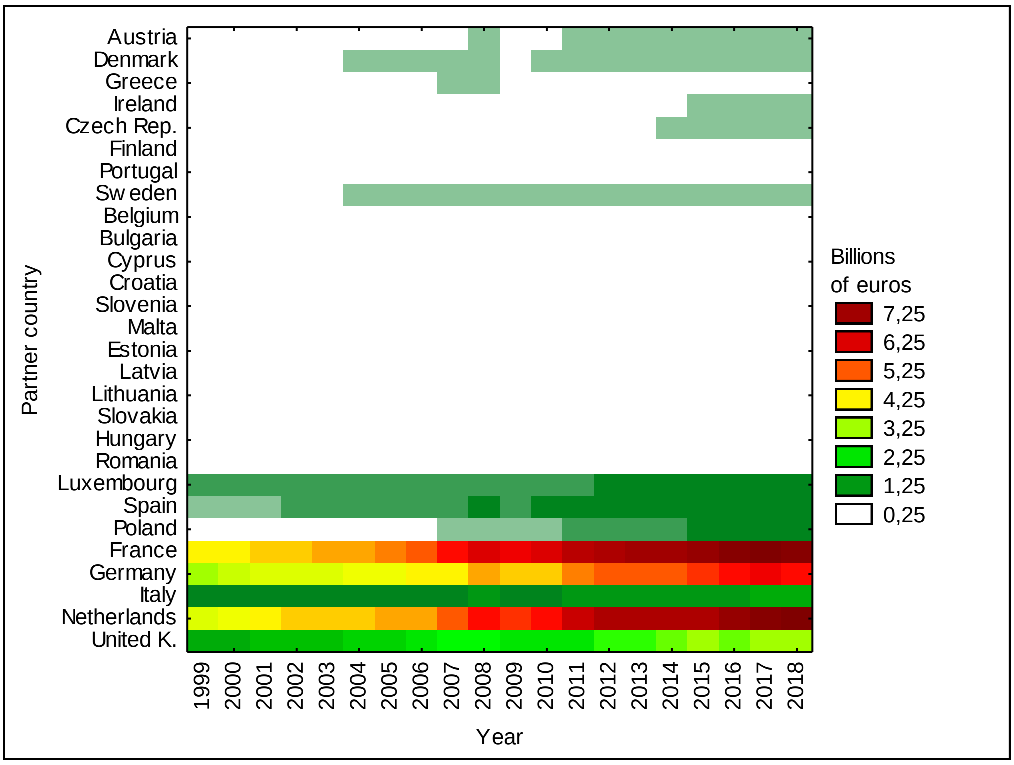

(a) year

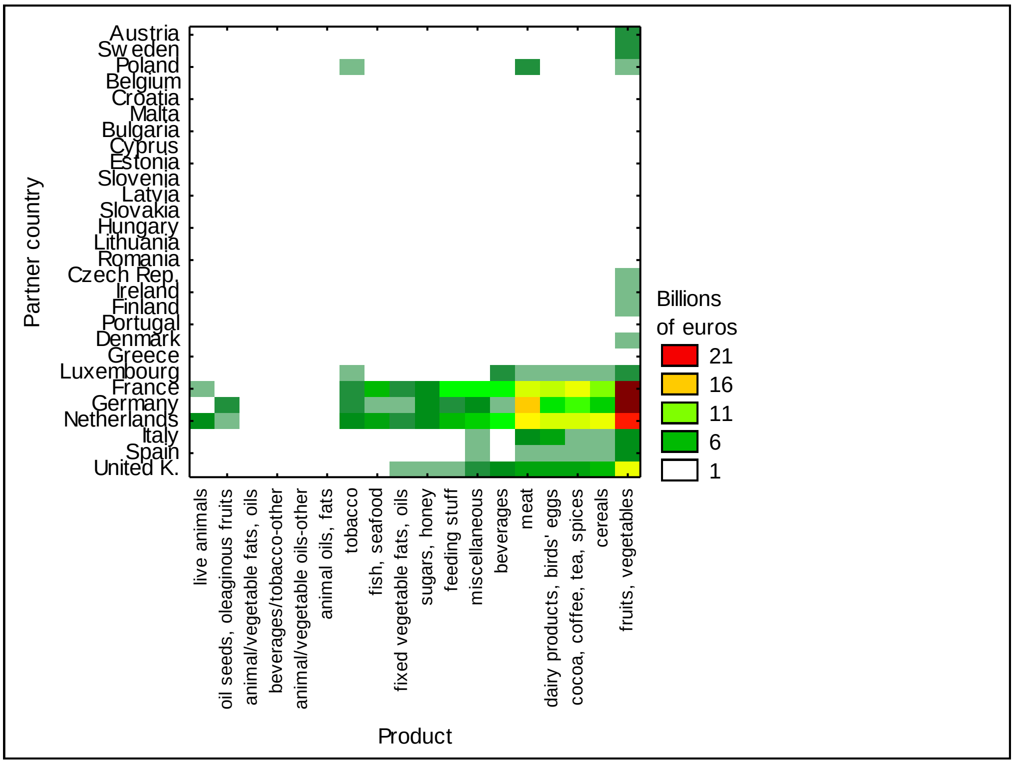

(b) product

Figure S6. Export of food from Belgium in 1999-2018 (billions of euros)

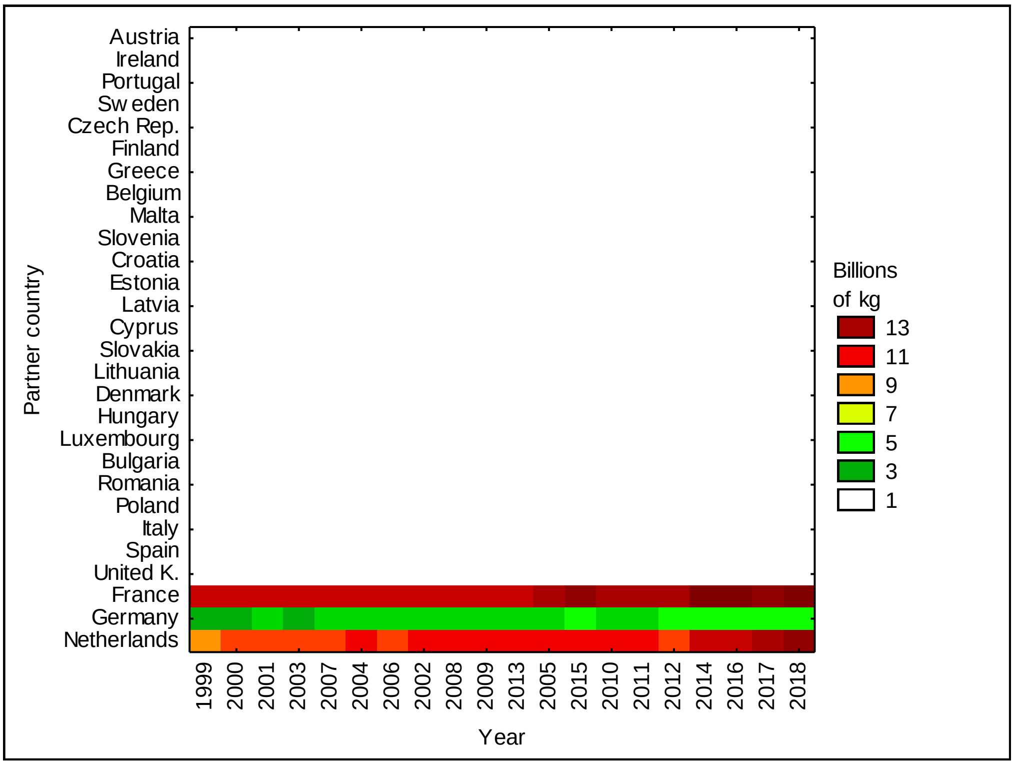

(a) year

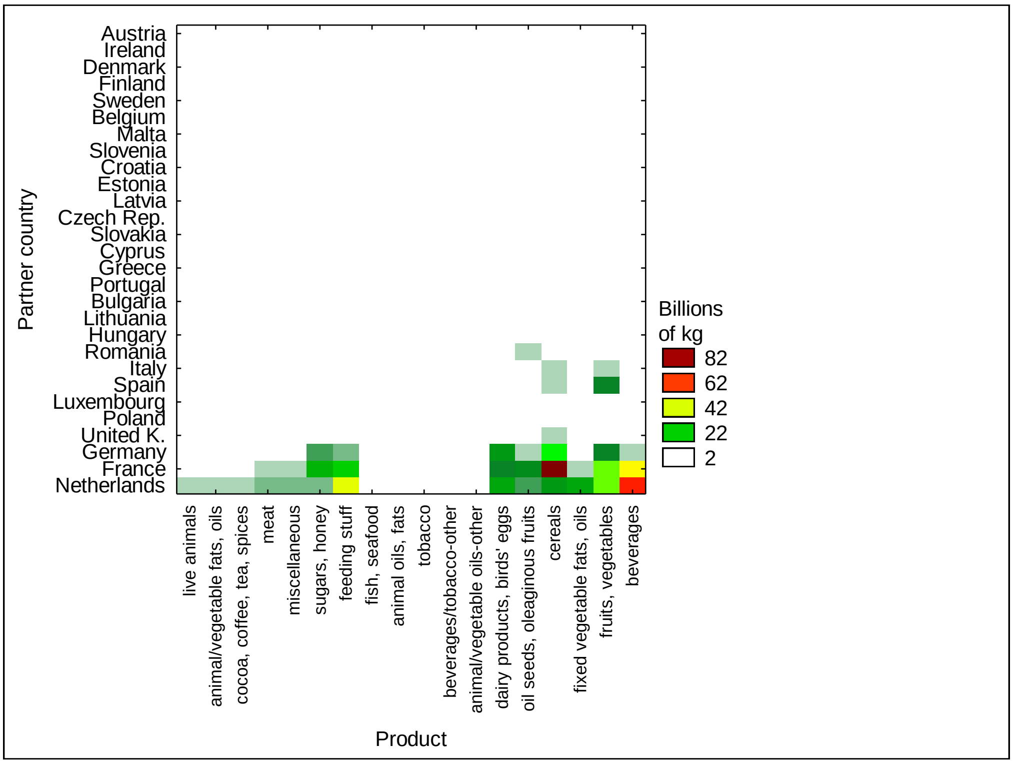

(b) product

Figure S7. Import of food to Belgium in 1999-2018 (billions of kg)

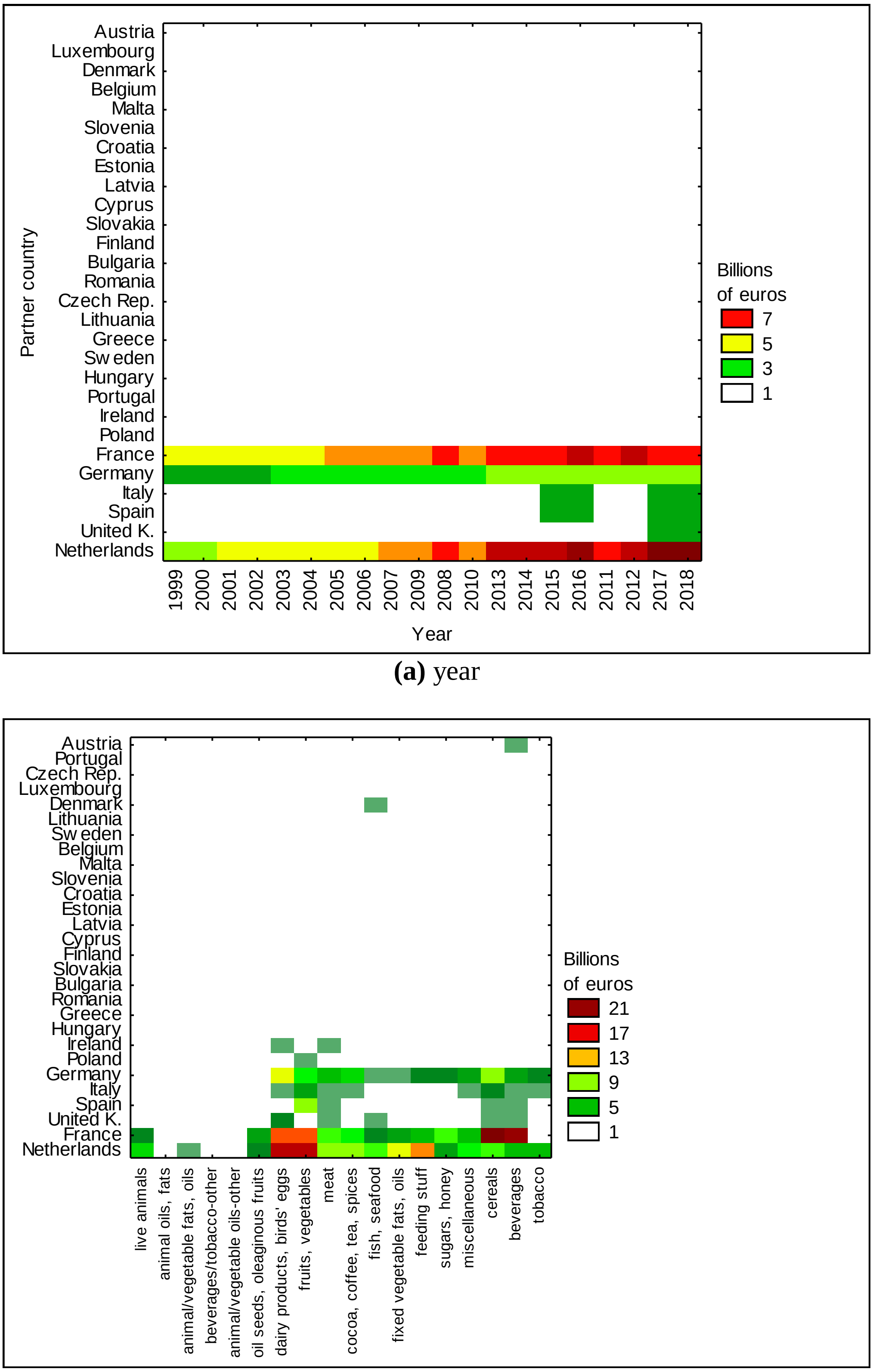

(a) year

(b) product

Figure S8. Import of food to Belgium in 1999-2018 (billions of euros)

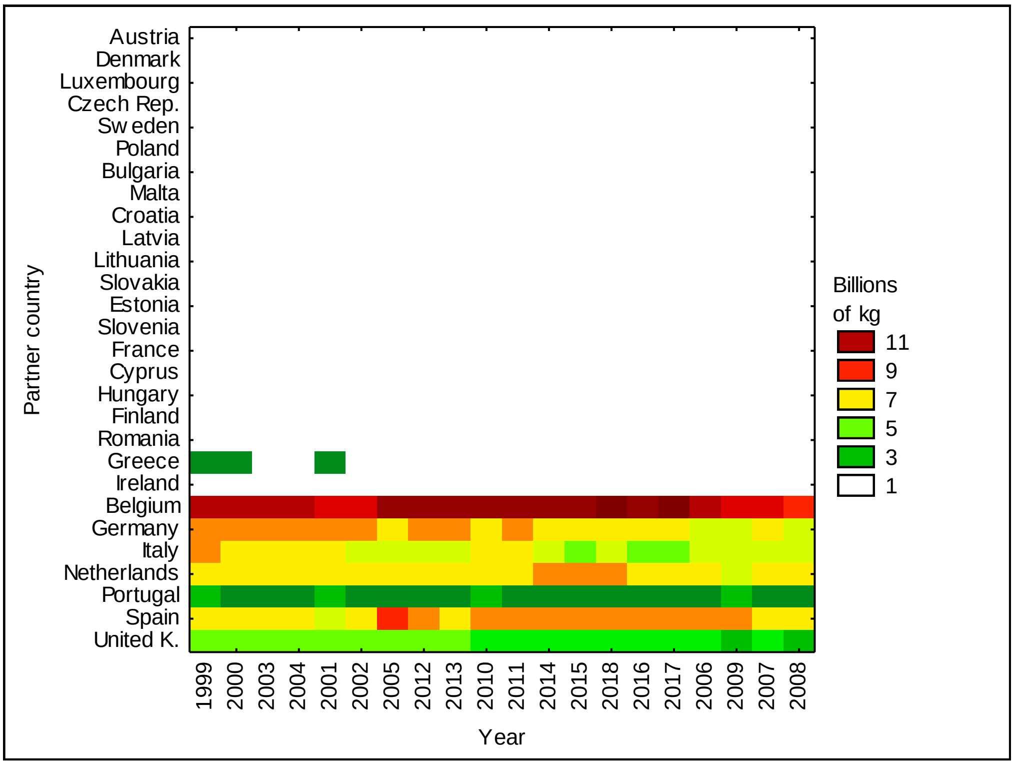

(a) year

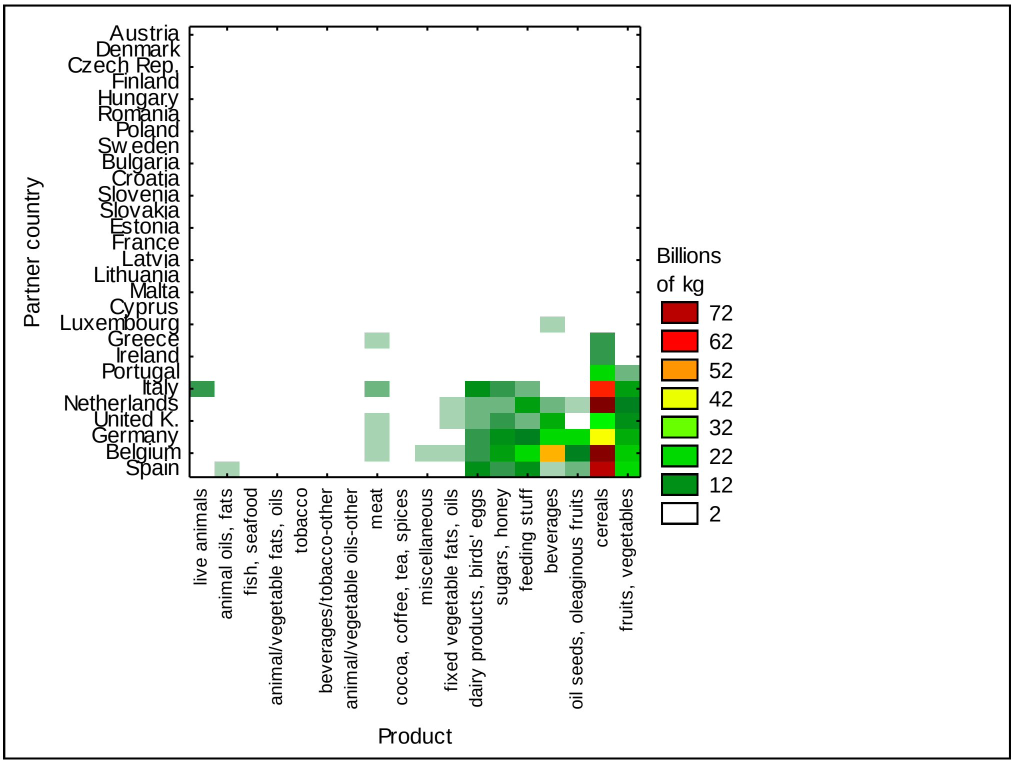

(b) product

Figure S9. Export of food from France in 1999-2018 (billions of kg)

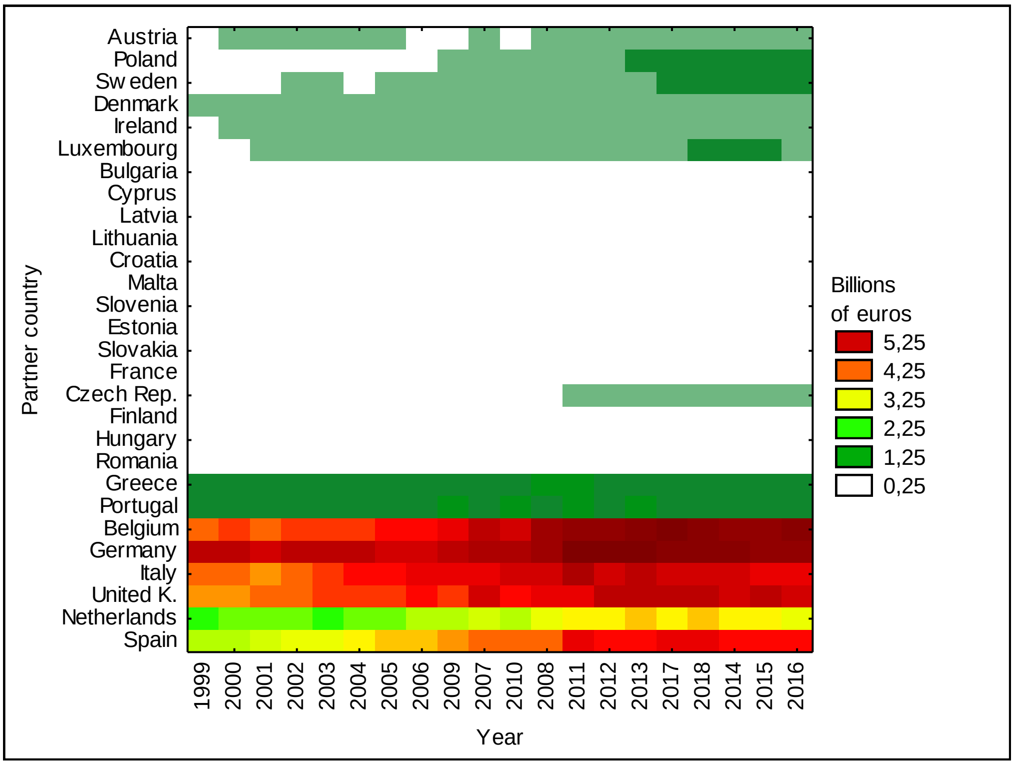

(a) year

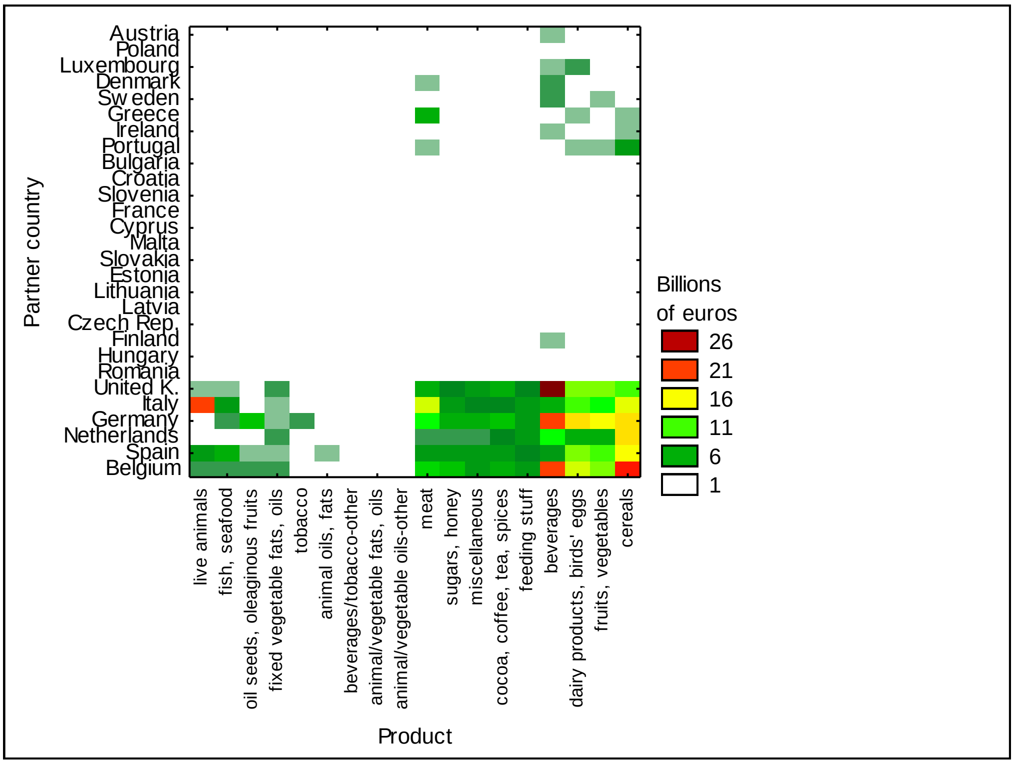

(b) product

Figure S10. Export of food from France in 1999-2018 (billions of euros)

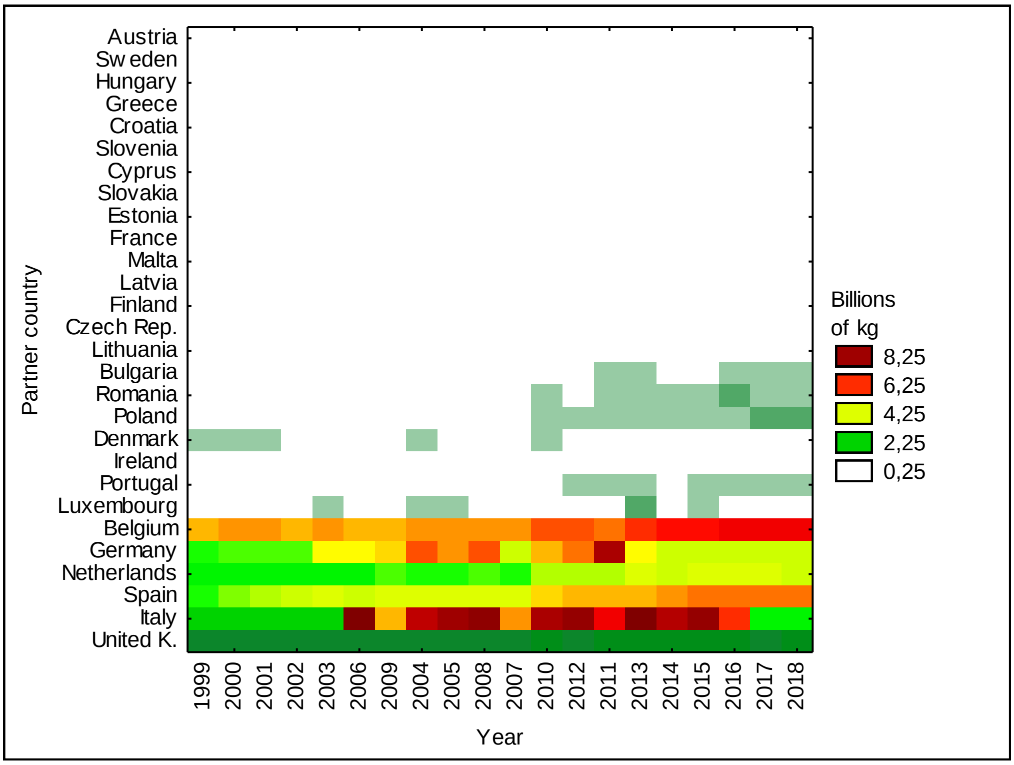

(a) year

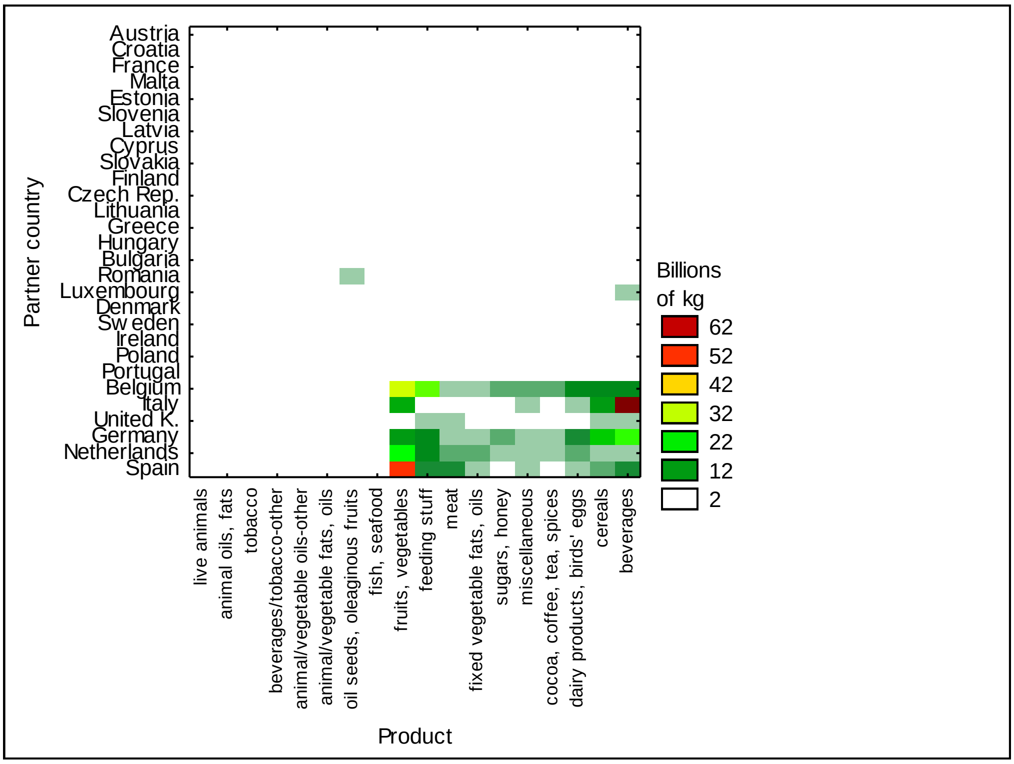

(b) product

Figure S11. Import of food to France in 1999-2018 (billions of kg)

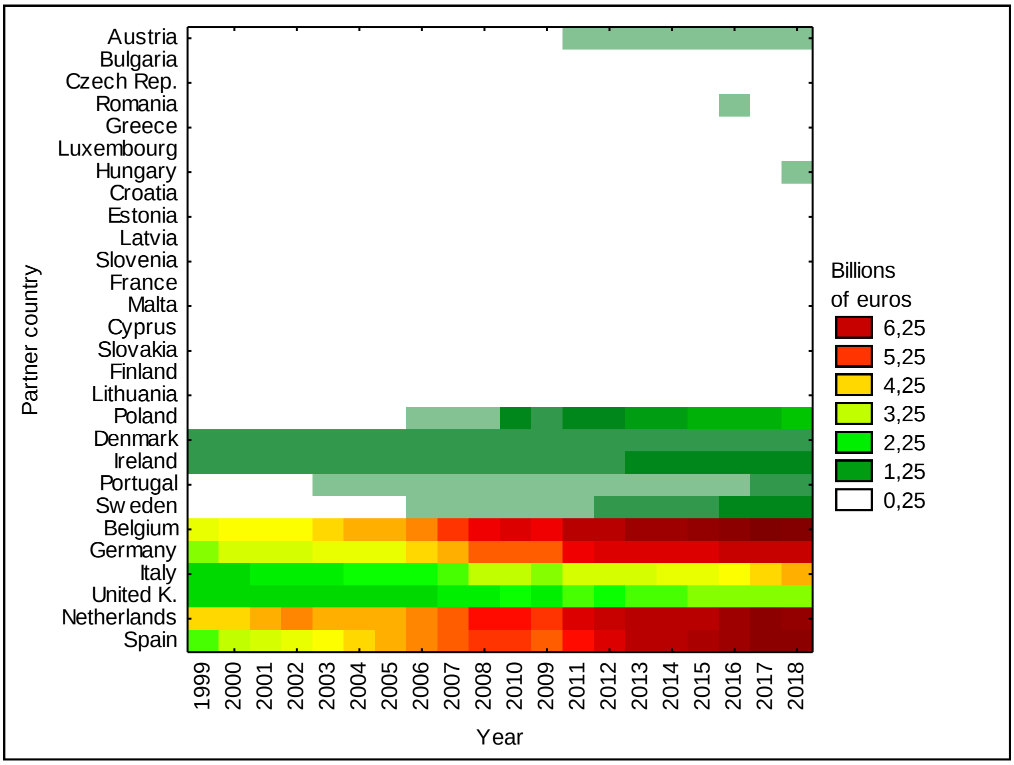

(a) year

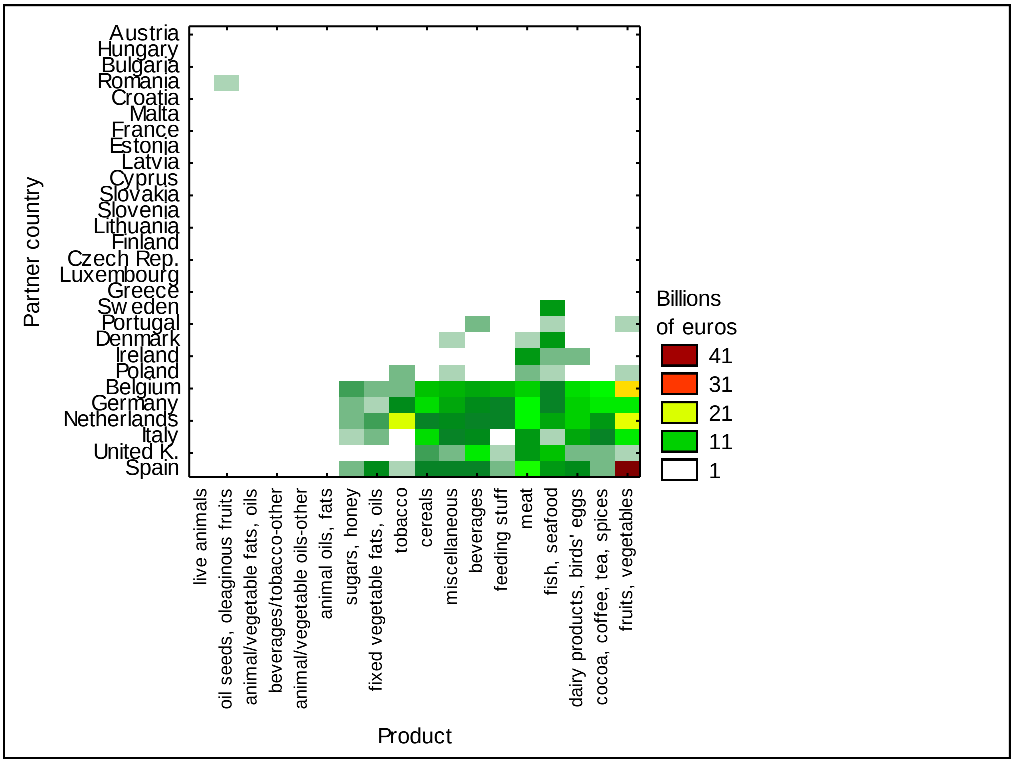

(b) product

Figure S12. Import of food to France in 1999-2018 (billions of euros)

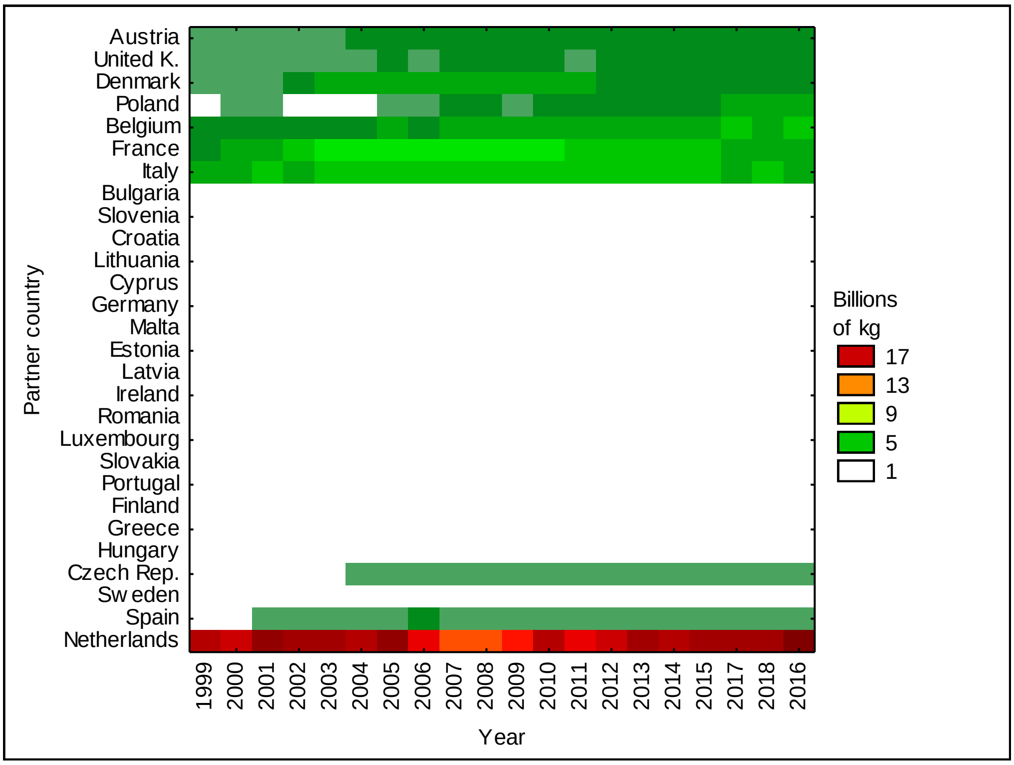

(a) year

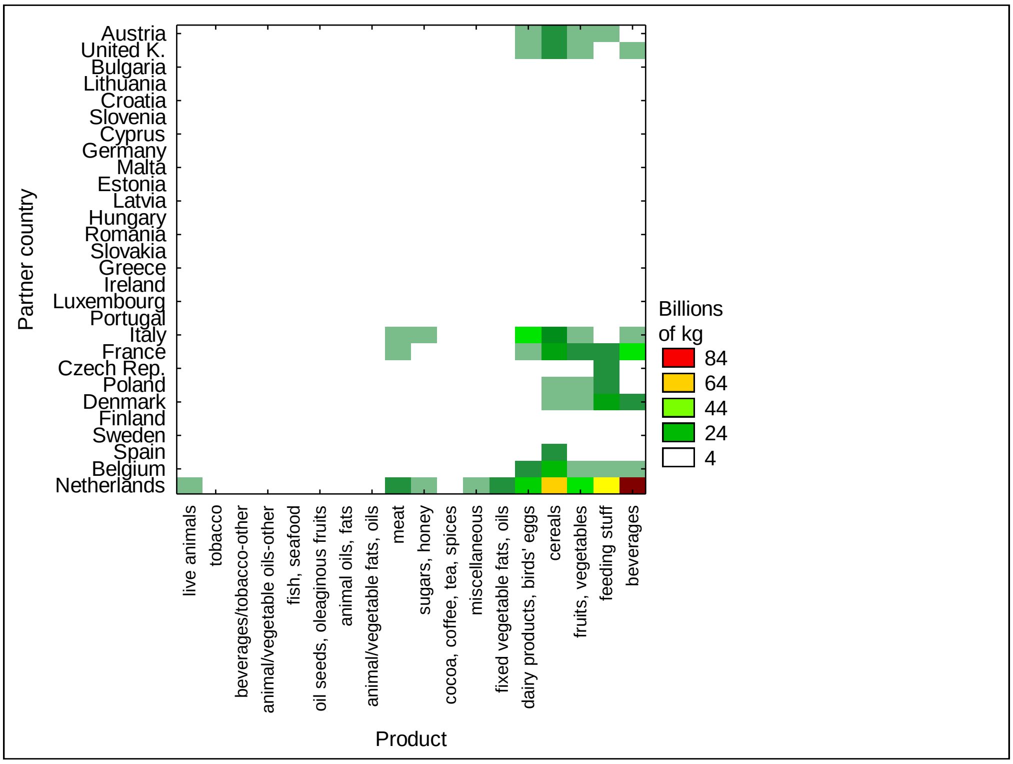

(b) product

Figure S13. Export of food from Germany in 1999-2018 (billions of kg)

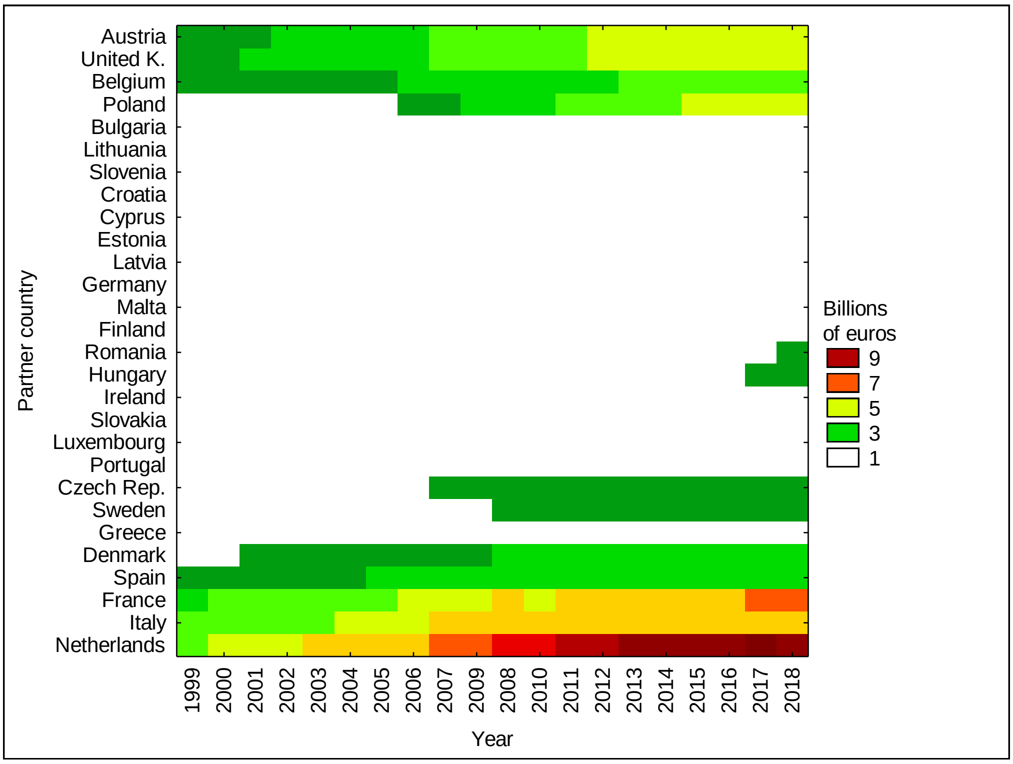

(a) year

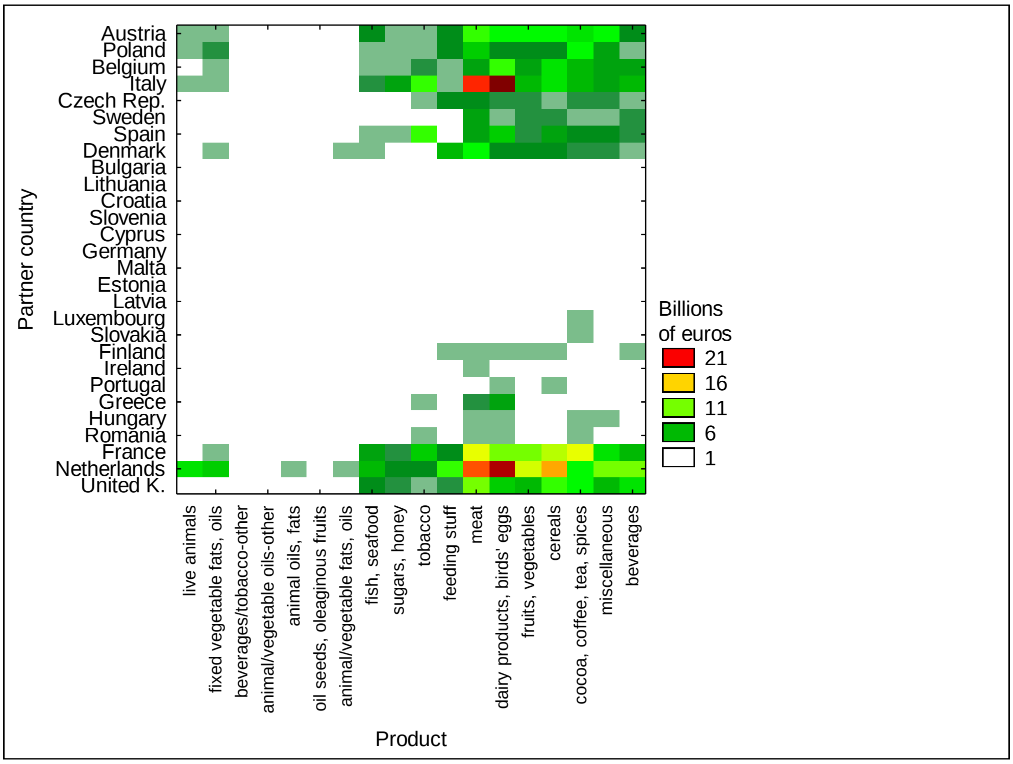

(b) product

Figure S14. Export of food from Germany in 1999-2018 (billions of euros)

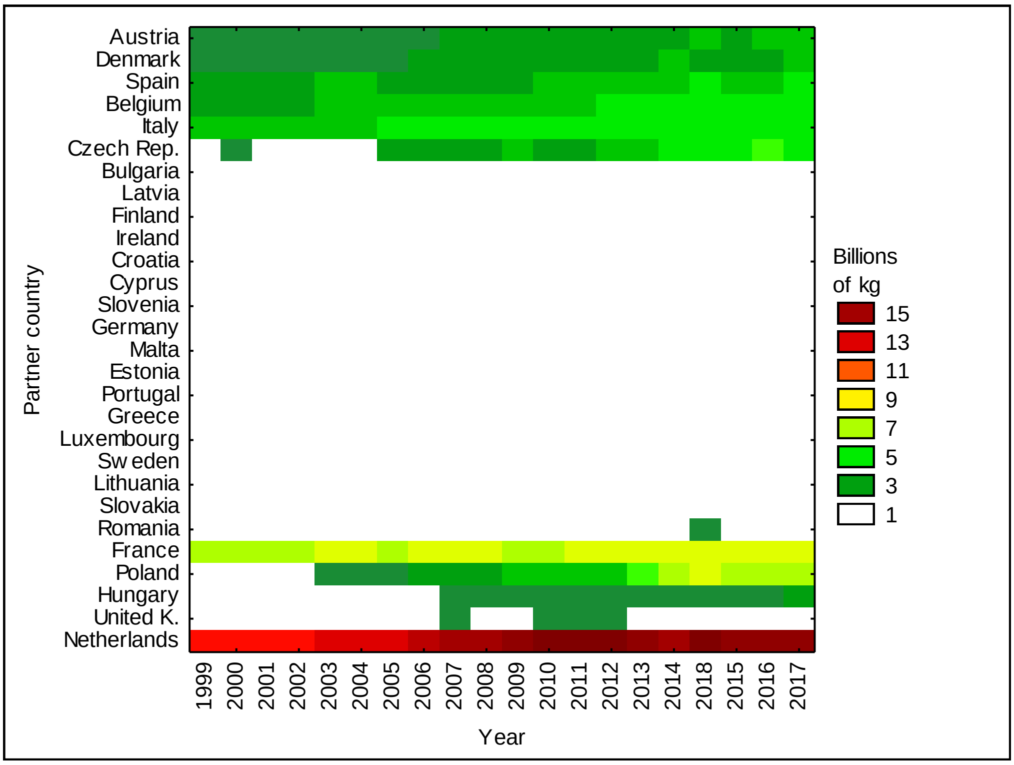

(a) year

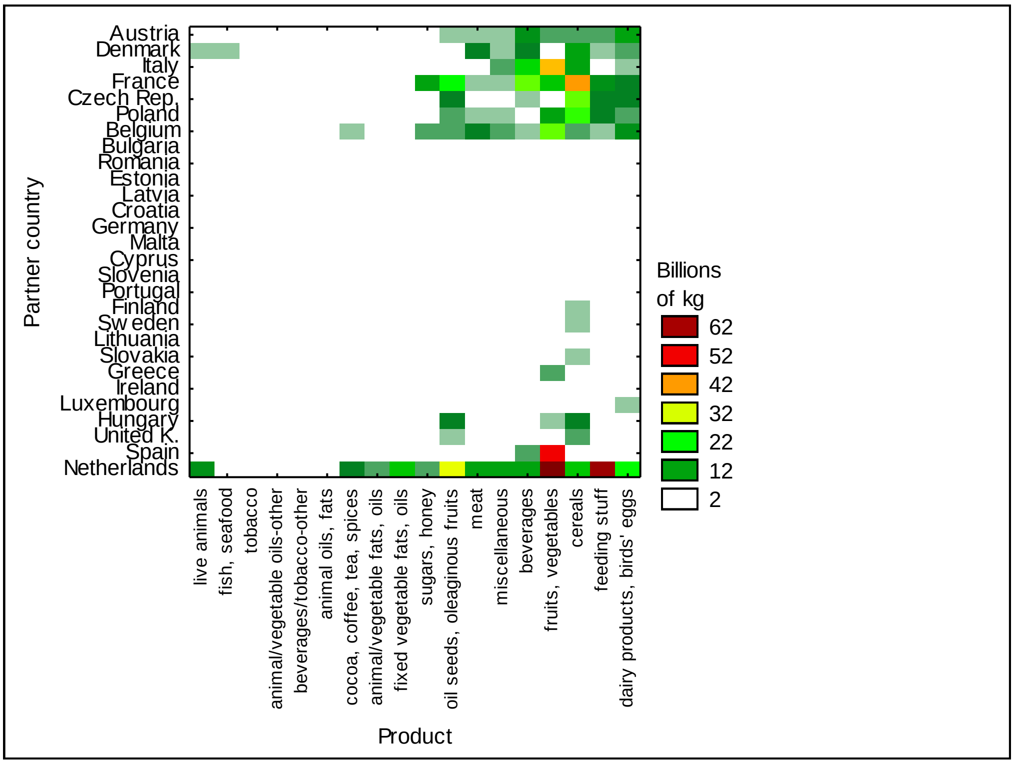

(b) product

Figure S15. Import of food to Germany in 1999-2018 (billions of kg)

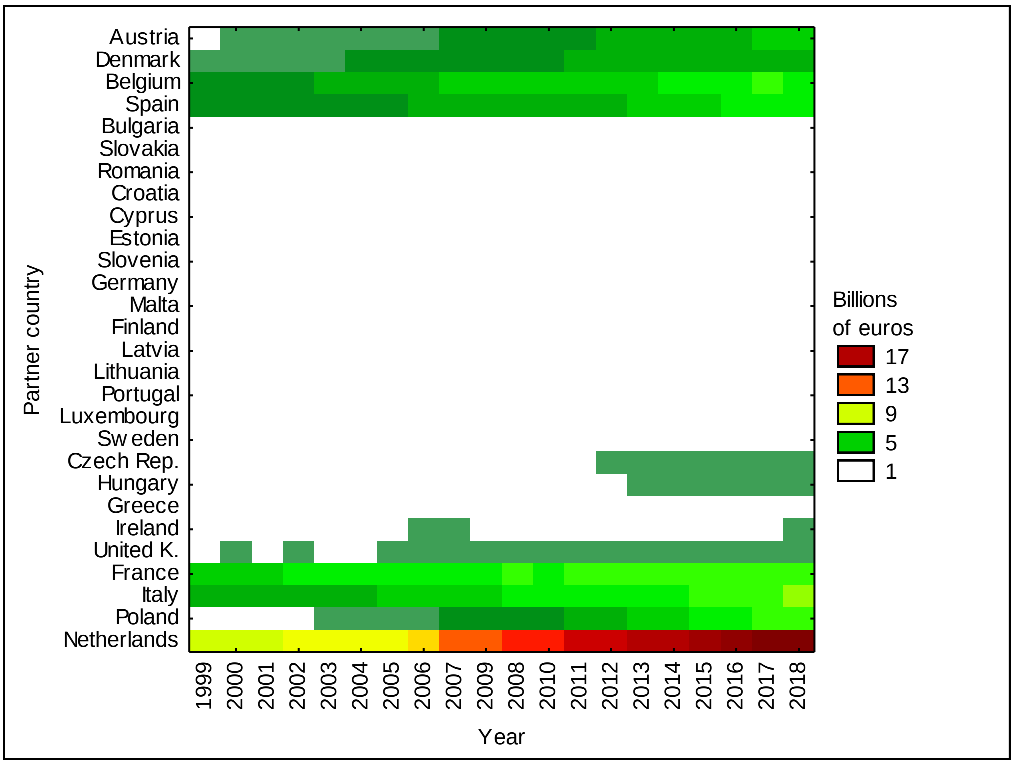

(a) year

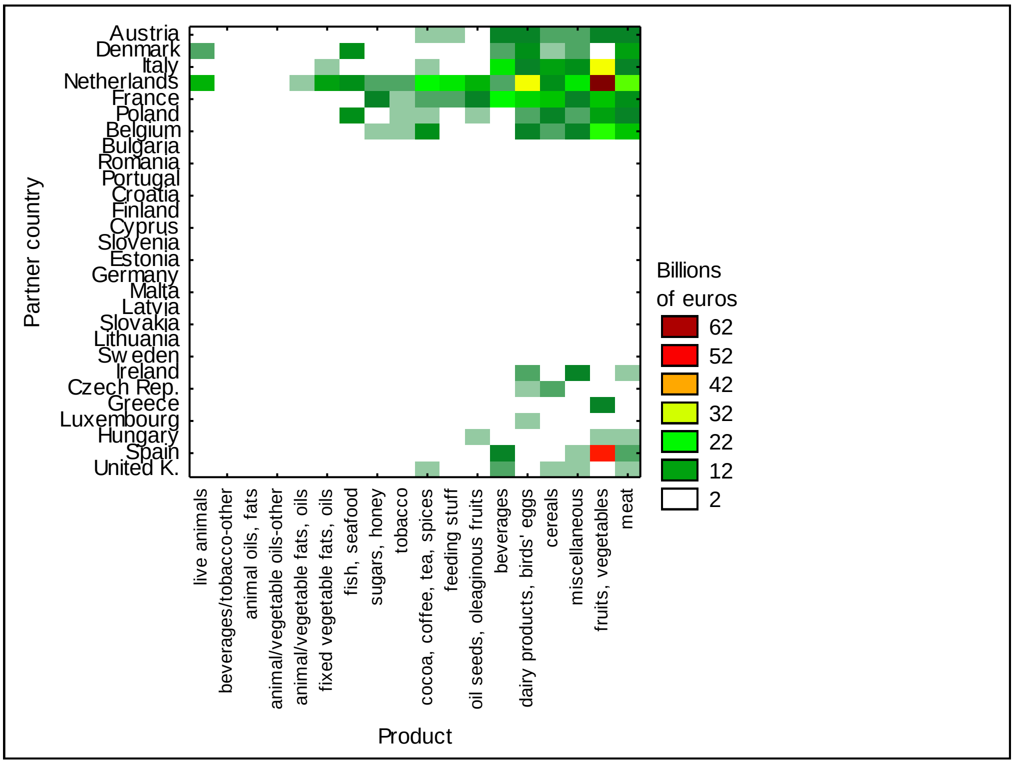

(b) product

Figure S16. Import of food to Germany in 1999-2018 (billions of euros)

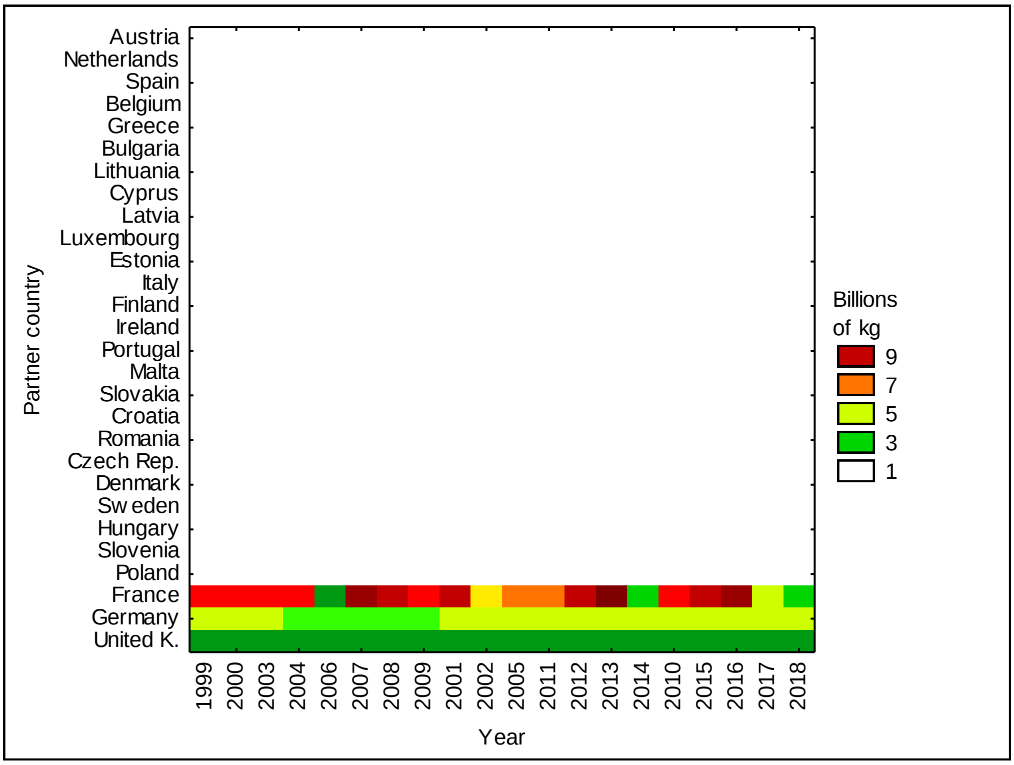

(a) year

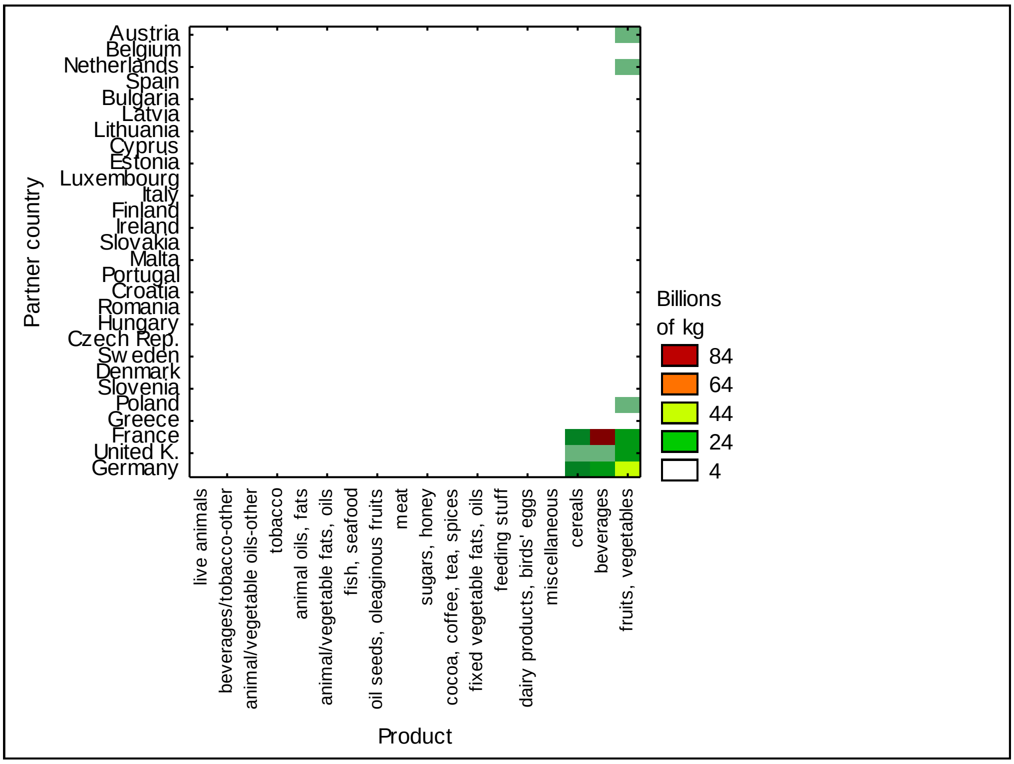

(b) product

Figure S17. Export of food from Italy in 1999-2018 (billions of kg)

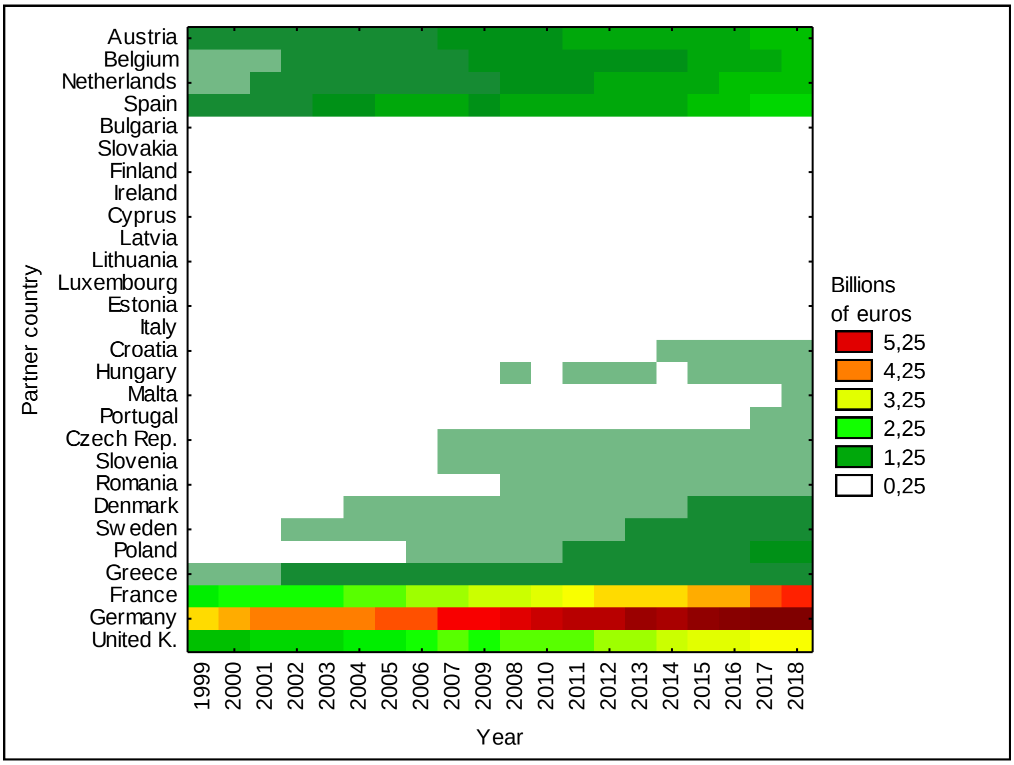

(a) year

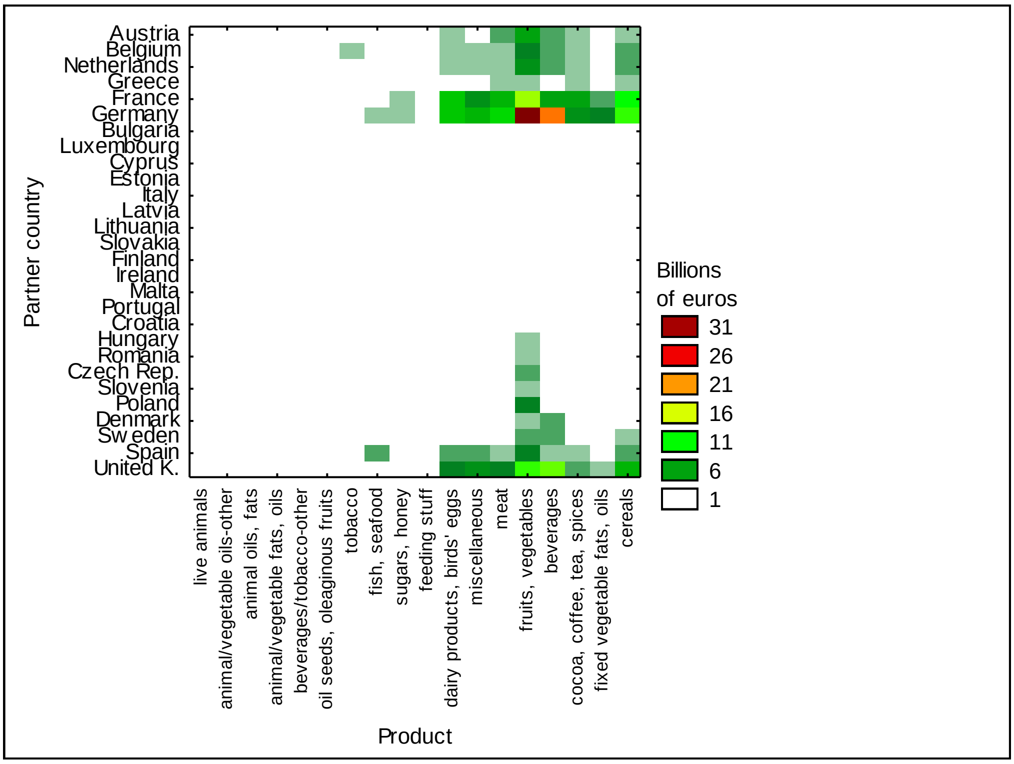

(b) product

Figure S18. Export of food from Italy in 1999-2018 (billions of euros)

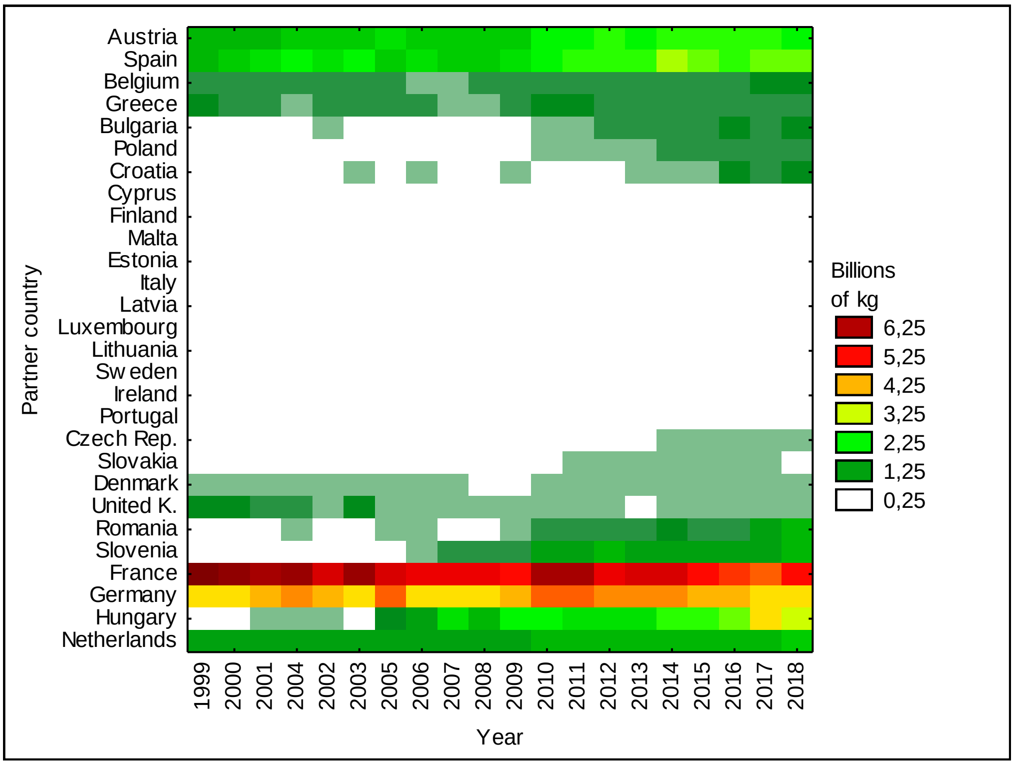

(a) year

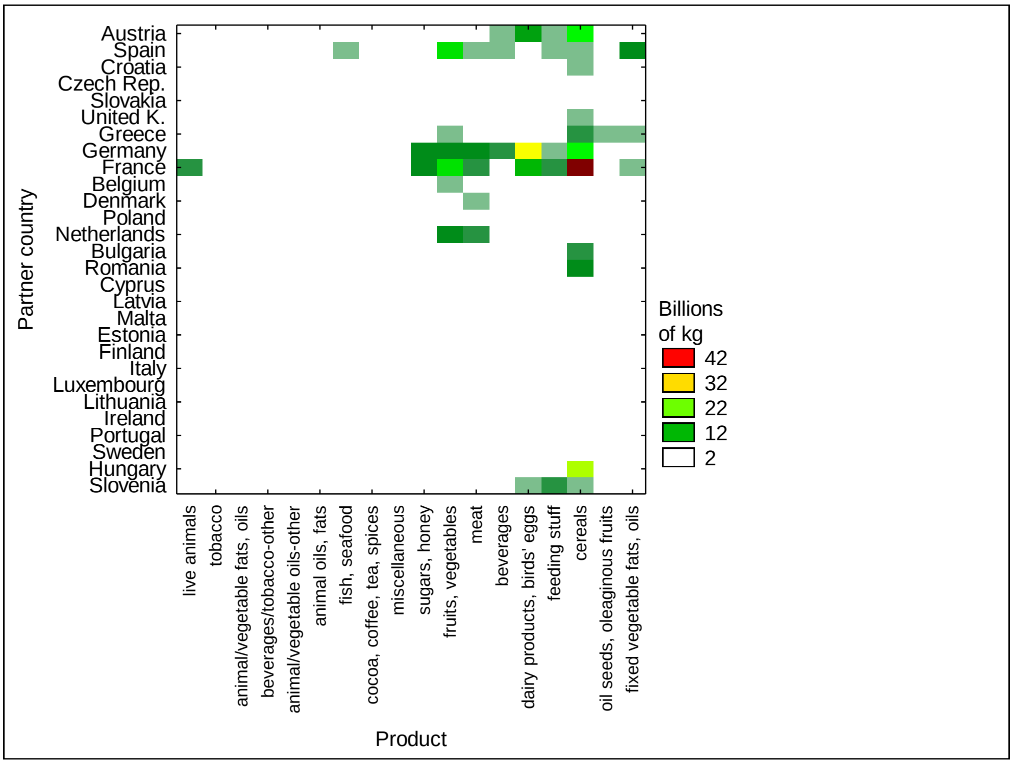

(b) product

Figure S19. Import of food to Italy in 1999-2018 (billions of kg)

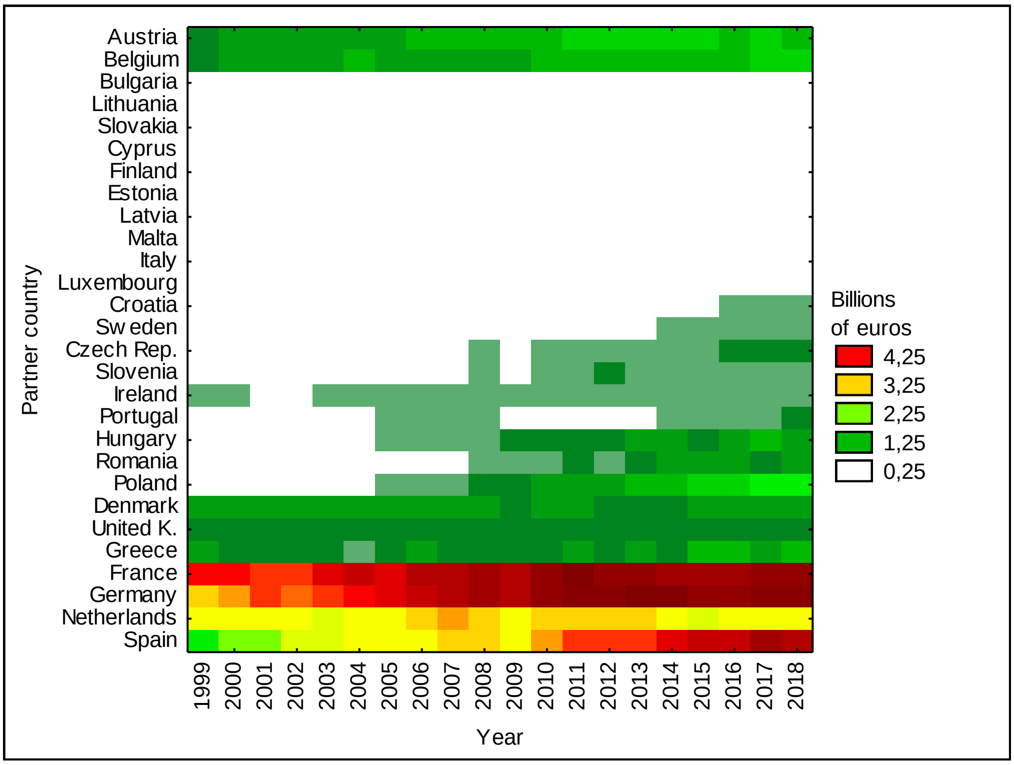

(a) year

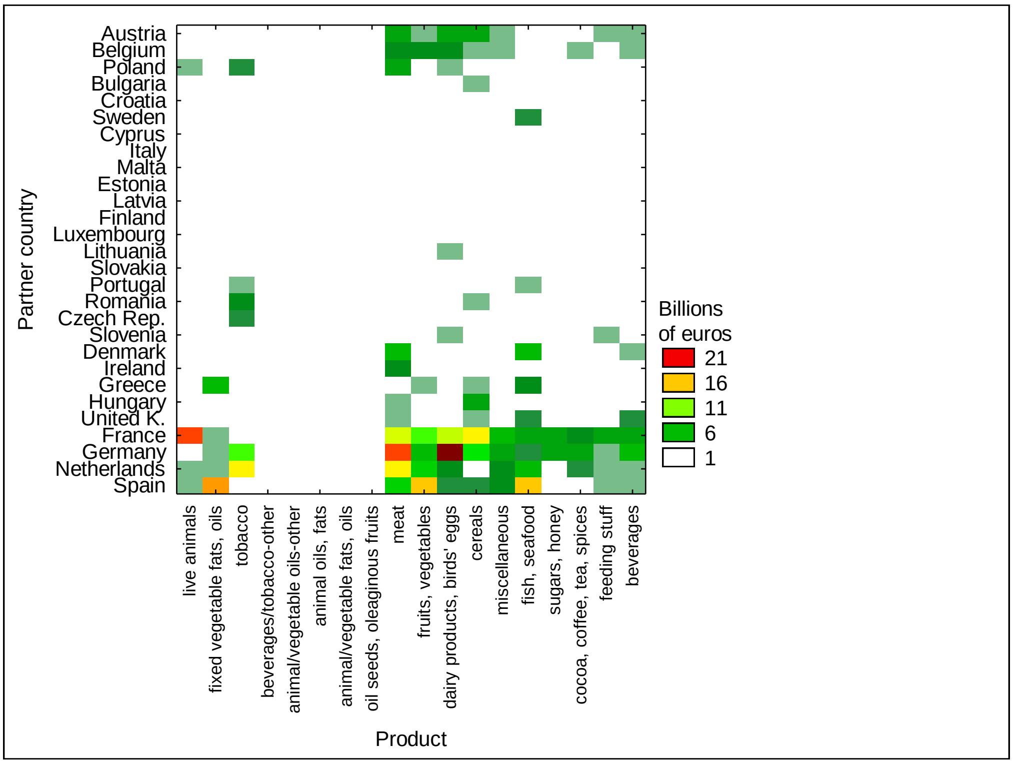

(b) product

Figure S20. Import of food to Italy in 1999-2018 (billions of euros)

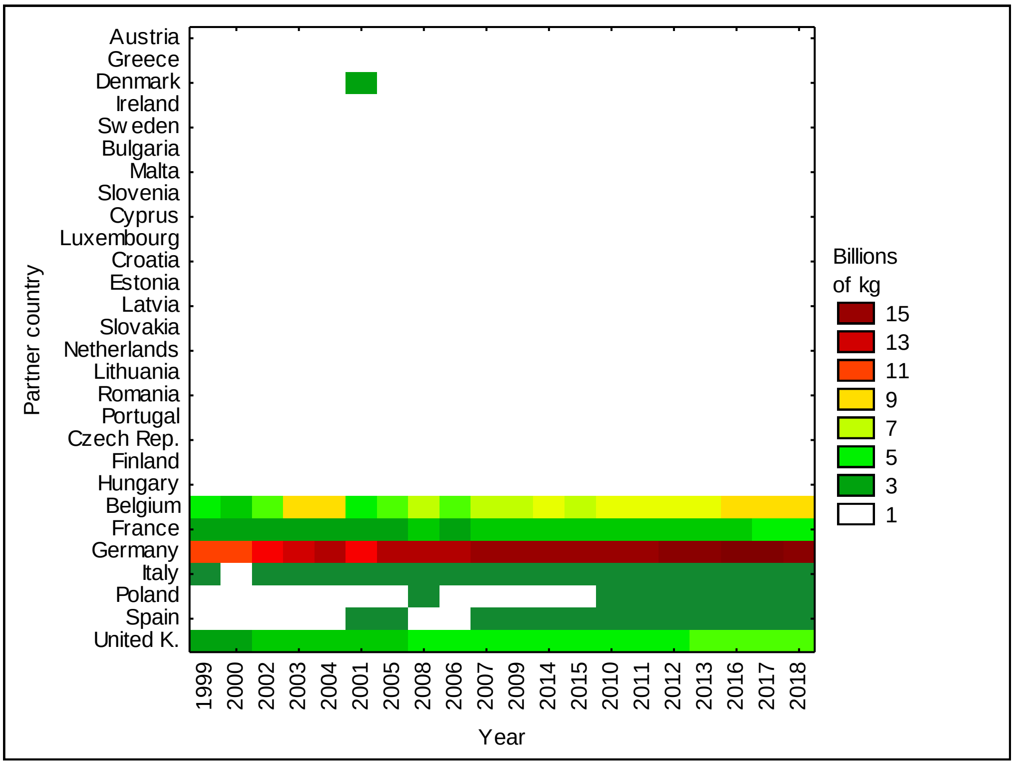

(a) year

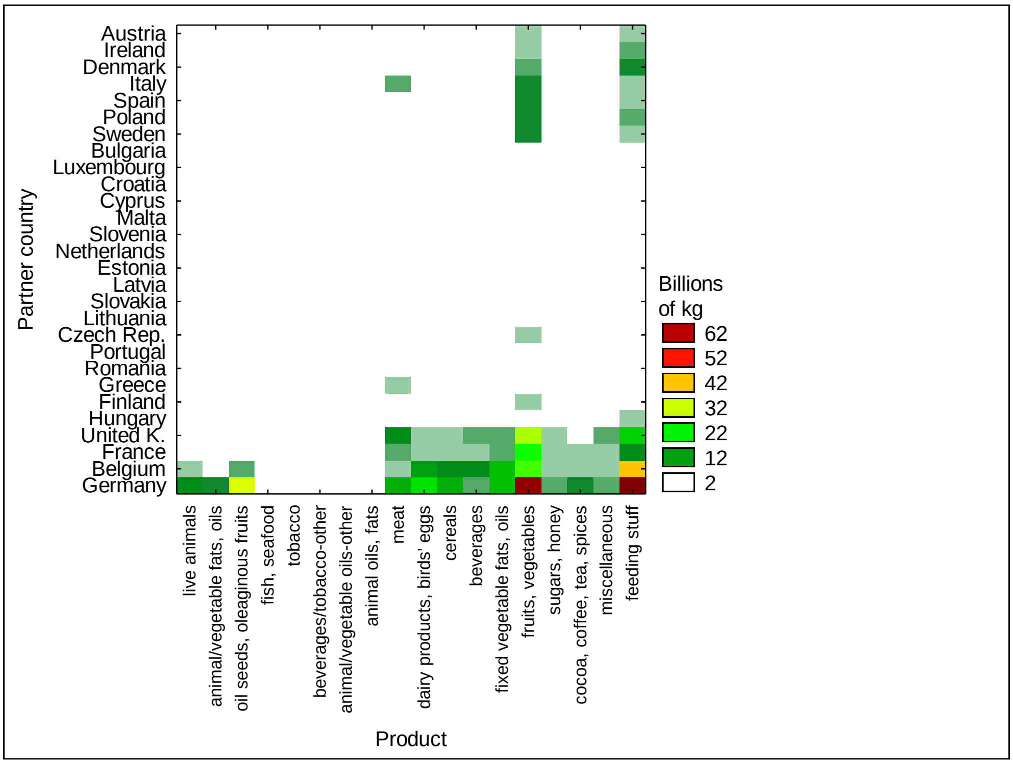

(b) product

Figure S21. Export of food from Netherlands in 1999-2018 (billions of kg)

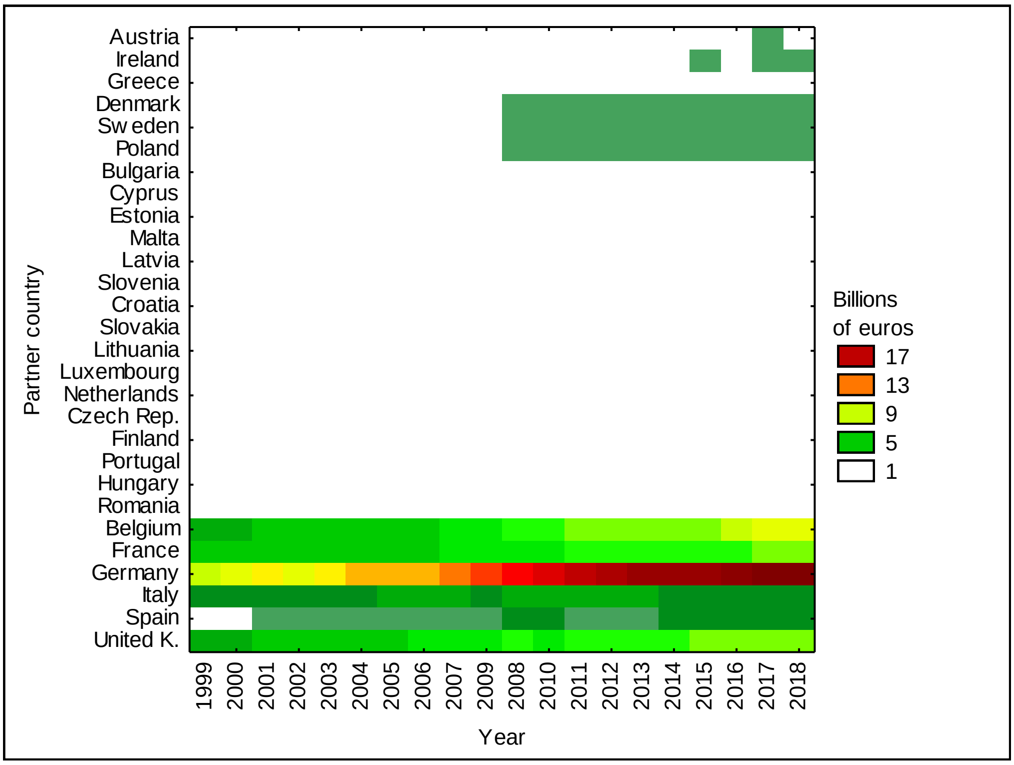

(a) year

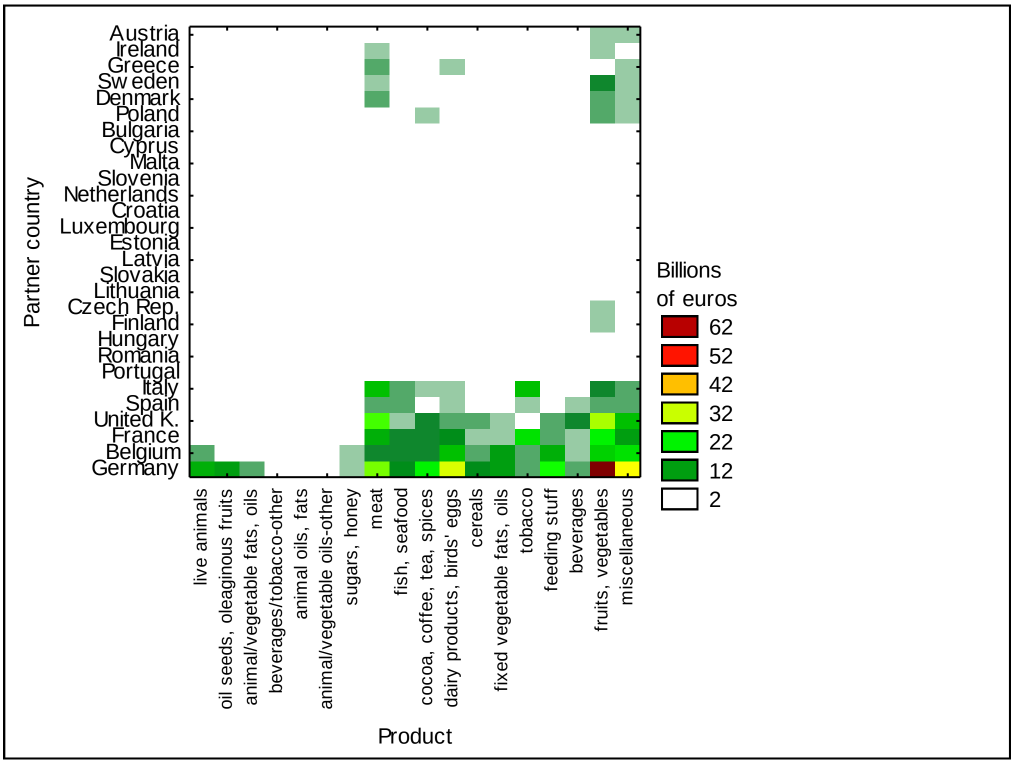

(b) product

Figure S22. Export of food from Netherlands in 1999-2018 (billions of euros)

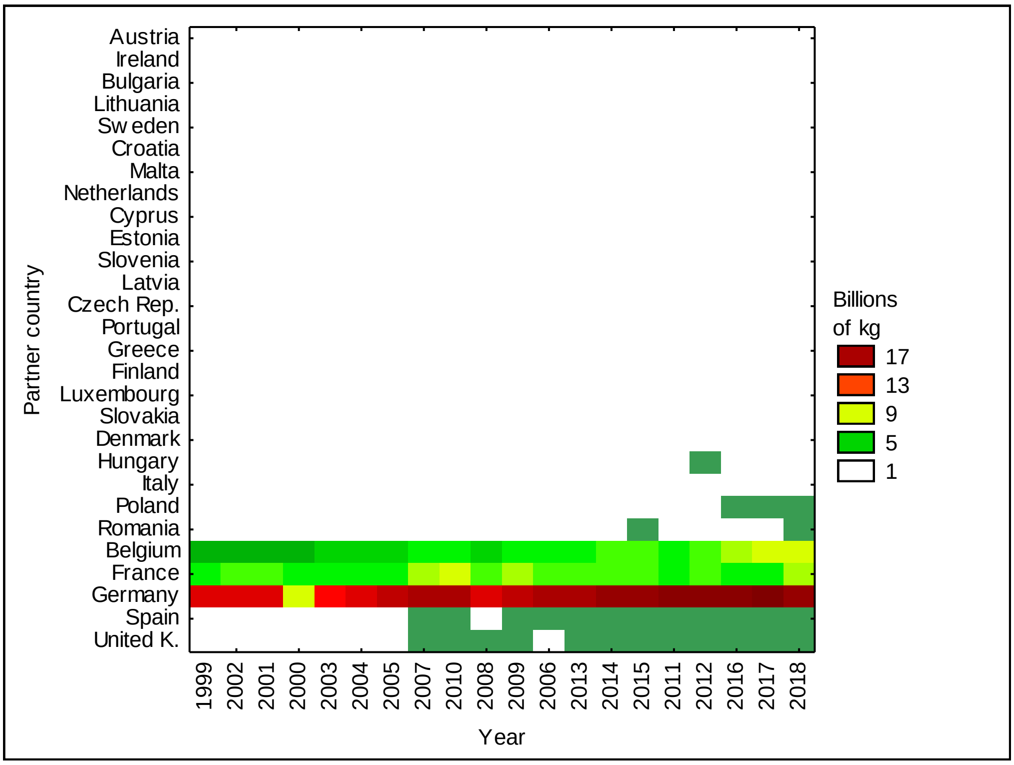

(a) year

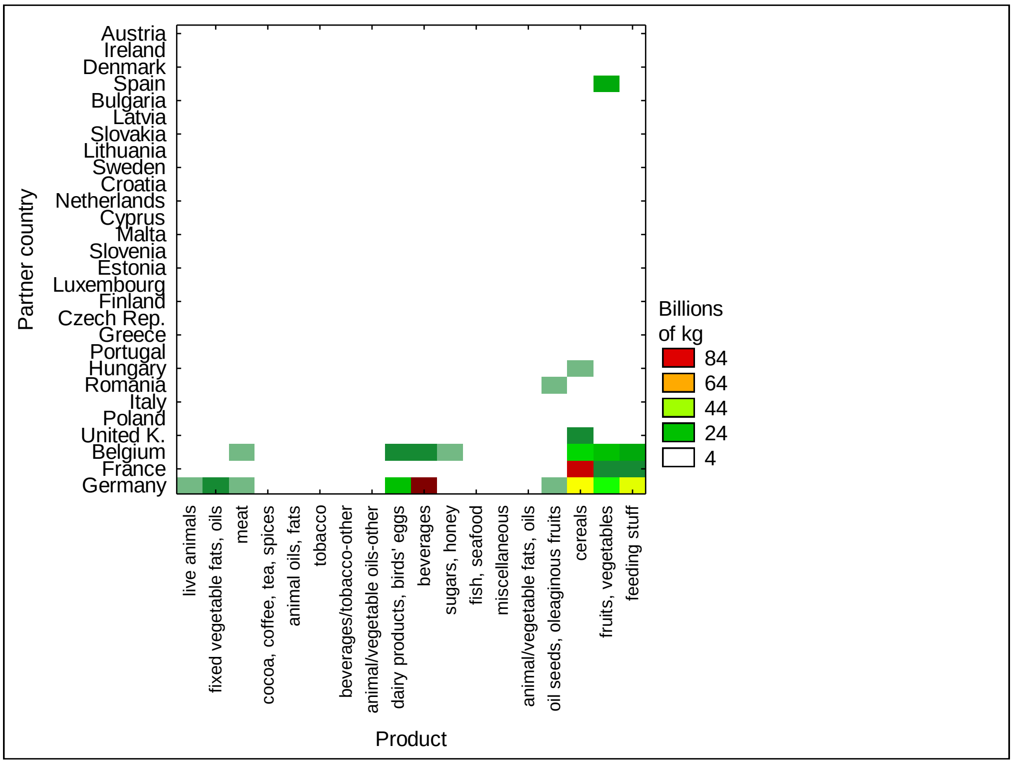

(b) product

Figure S23. Import of food to Netherlands in 1999-2018 (billions of kg)

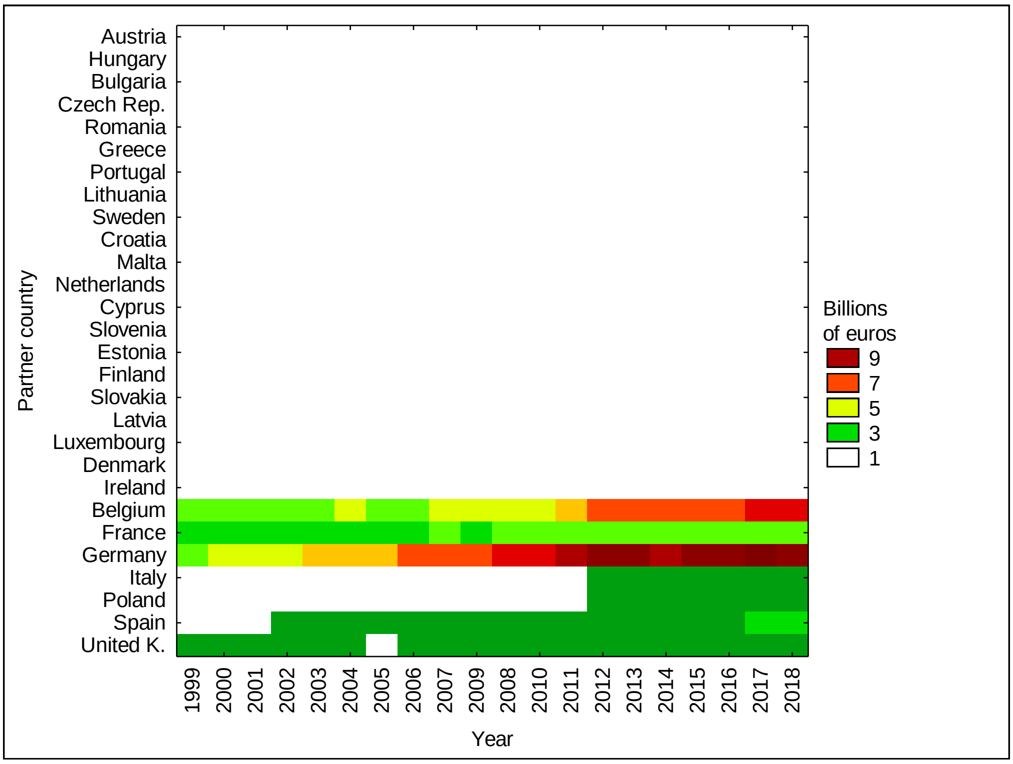

(a) year

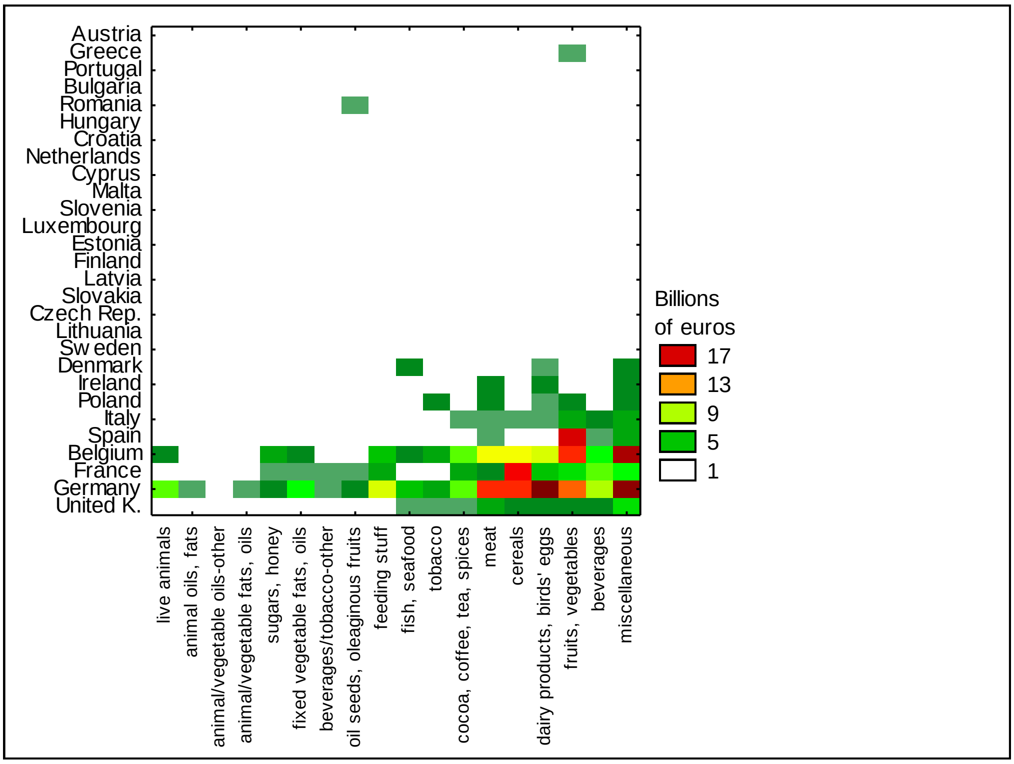

(b) product

Figure S24. Import of food to Netherlands in 1999-2018 (billions of euros)

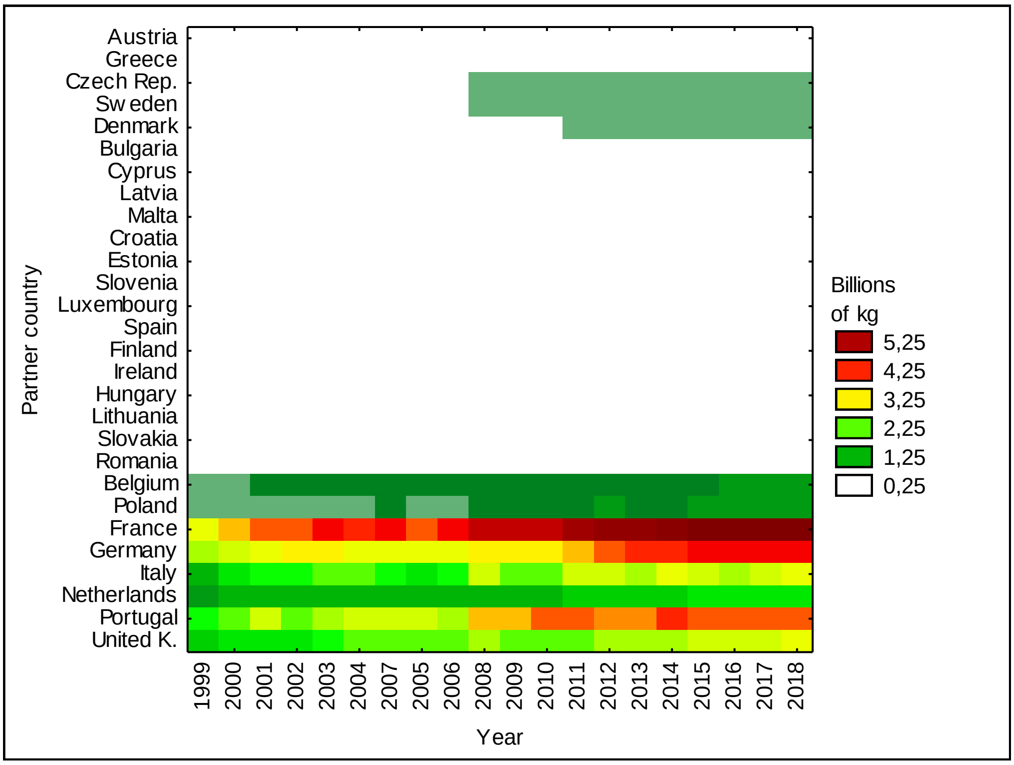

(a) year

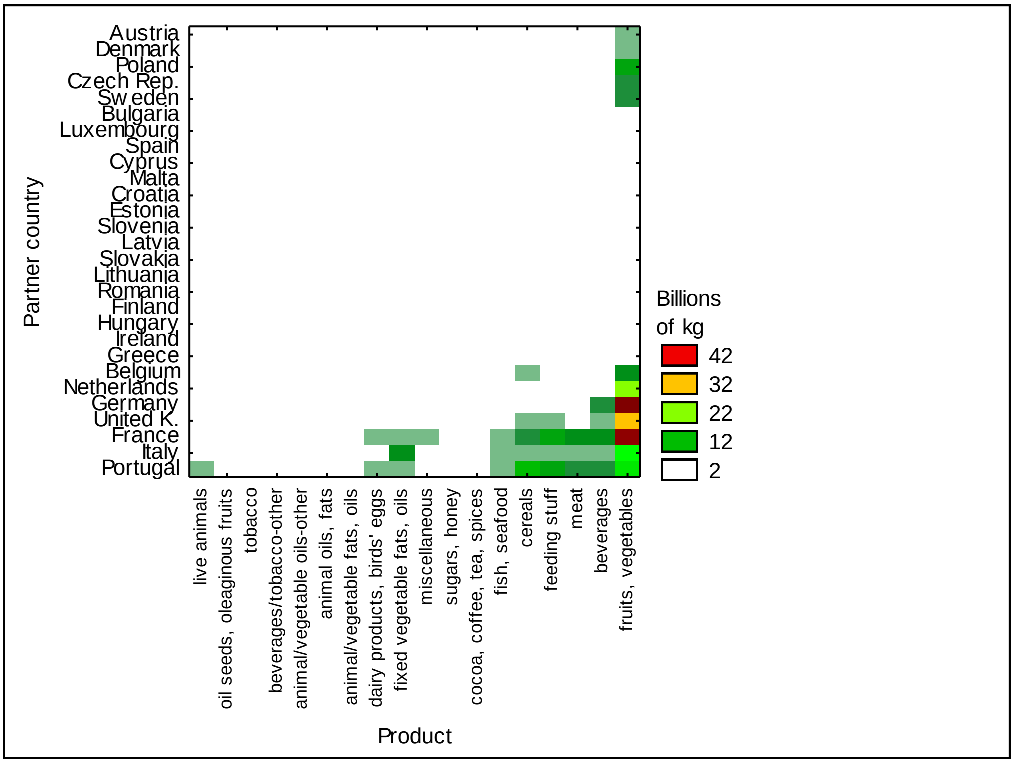

(b) product

Figure S25. Export of food from Spain in 1999-2018 (billions of kg)

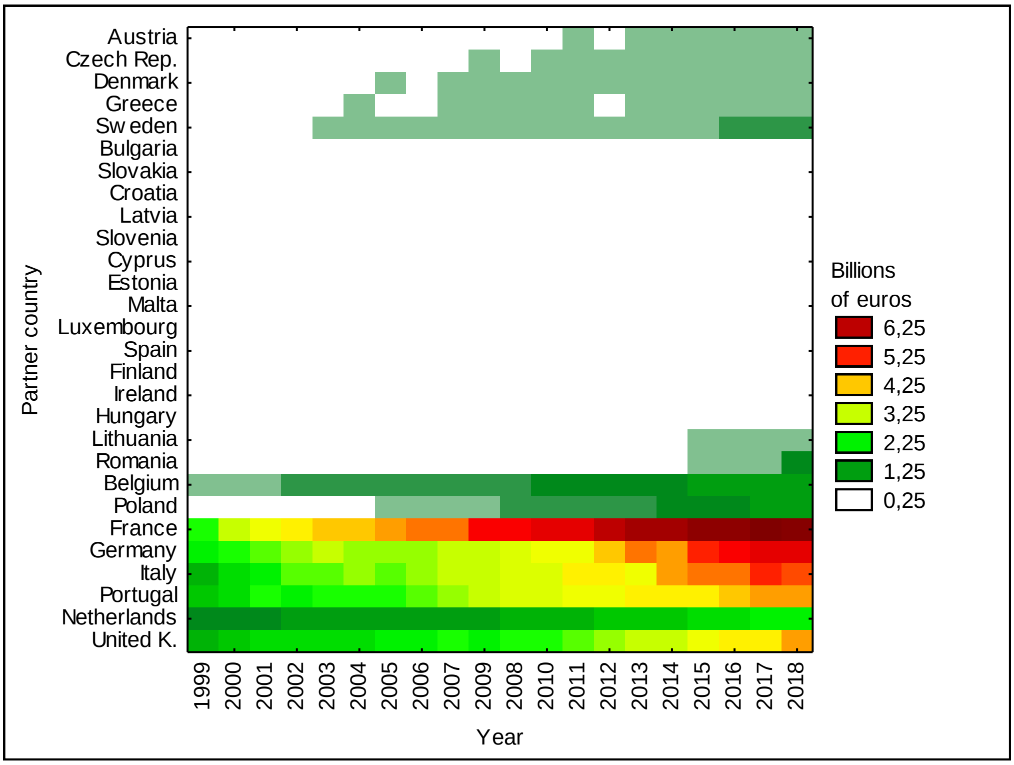

(a) year

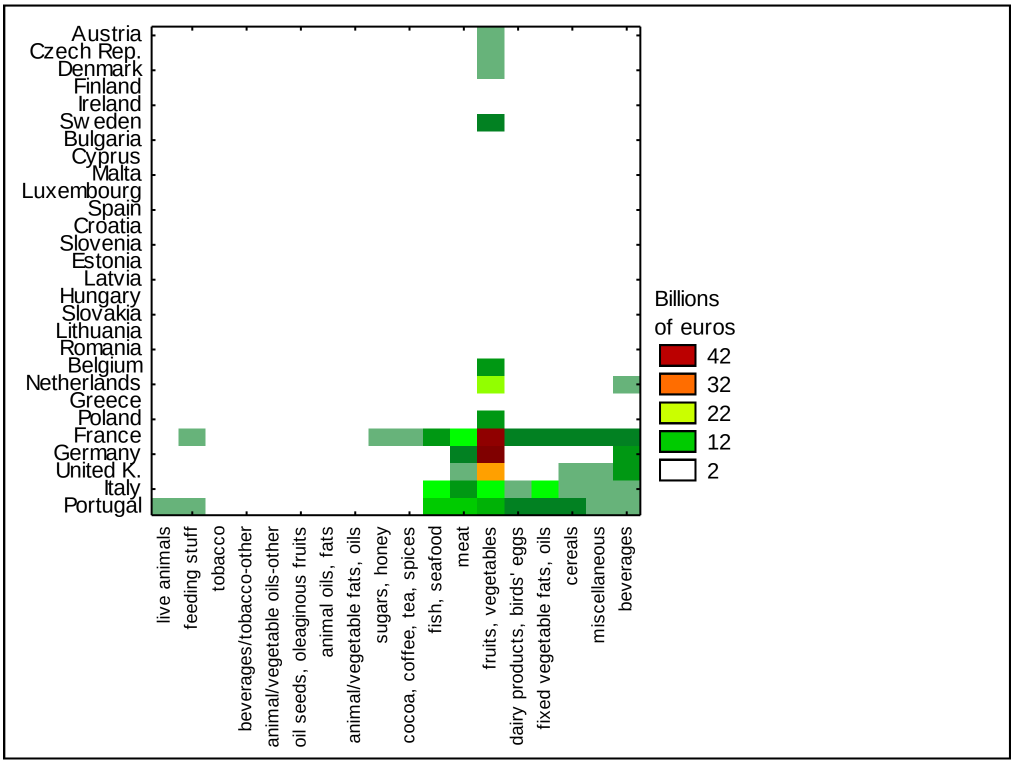

(b) product

Figure S26. Export of food from Spain in 1999-2018 (billions of euros)

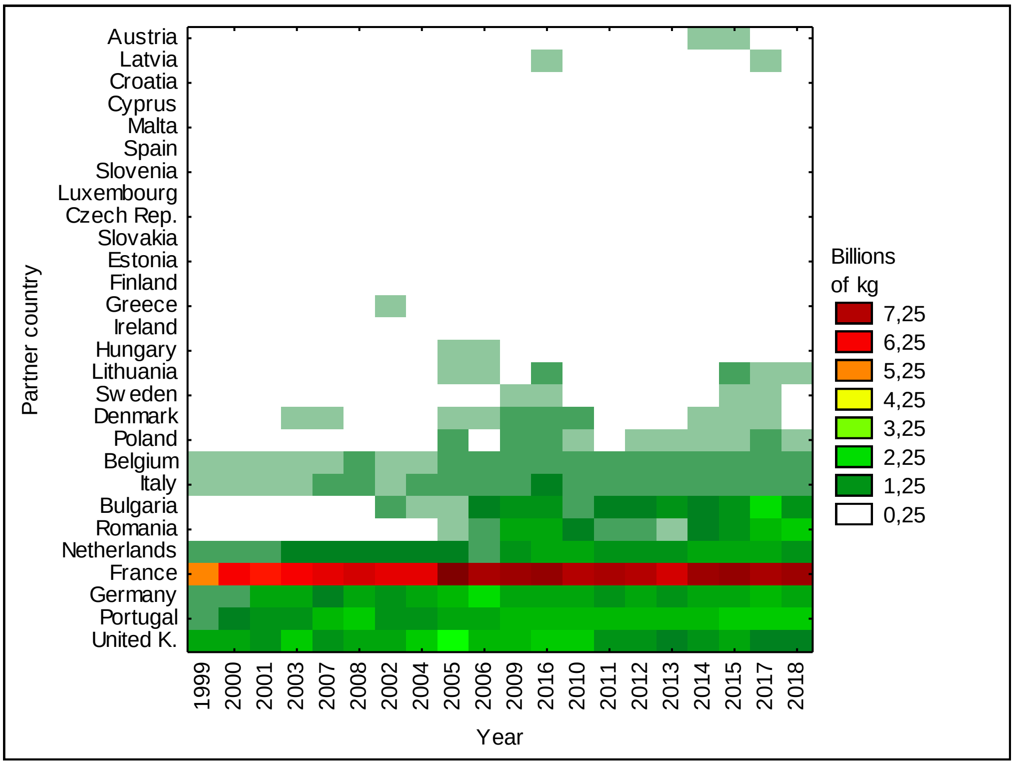

(a) year

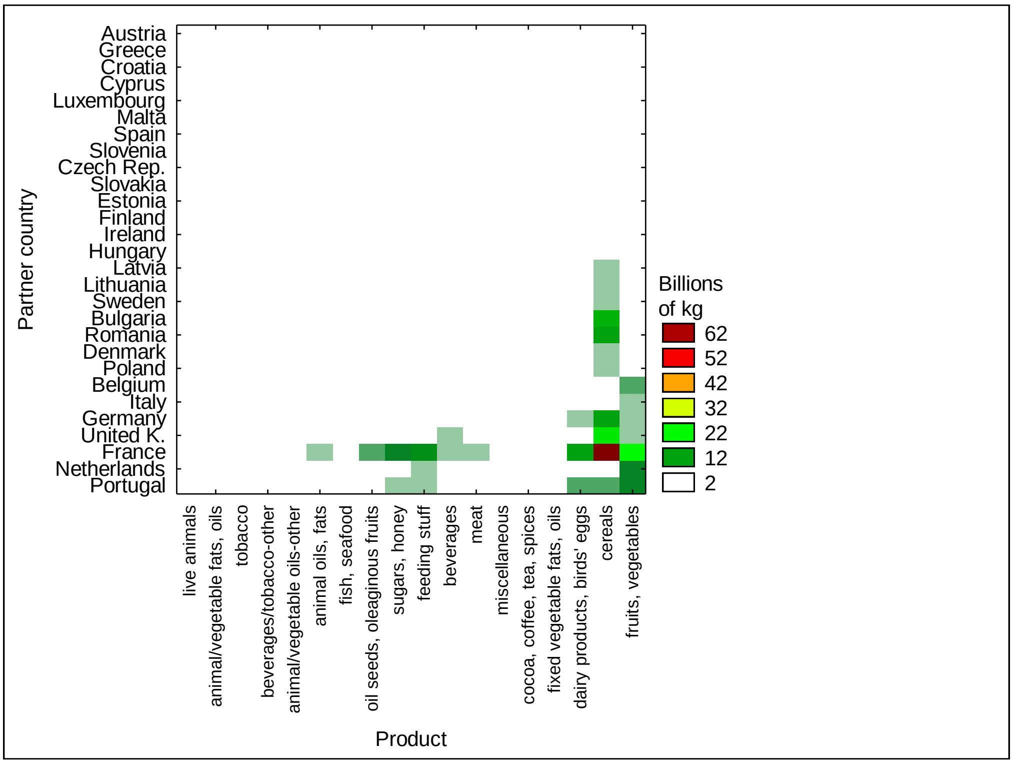

(b) product

Figure S27. Import of food to Spain in 1999-2018 (billions of kg)

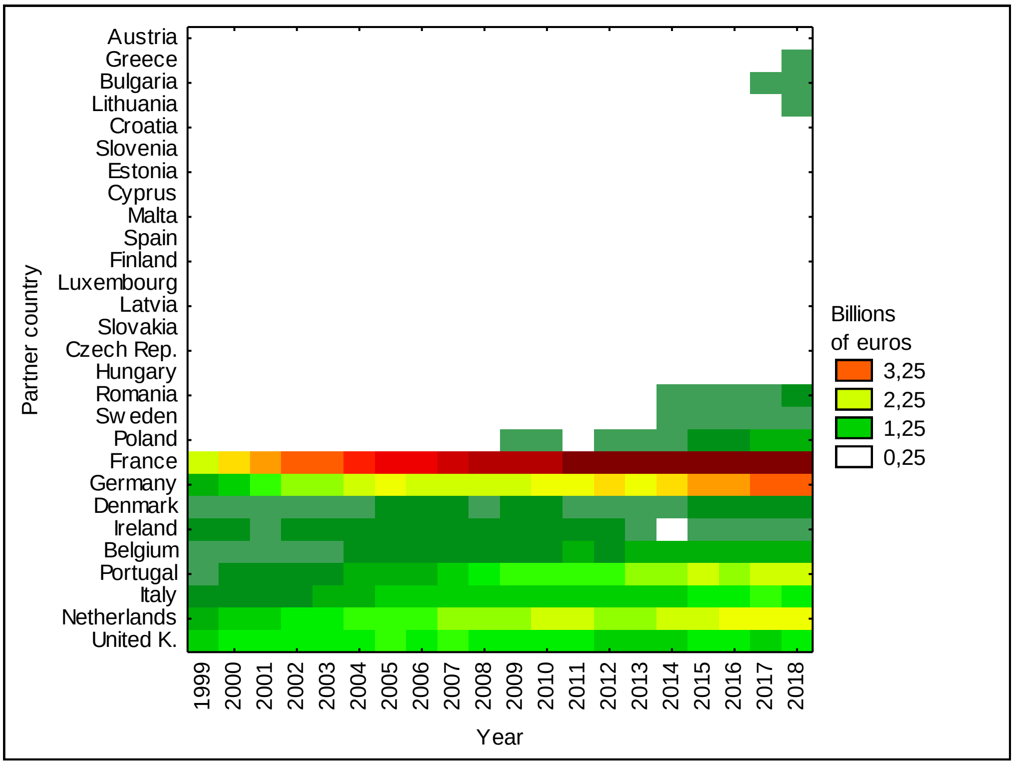

(a) year

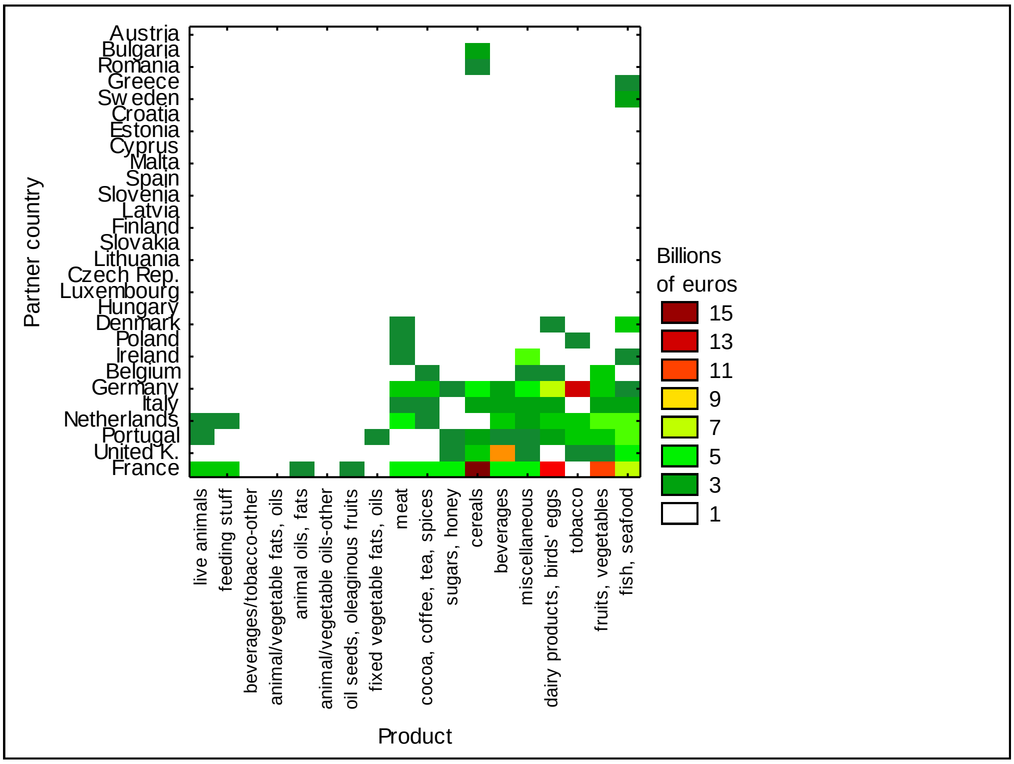

(b) product

Figure S28. Import of food to Spain in 1999-2018 (billions of euros)

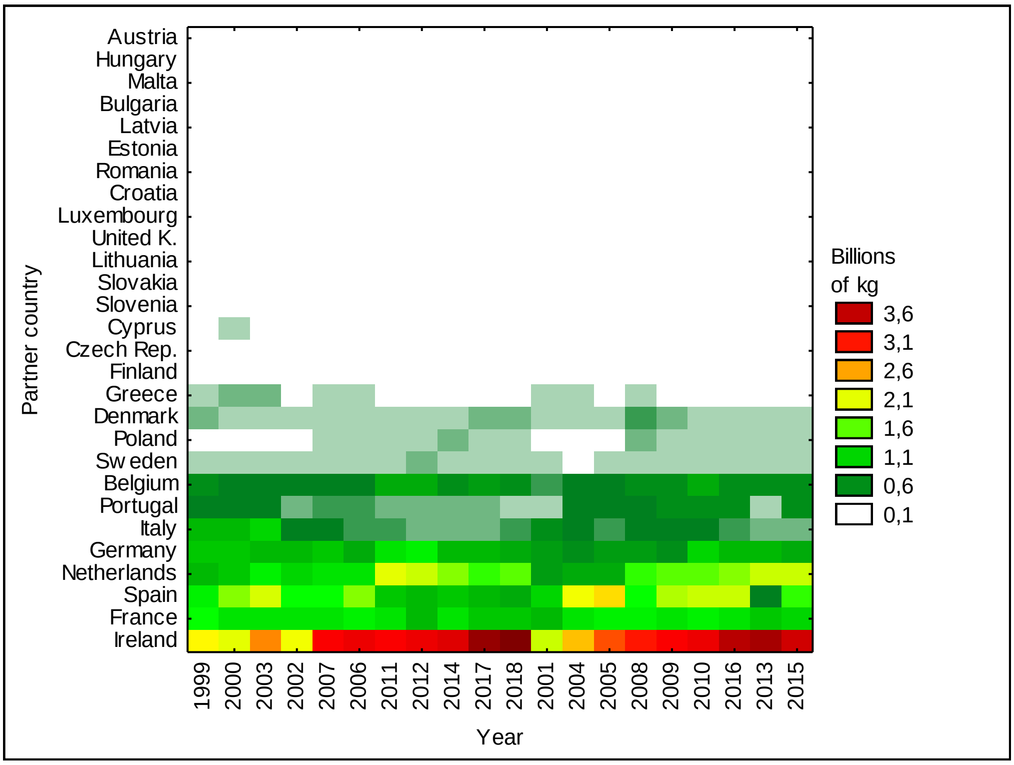

(a) year

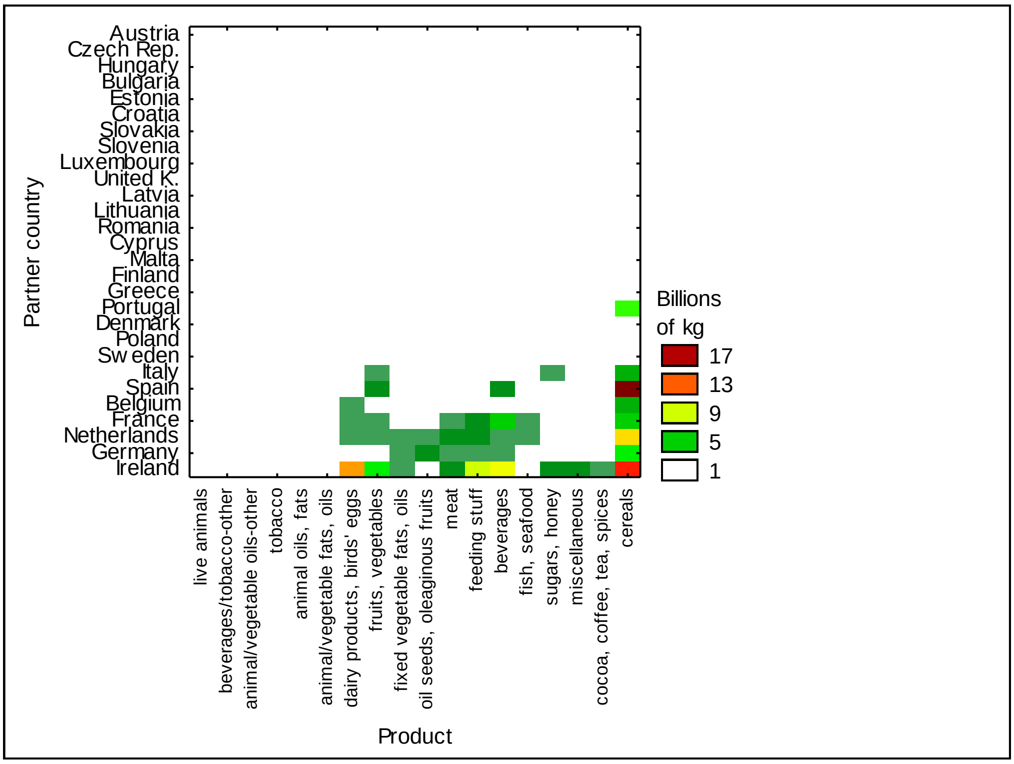

(b) product

Figure S29. Export of food from United Kingdom in 1999-2018 (billions of kg)

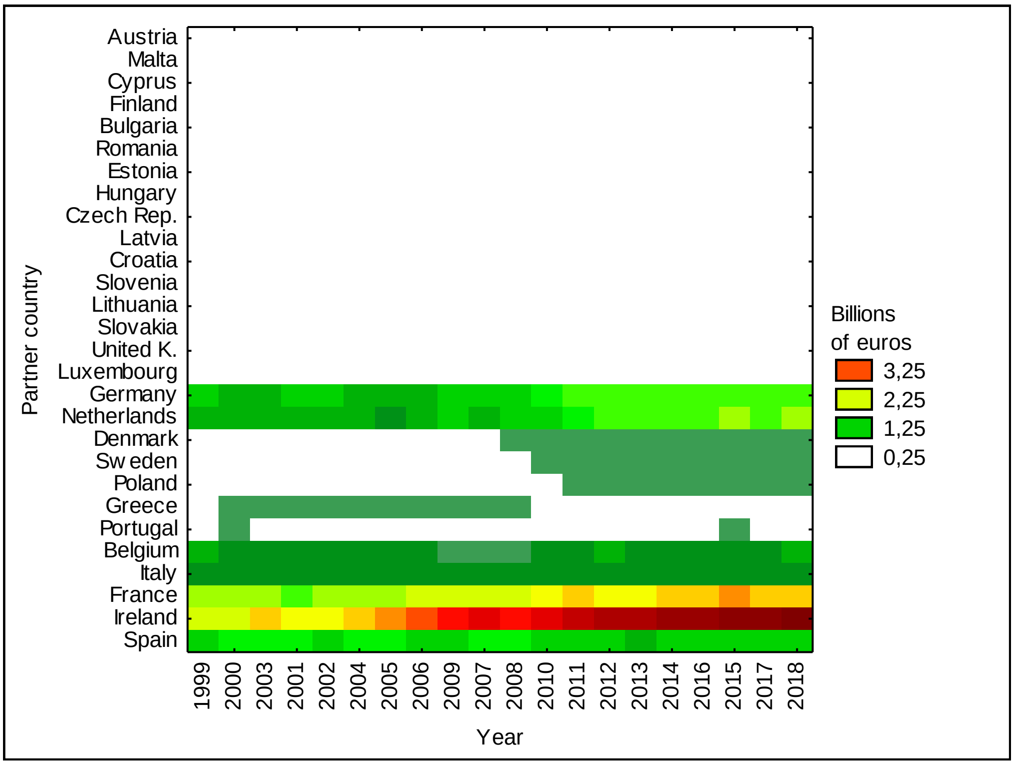

(a) year

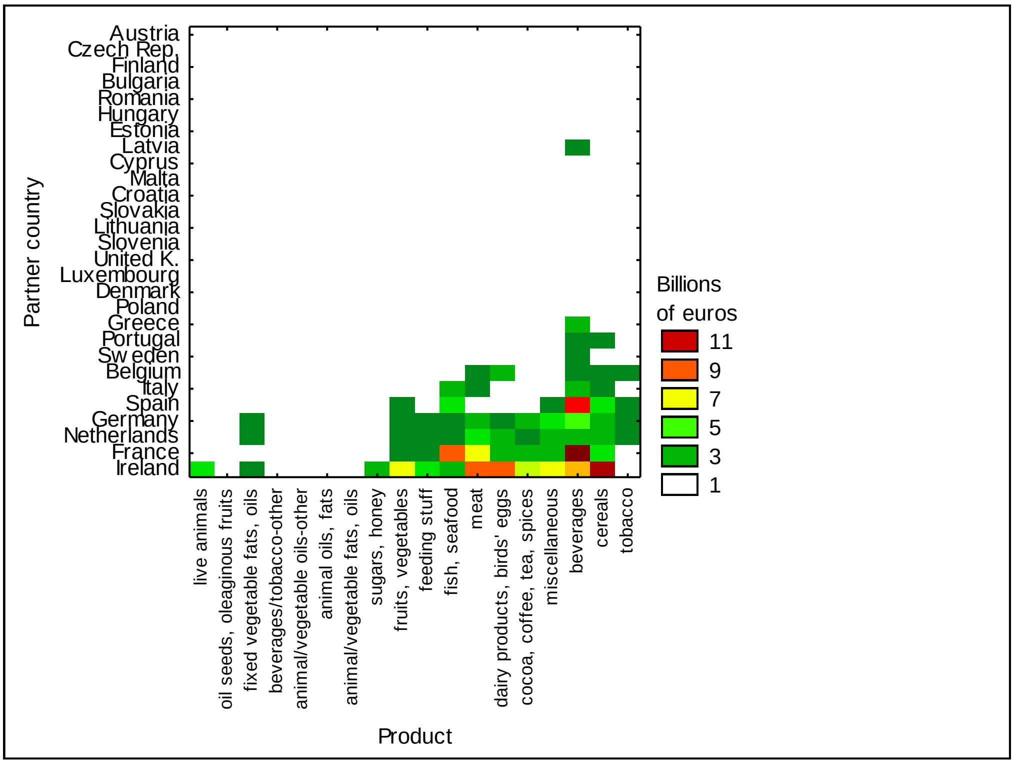

(b) product

Figure S30. Export of food from United Kingdom in 1999-2018 (billions of euros)

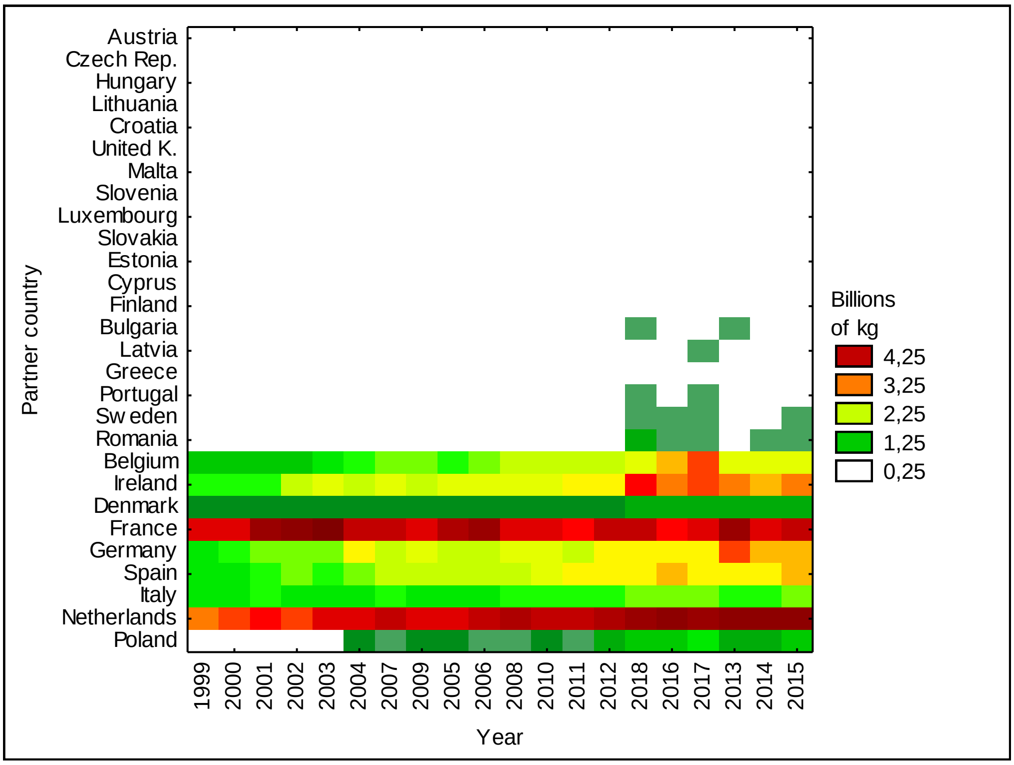

(a) year

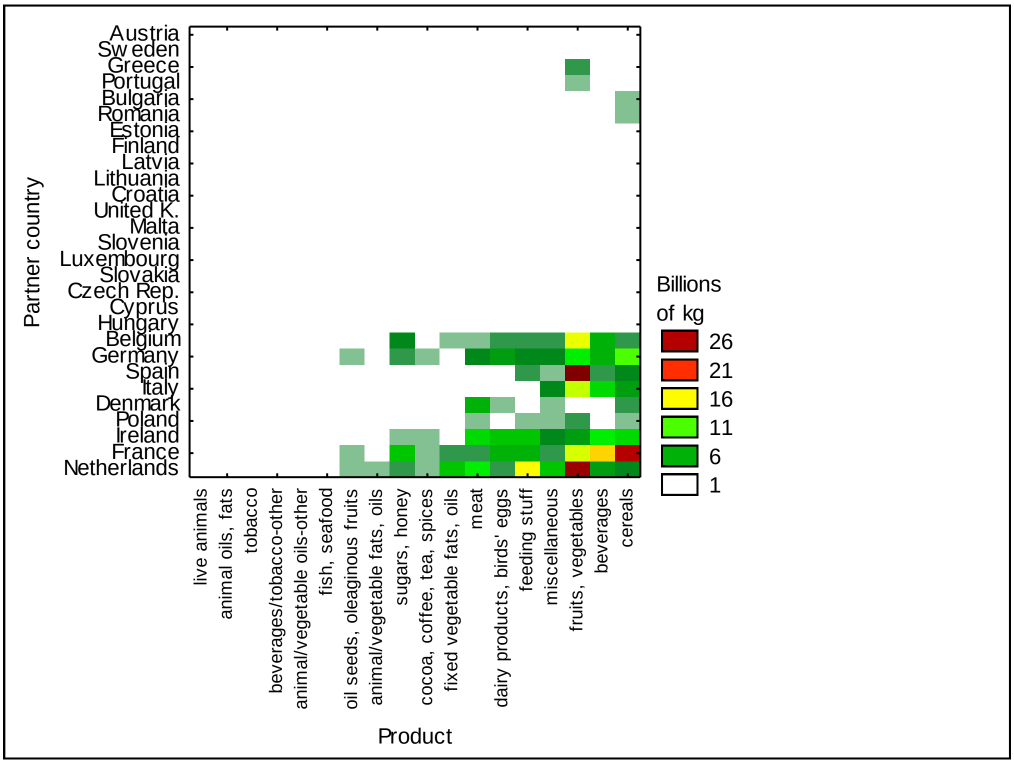

(b) product

Figure S31. Import of food to United Kingdom in 1999-2018 (billions of kg)

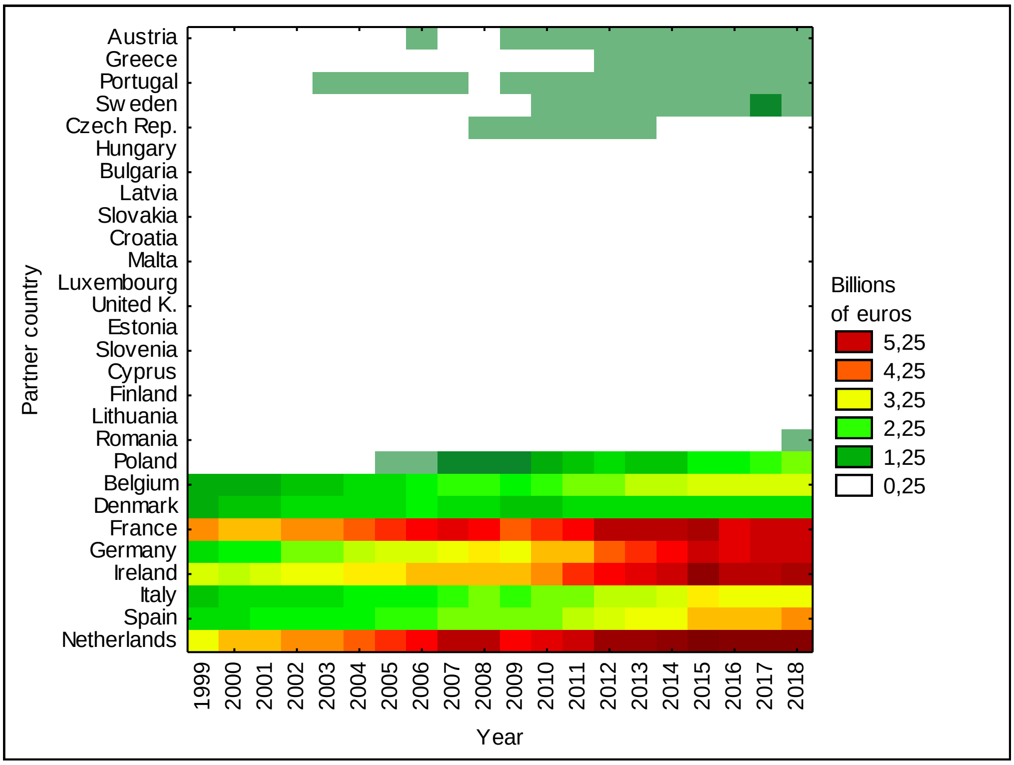

(a) year

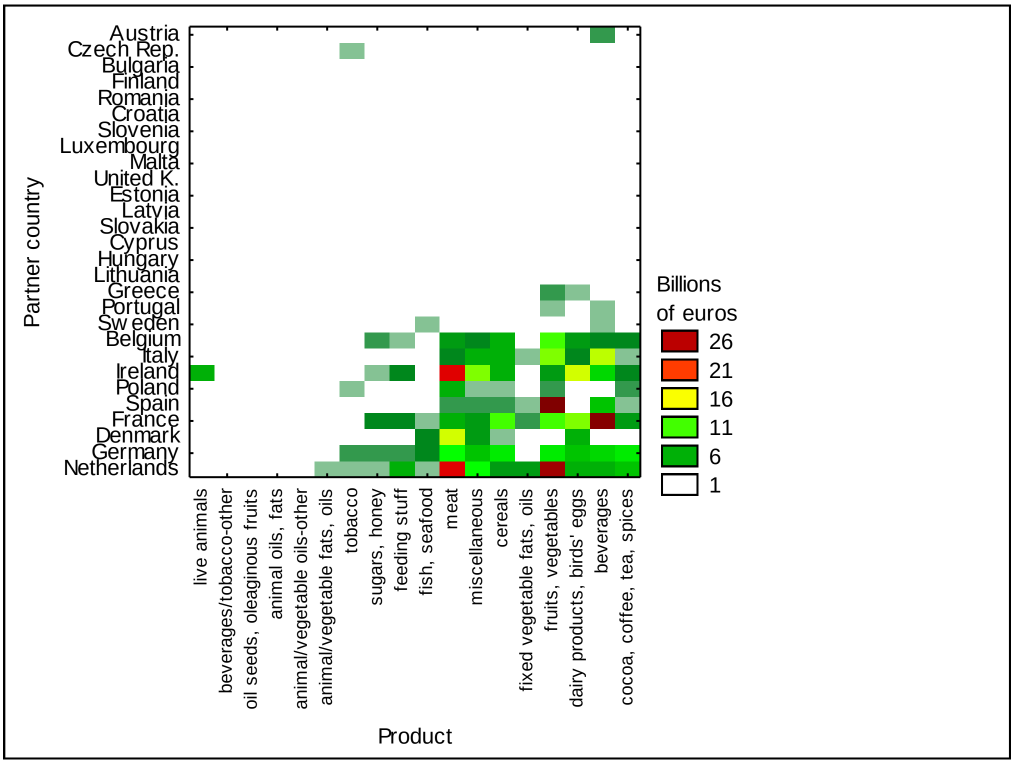

(b) product

Figure S32. Import of food to United Kingdom in 1999-2018 (billions of euros)

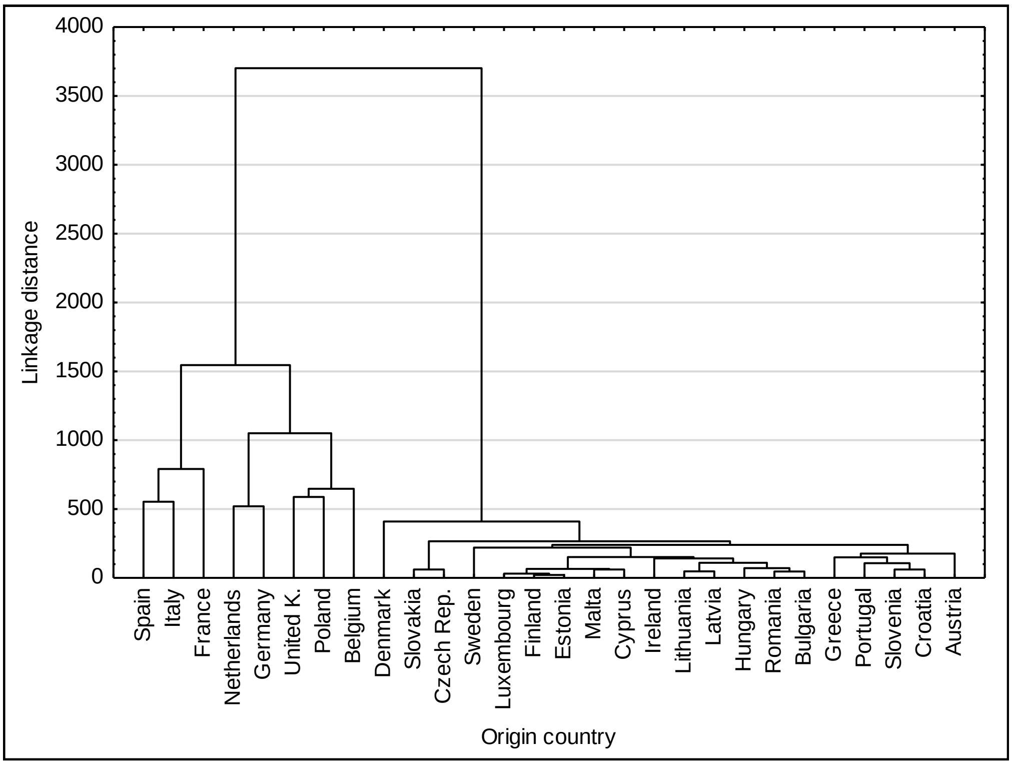

(a) joining

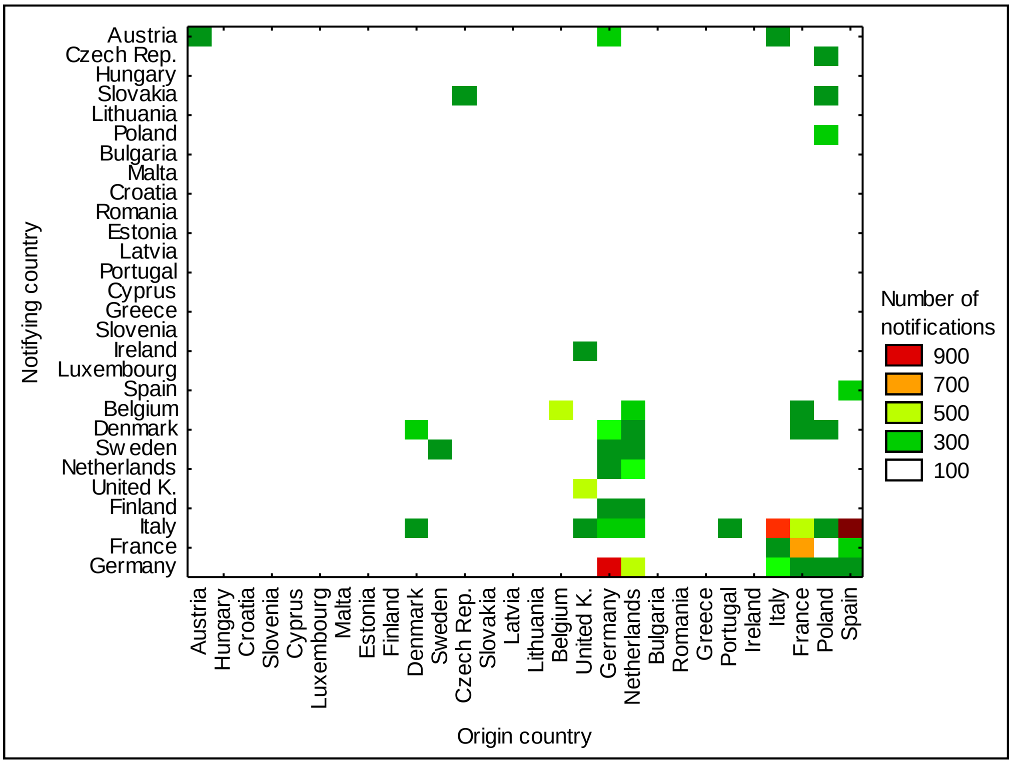

(b) two-way joining

Figure S33. Notifications on food in the RASFF in 1999-2018 within the European Union according to the notifying country

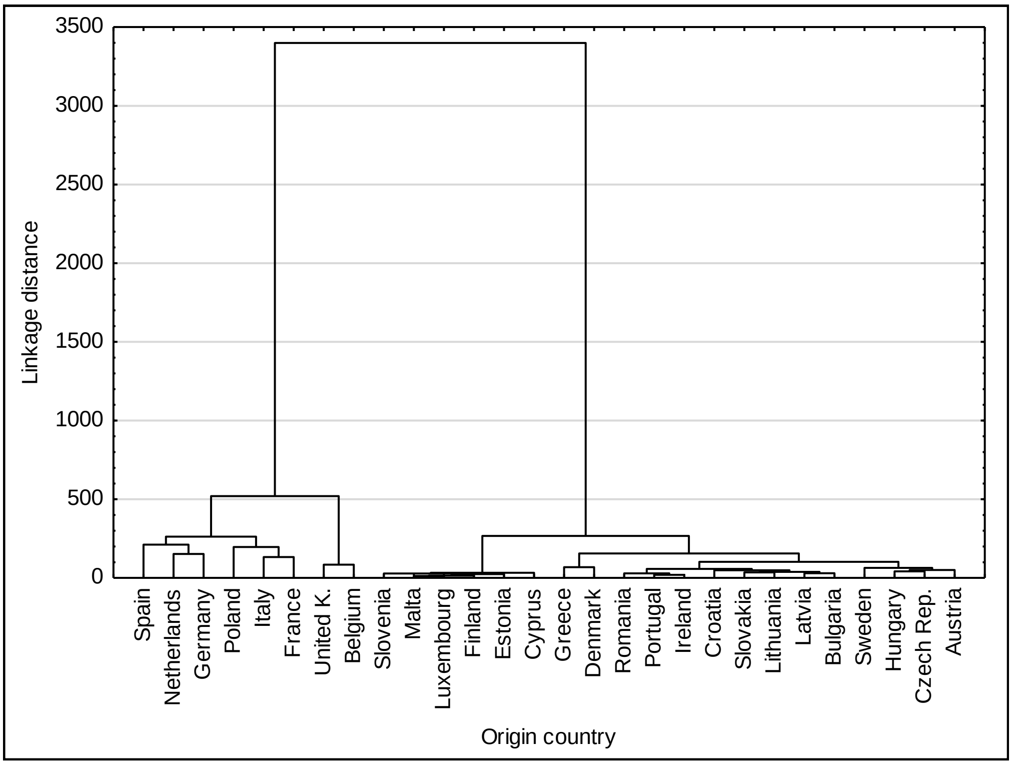

(a) joining

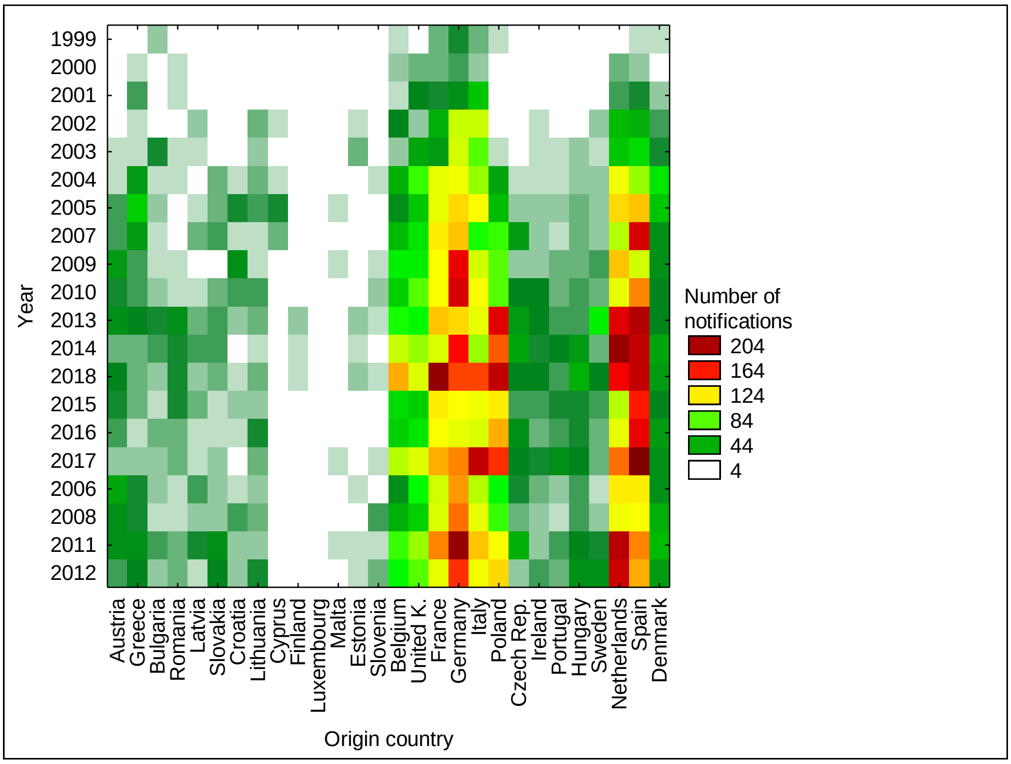

(b) two-way joining

Figure S34. Notifications on food in the RASFF in 1999-2018 within the European Union according to the year

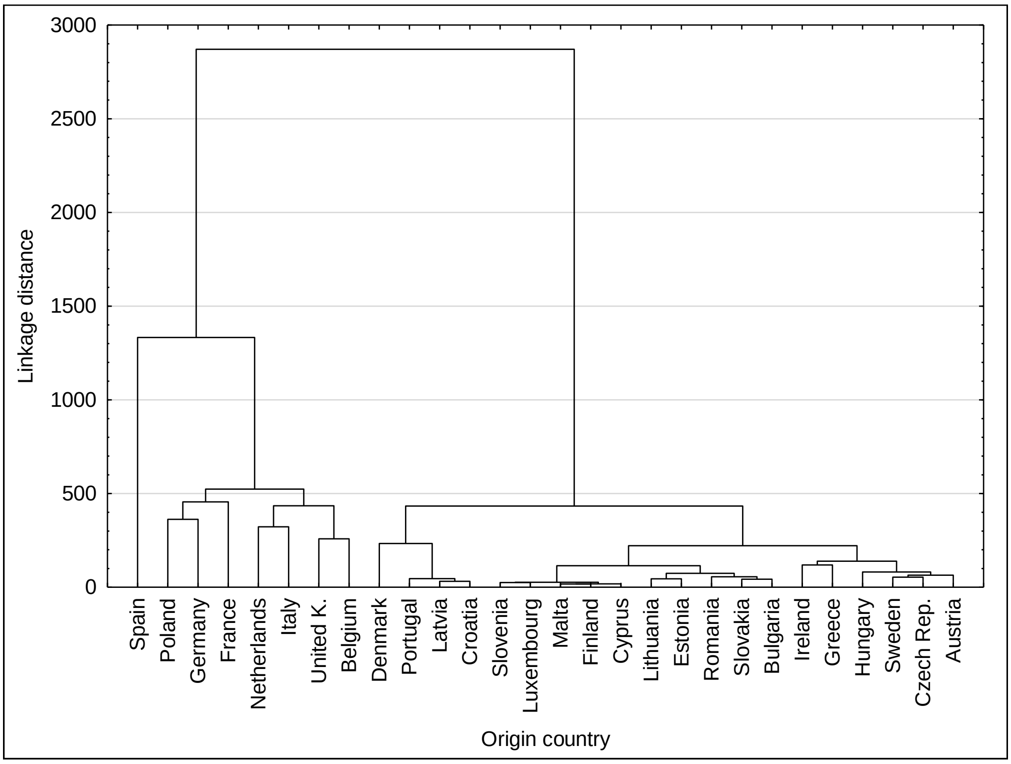

(a) joining

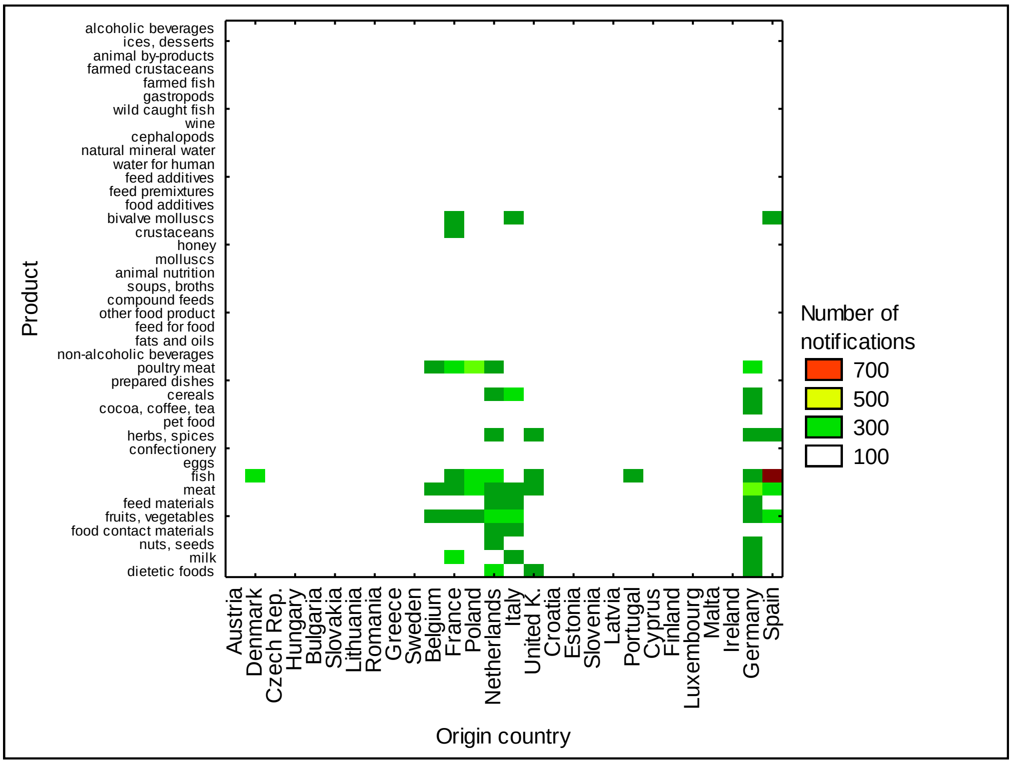

(b) two-way joining

Figure S35. Notifications on food in the RASFF in 1999-2018 within the European Union according to the product

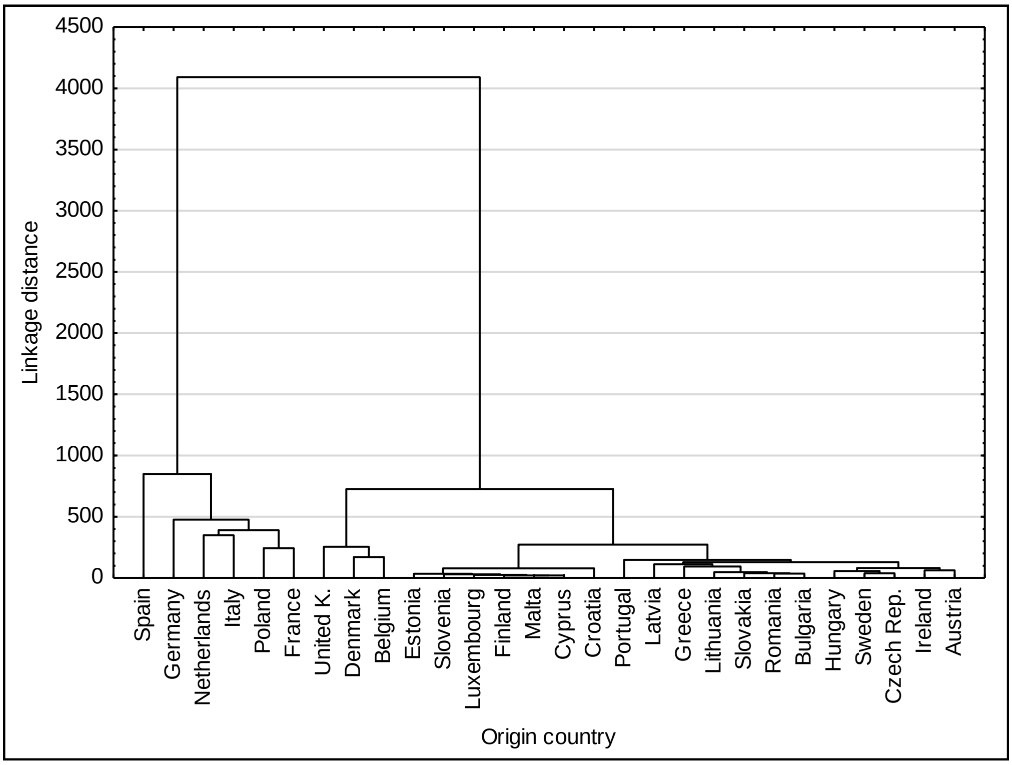

(a) joining

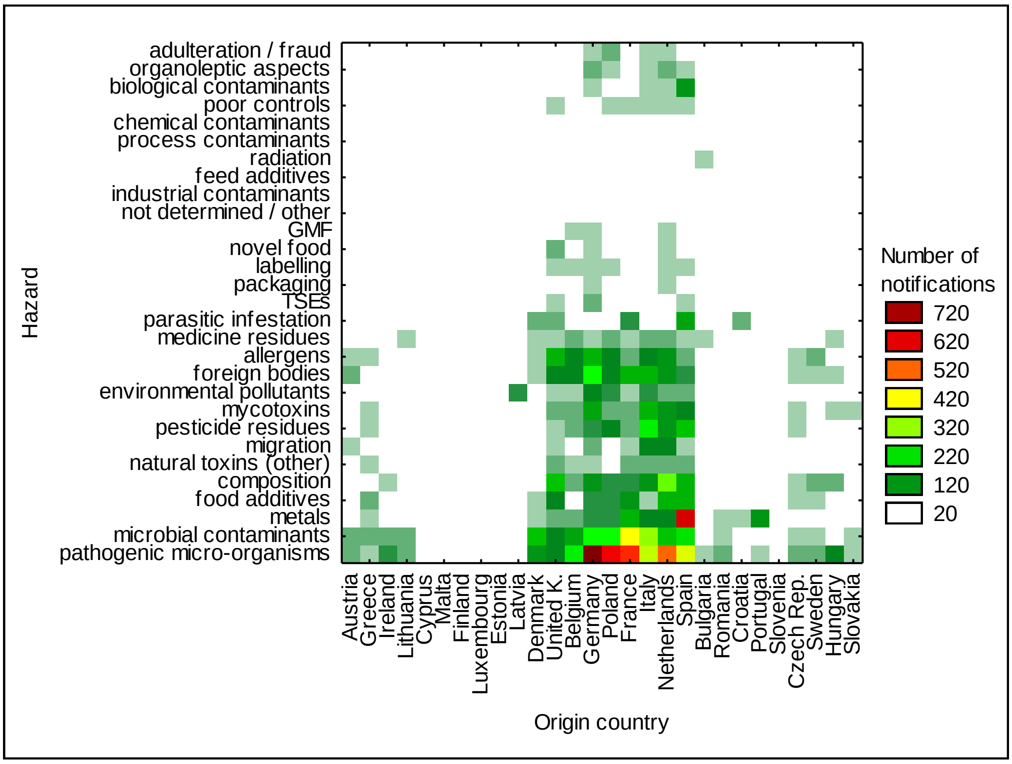

(b) two-way joining

Figure S36. Notifications on food in the RASFF in 1999-2018 within the European Union according to the hazard

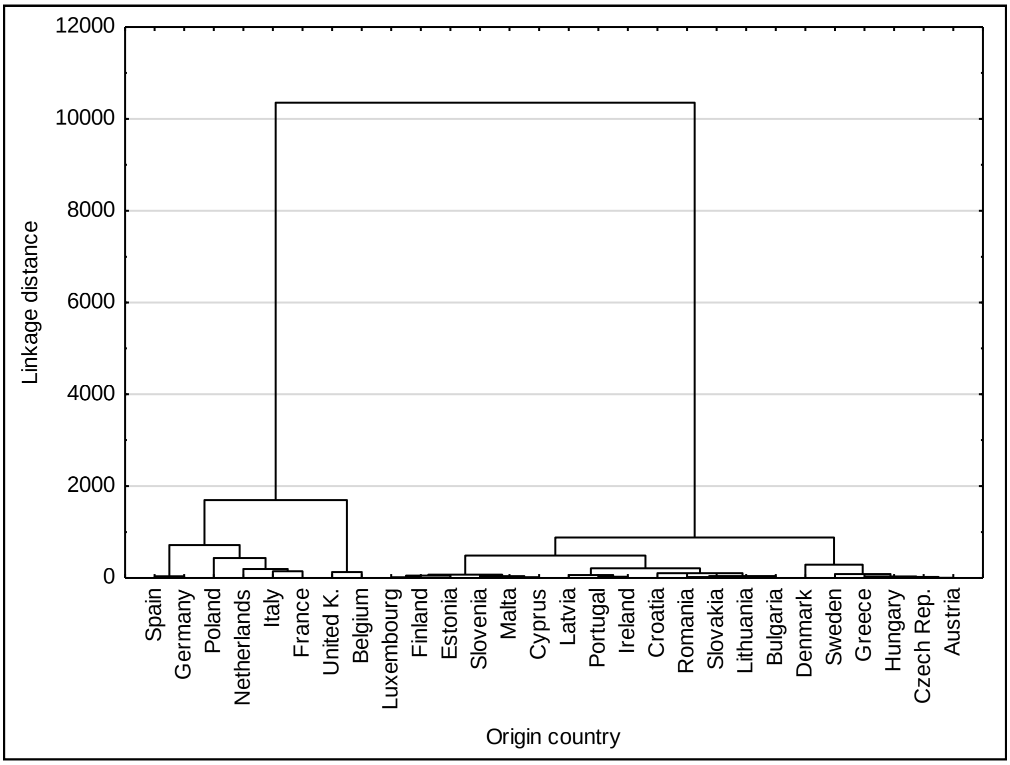

(a) joining

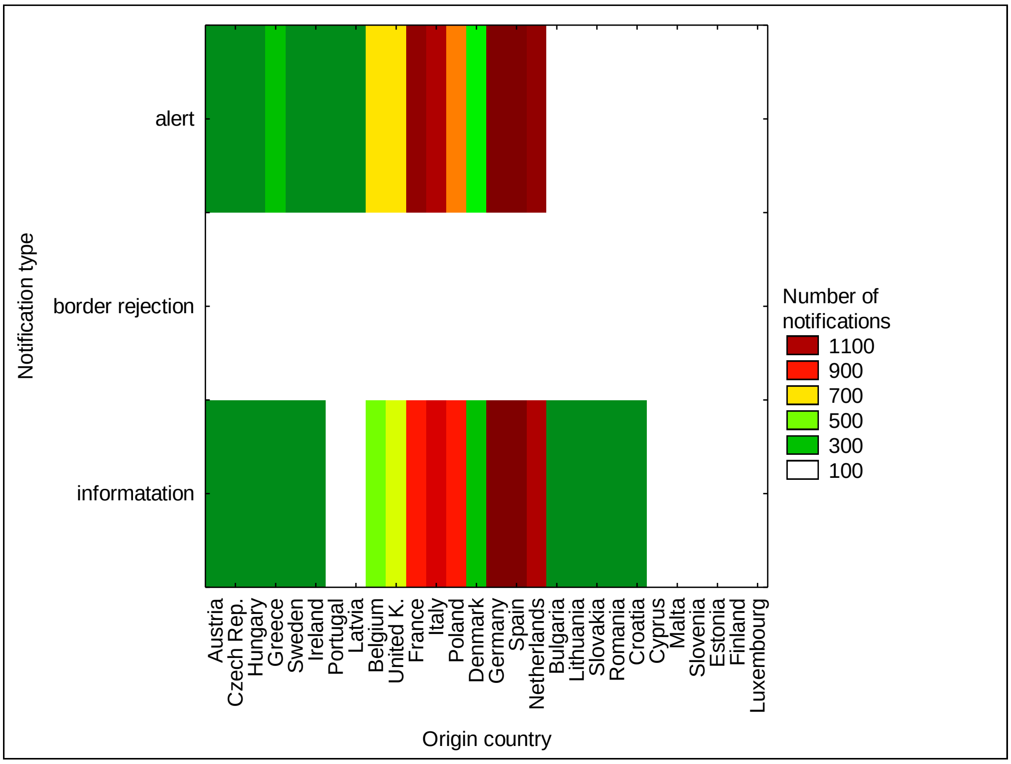

(b) two-way joining

Figure S37. Notifications on food in the RASFF in 1999-2018 within the European Union according to the notification type

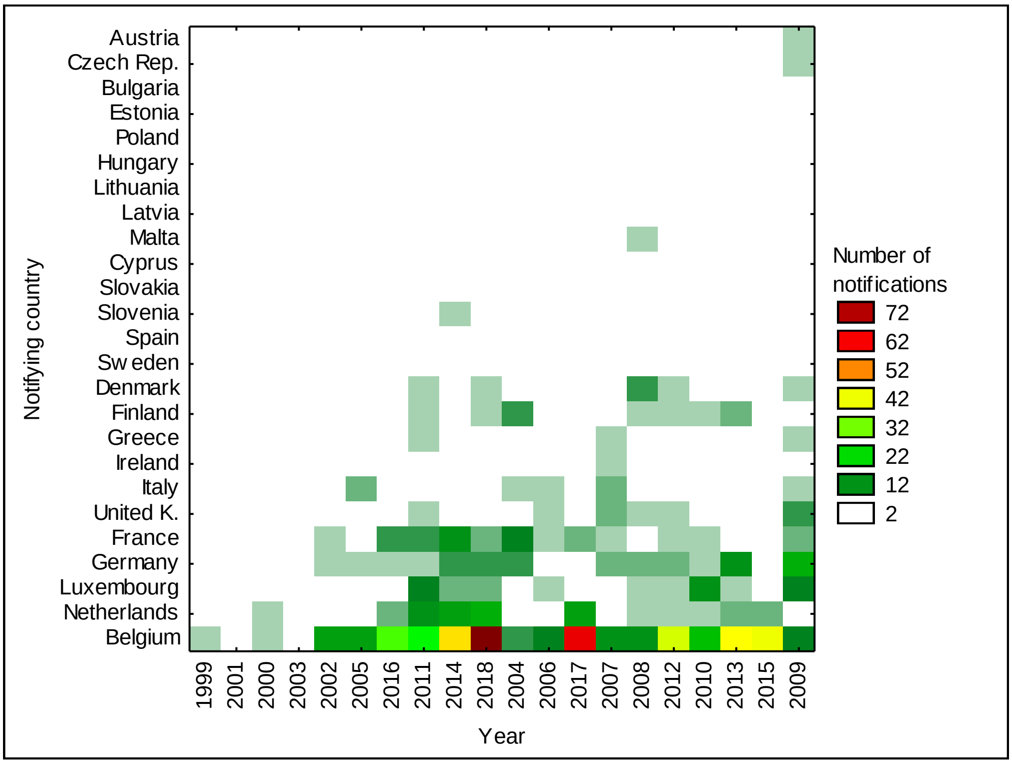

(a) year

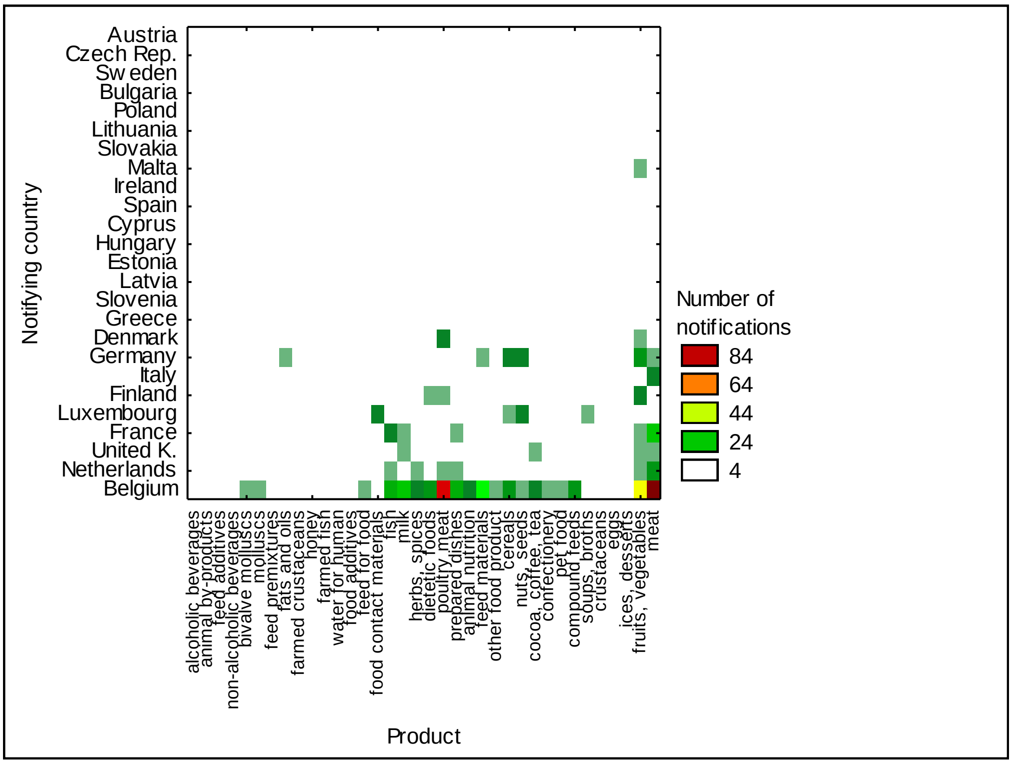

(b) product

Figure S38. Notifications on food in the RASFF in 1999-2018 originated from Belgium

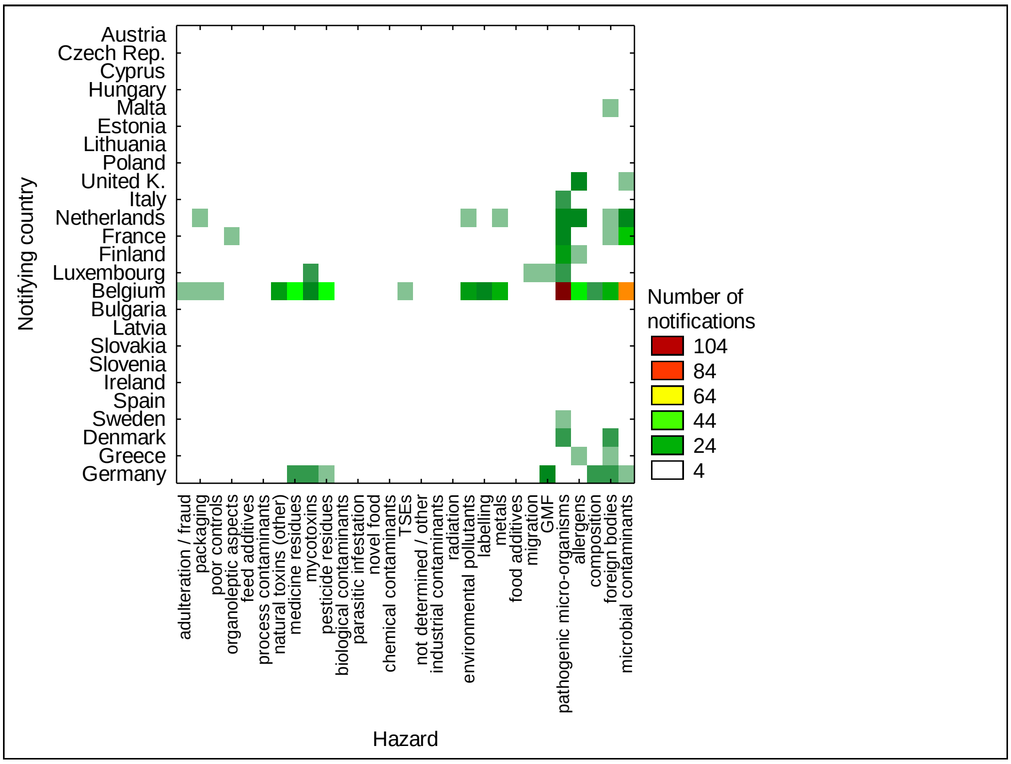

(c) hazard

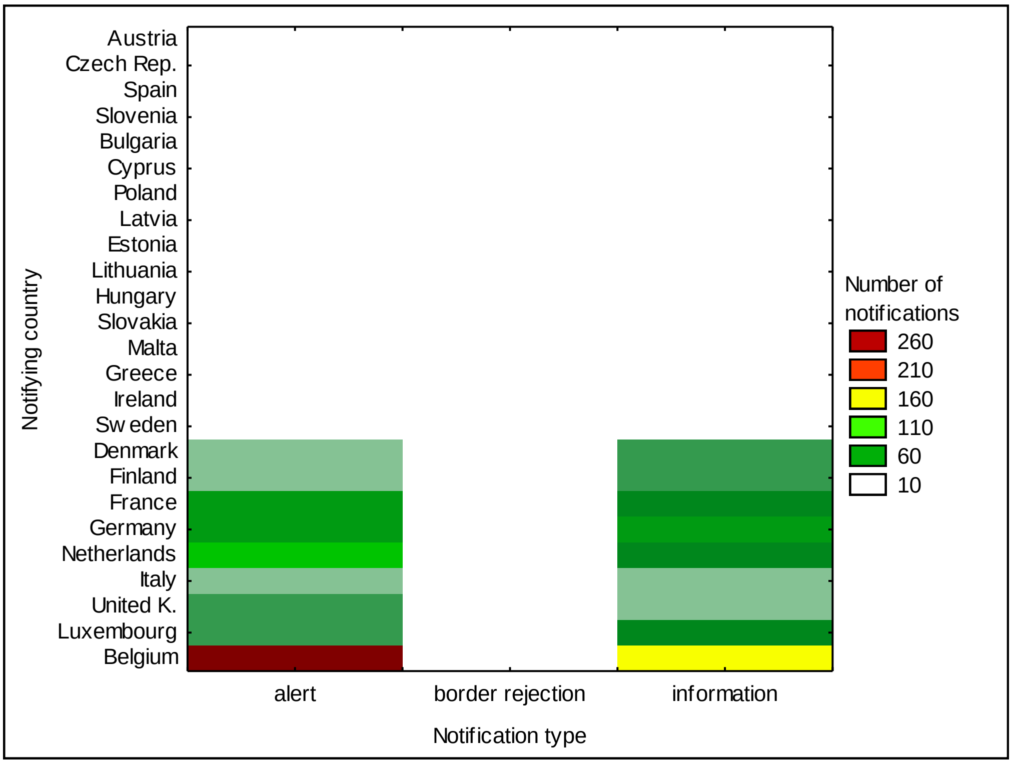

(d) notification type

Figure S38 continued. Notifications on food in the RASFF in 1999-2018 originated from Belgium

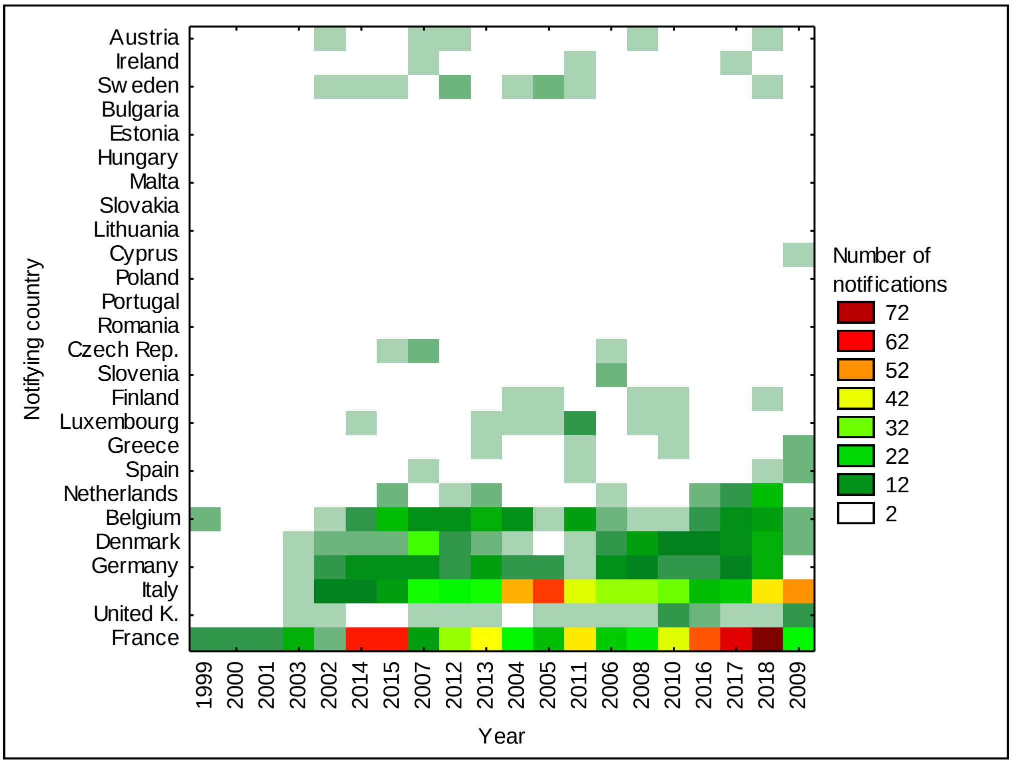

(a) year

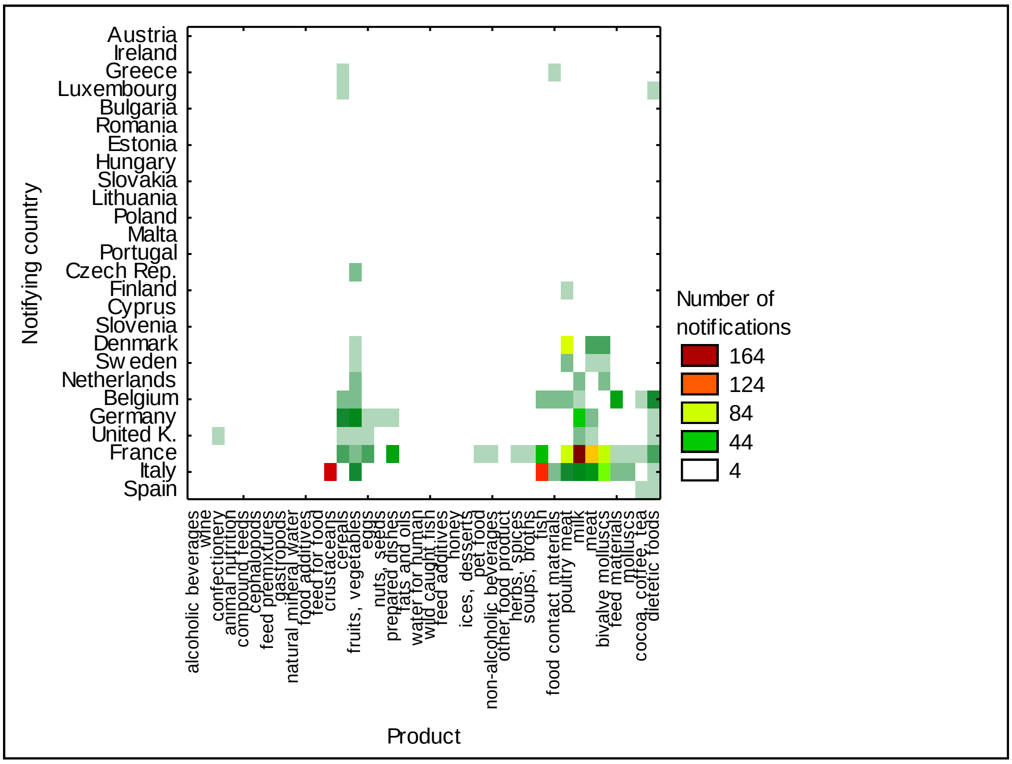

(b) product

Figure S39. Notifications on food in the RASFF in 1999-2018 originated from France

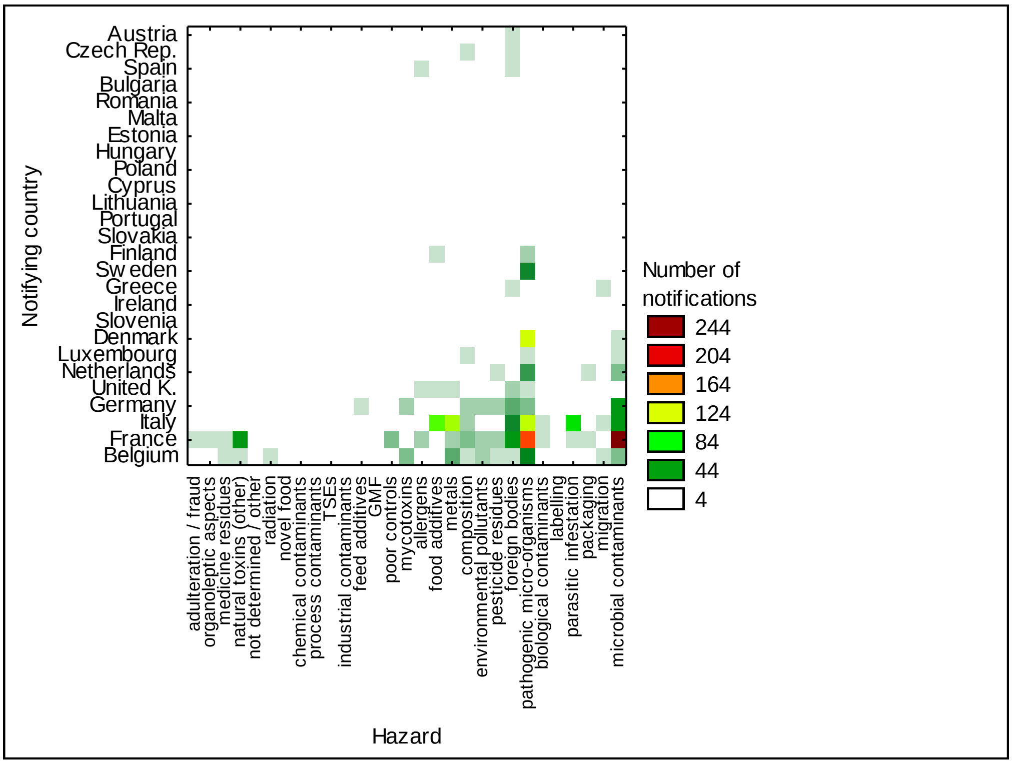

(c) hazard

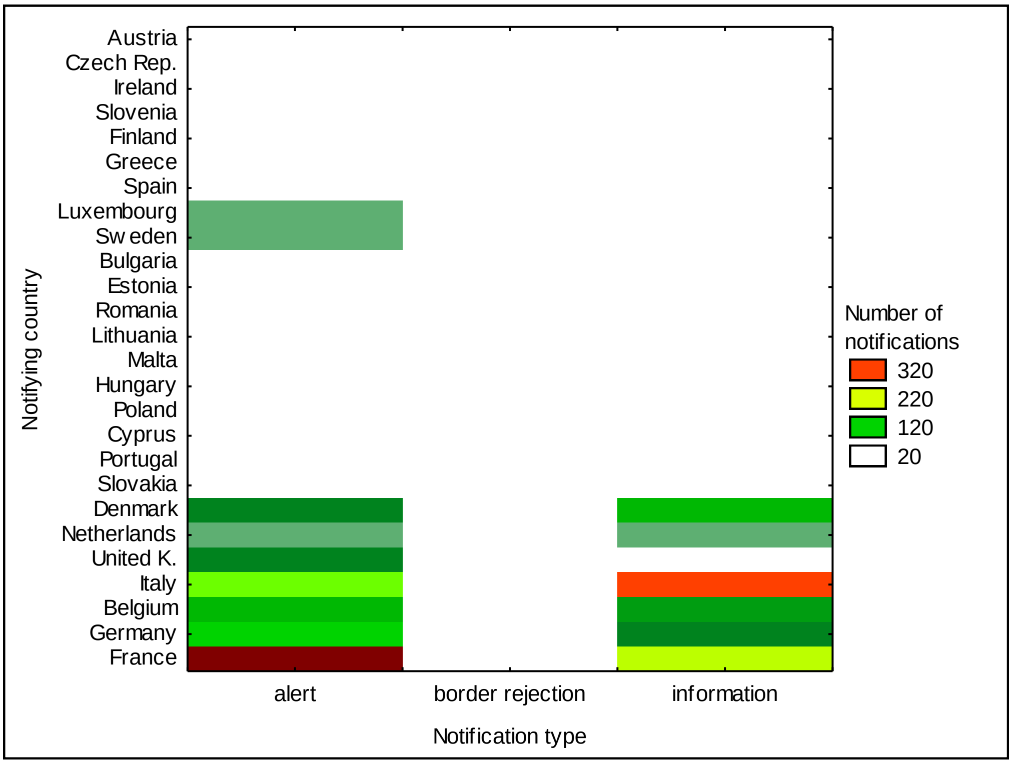

(d) notification type

Figure S39 continued. Notifications on food in the RASFF in 1999-2018 originated from France

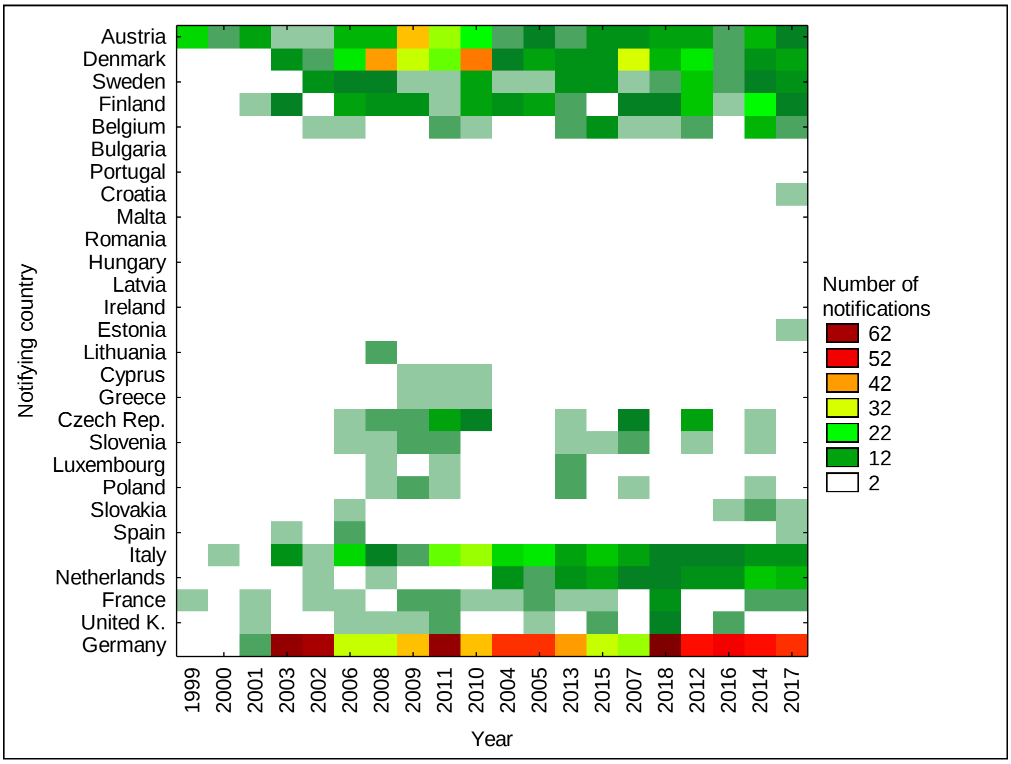

(a) year

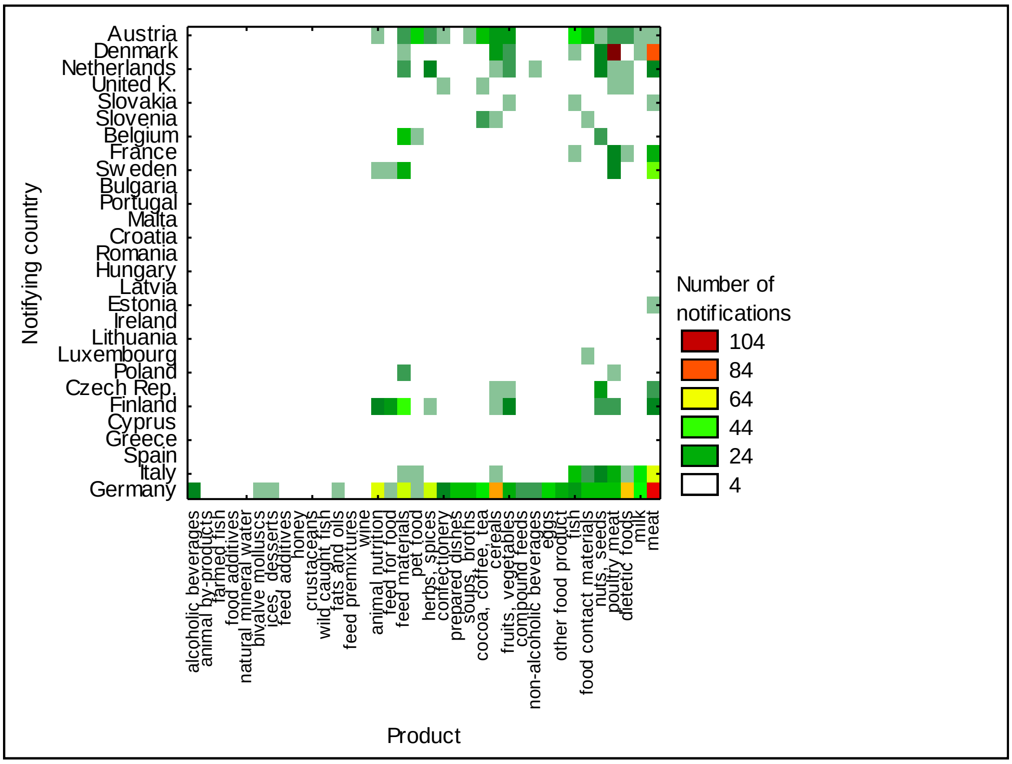

(b) product

Figure S40. Notifications on food in the RASFF in 1999-2018 originated from Germany

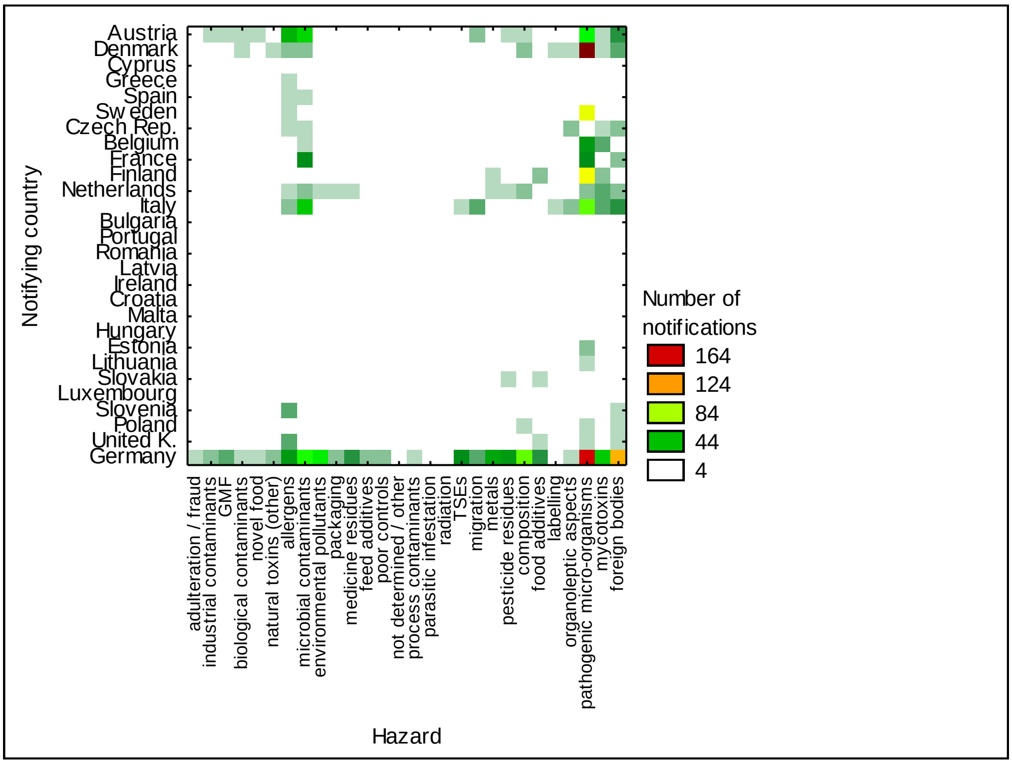

(c) hazard

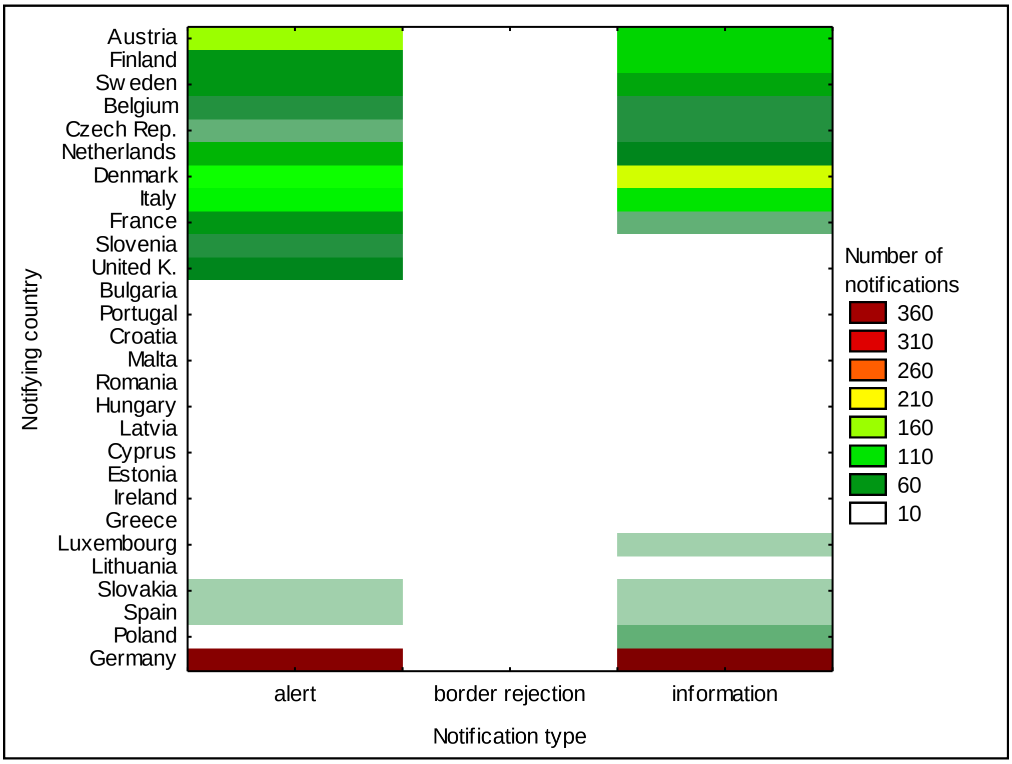

(d) notification type

Figure S40 continued. Notifications on food in the RASFF in 1999-2018 originated from Germany

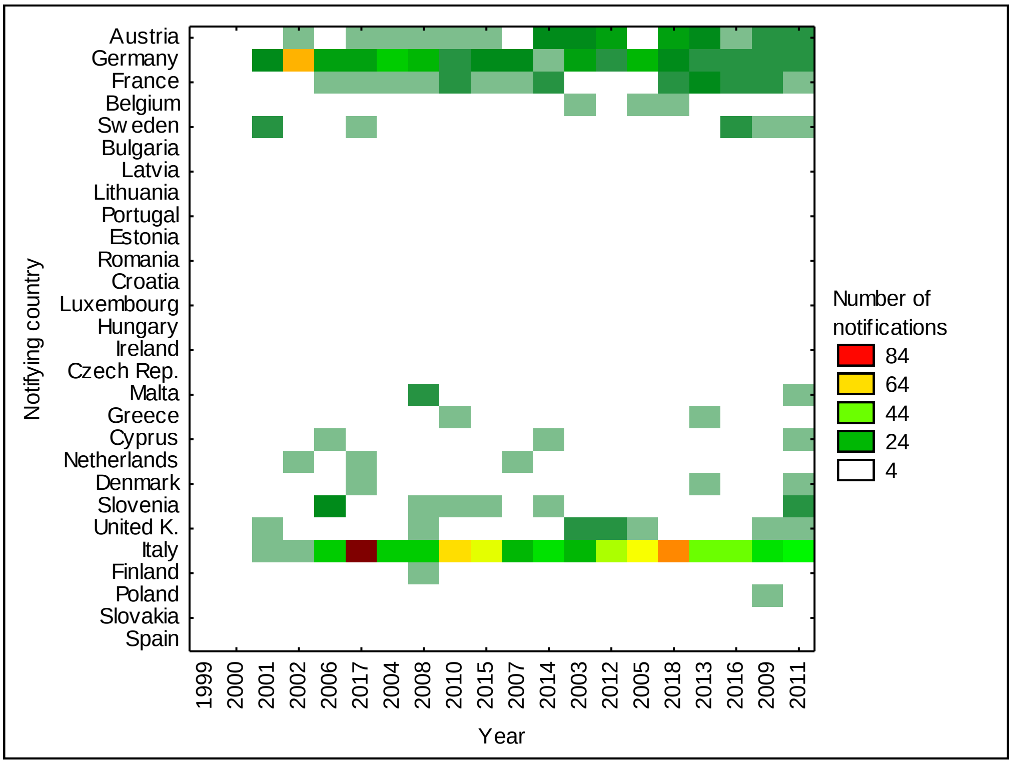

(a) year

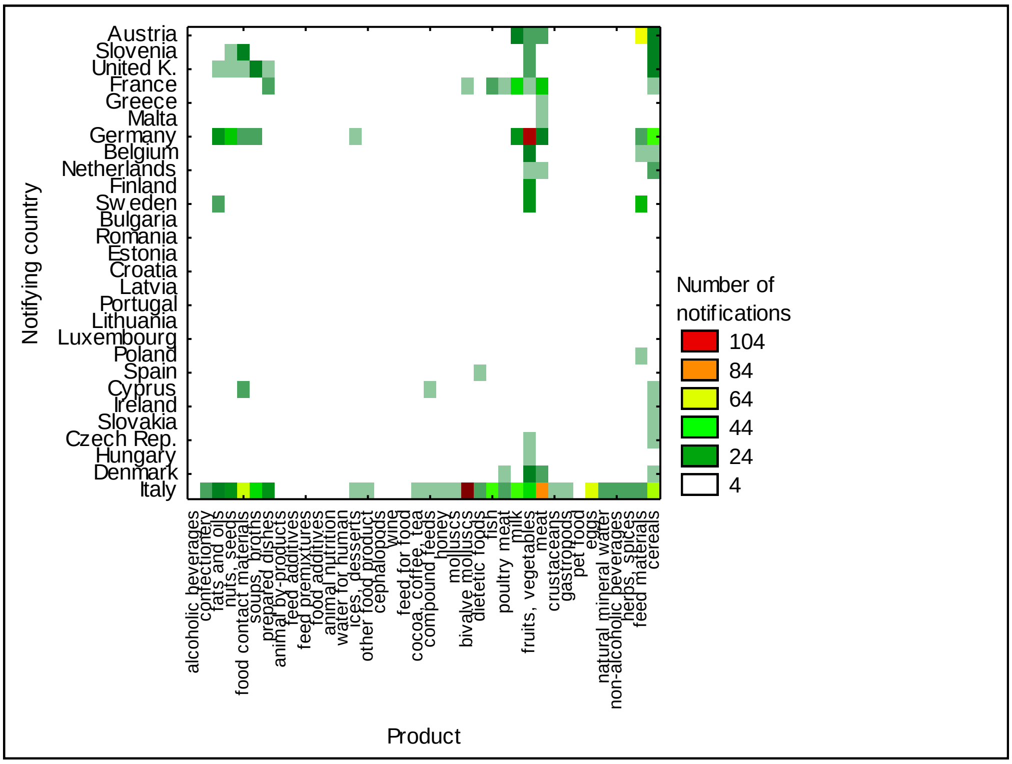

(b) product

Figure S41. Notifications on food in the RASFF in 1999-2018 originated from Italy

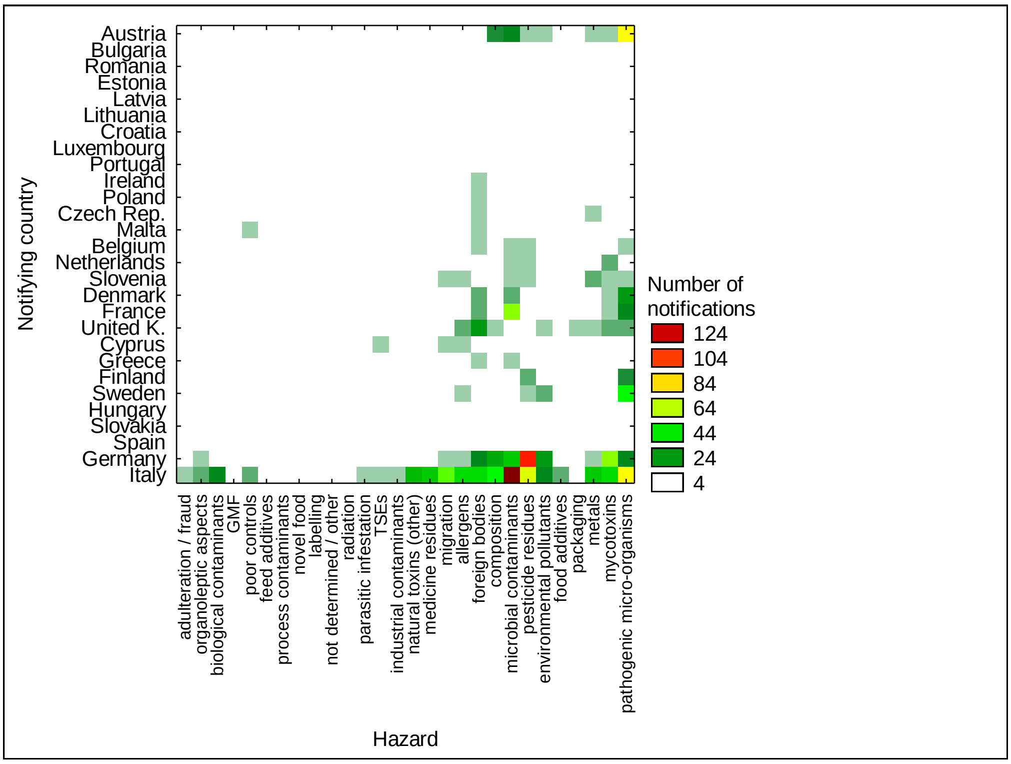

(c) hazard

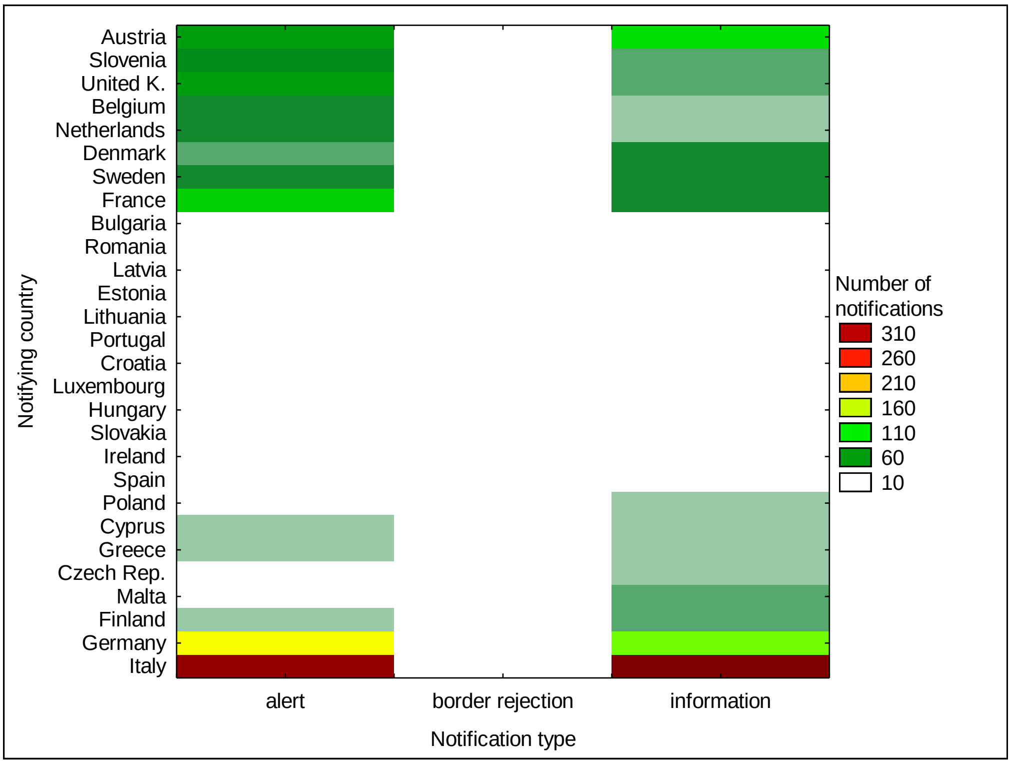

(d) notification type

Figure S41 continued. Notifications on food in the RASFF in 1999-2018 originated from Italy

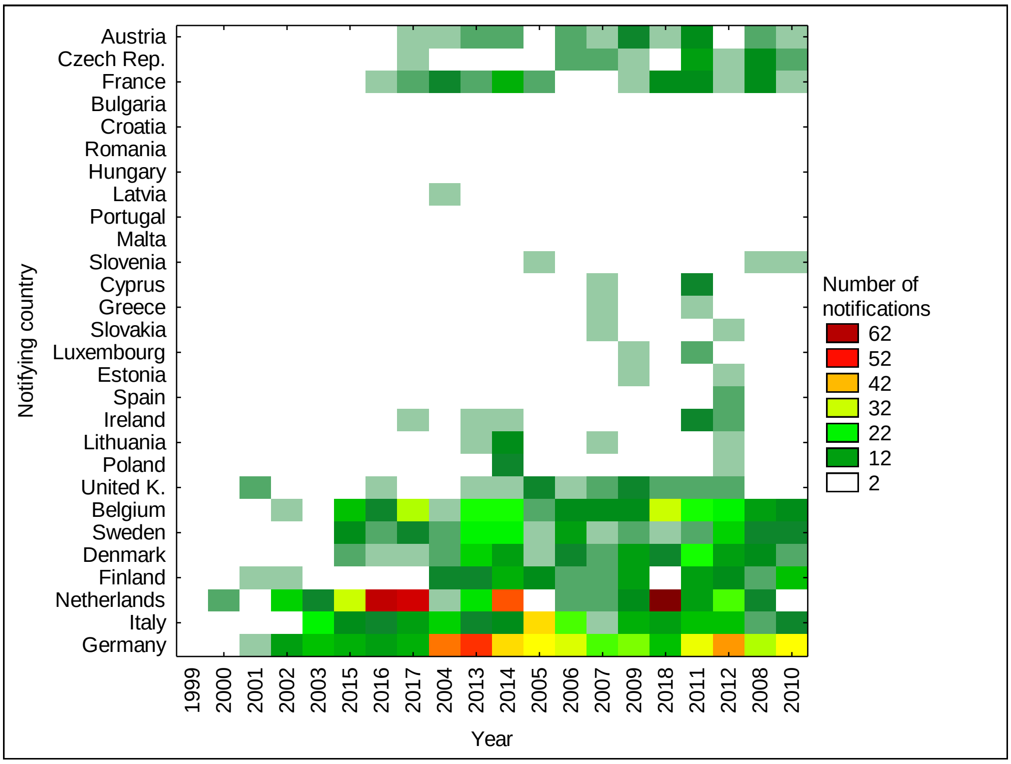

(a) year

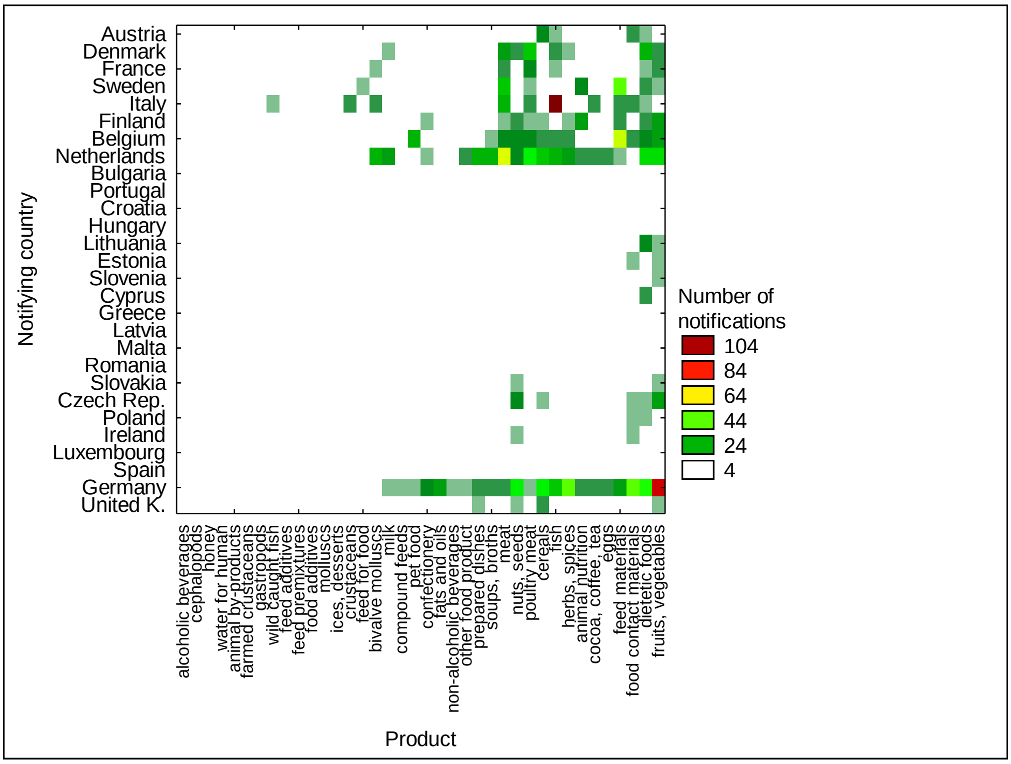

(b) product

Figure S42. Notifications on food in the RASFF in 1999-2018 originated from the Netherlands

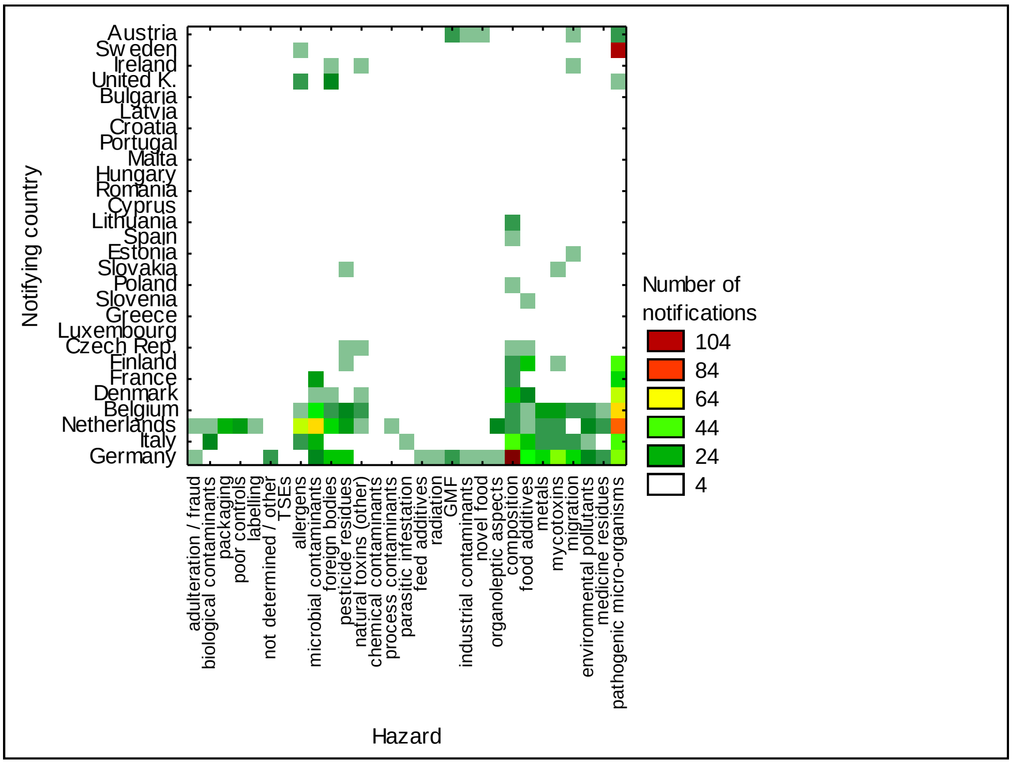

(c) hazard

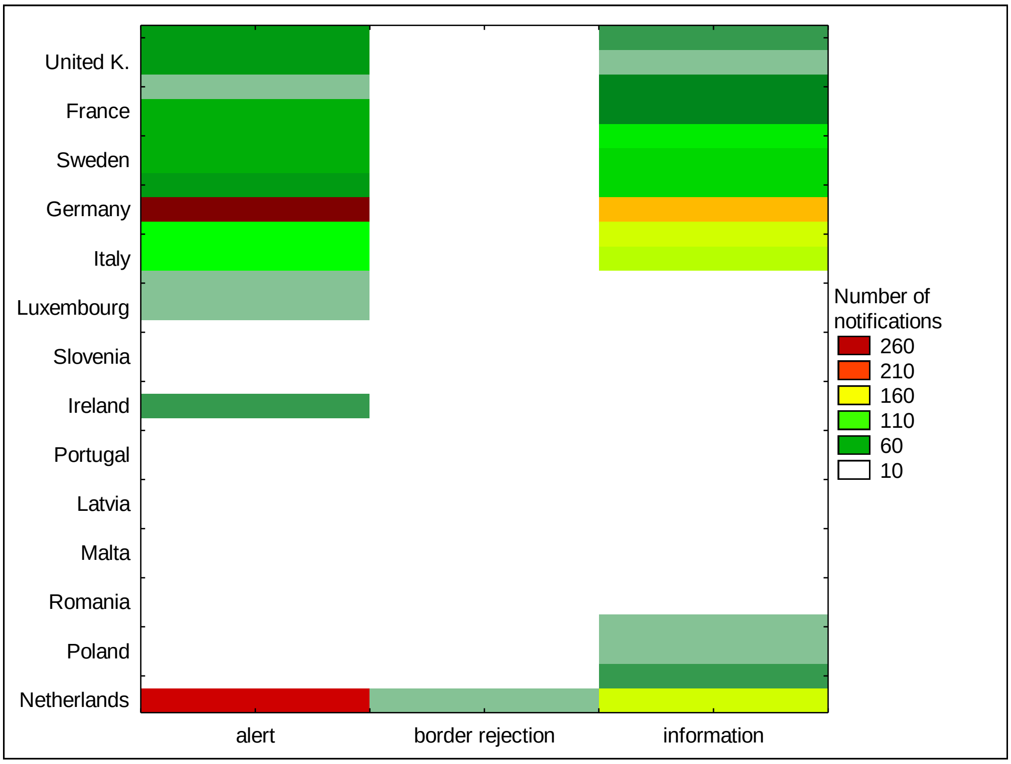

(d) notification type

Figure S42 continued. Notifications on food in the RASFF in 1999-2018 originated from the Netherlands

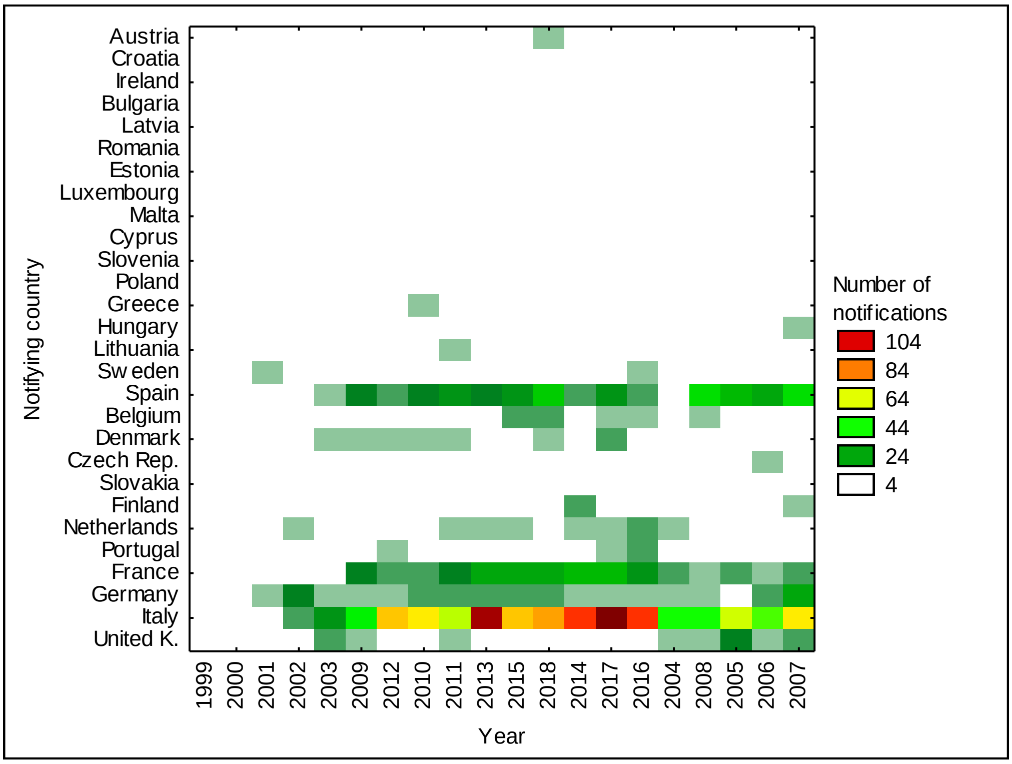

(a) year

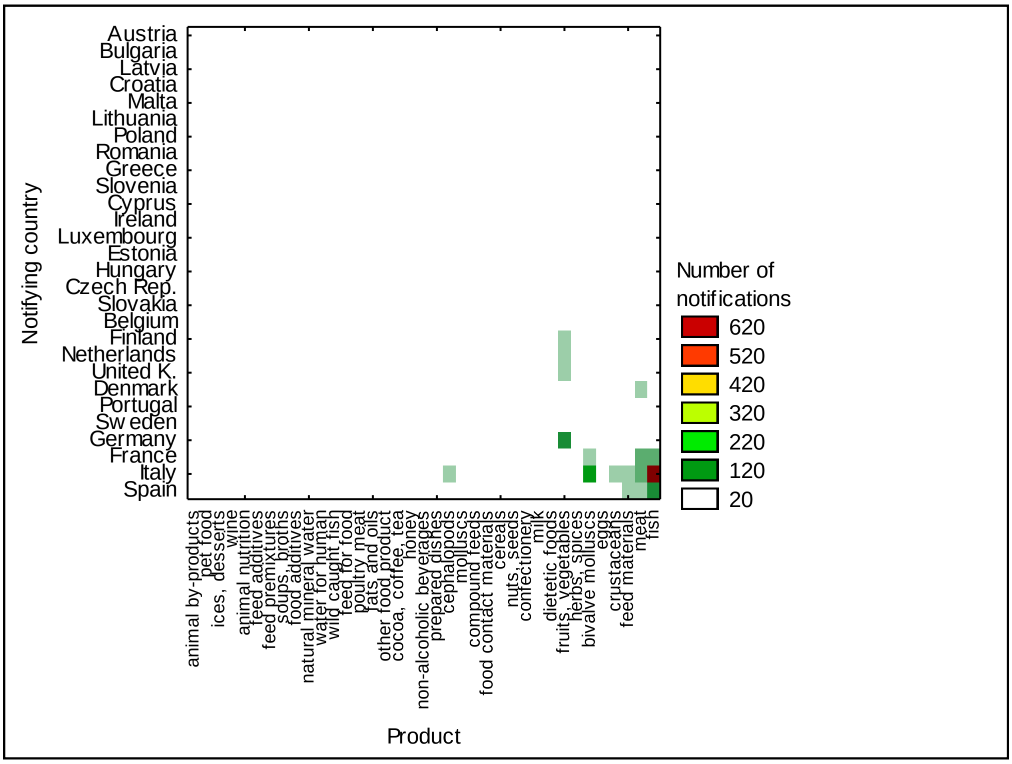

(b) product

Figure S43. Notifications on food in the RASFF in 1999-2018 originated from Spain

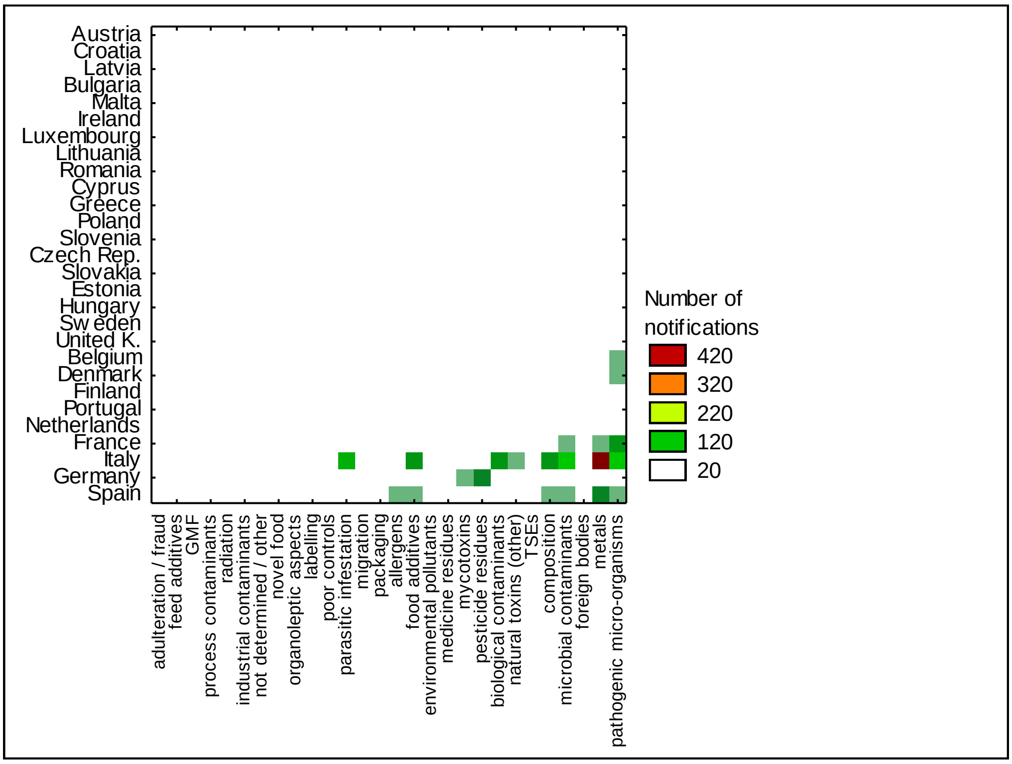

(c) hazard

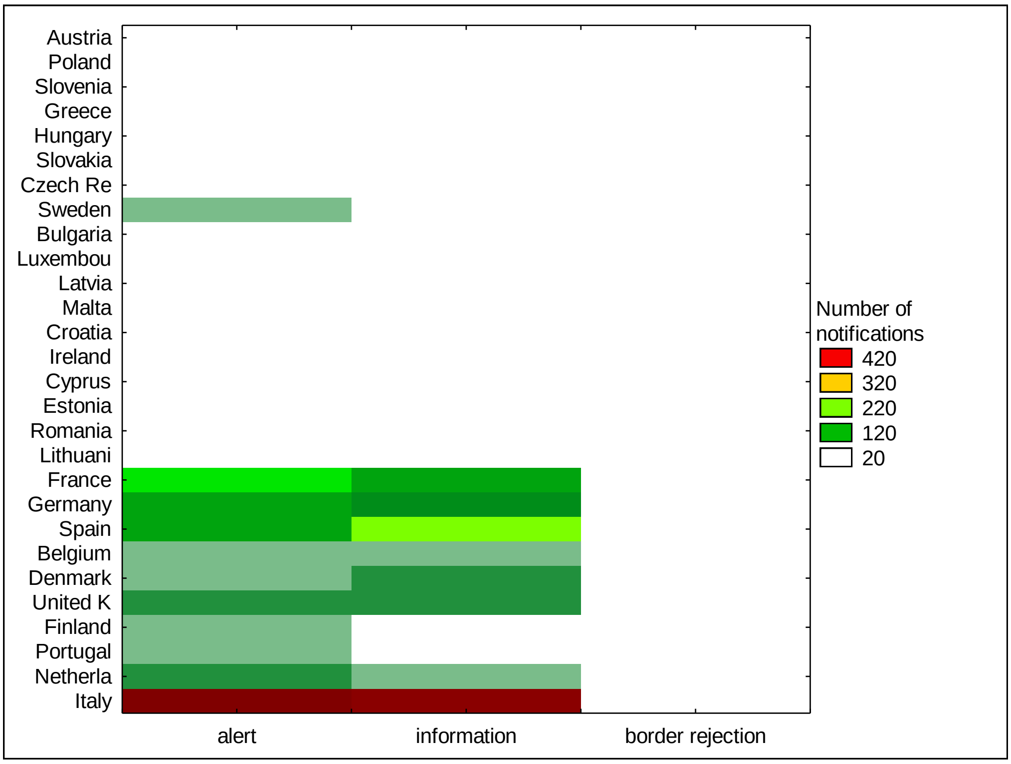

(d) notification type

Figure S43 continued. Notifications on food in the RASFF in 1999-2018 originated from Spain

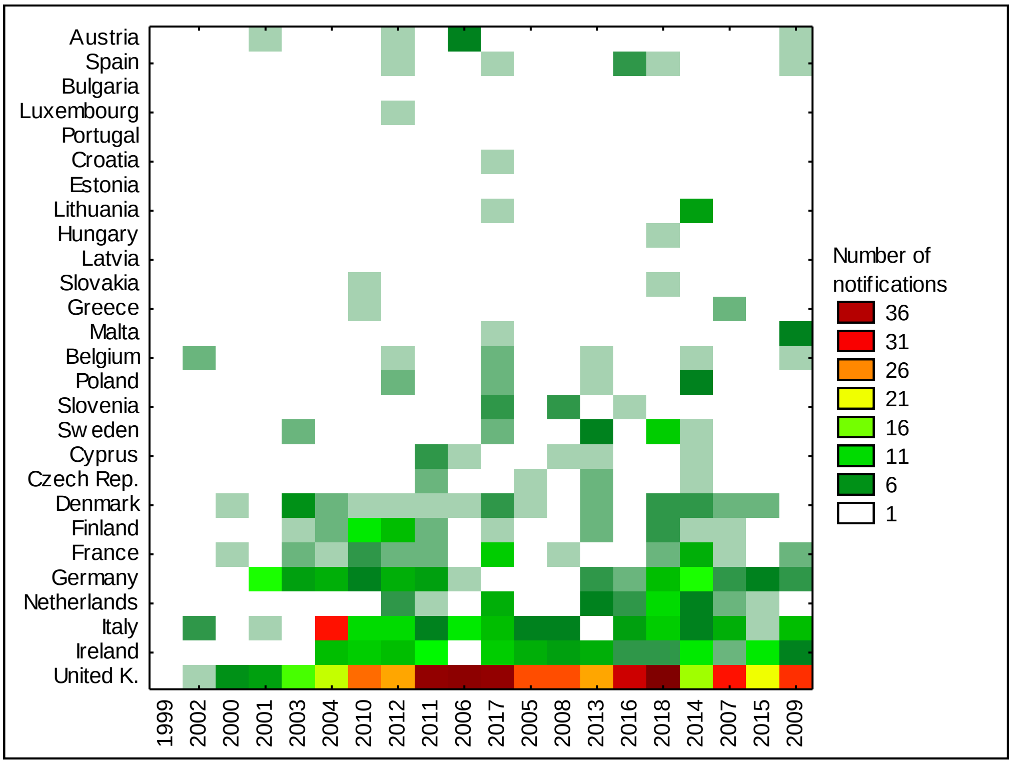

(a) year

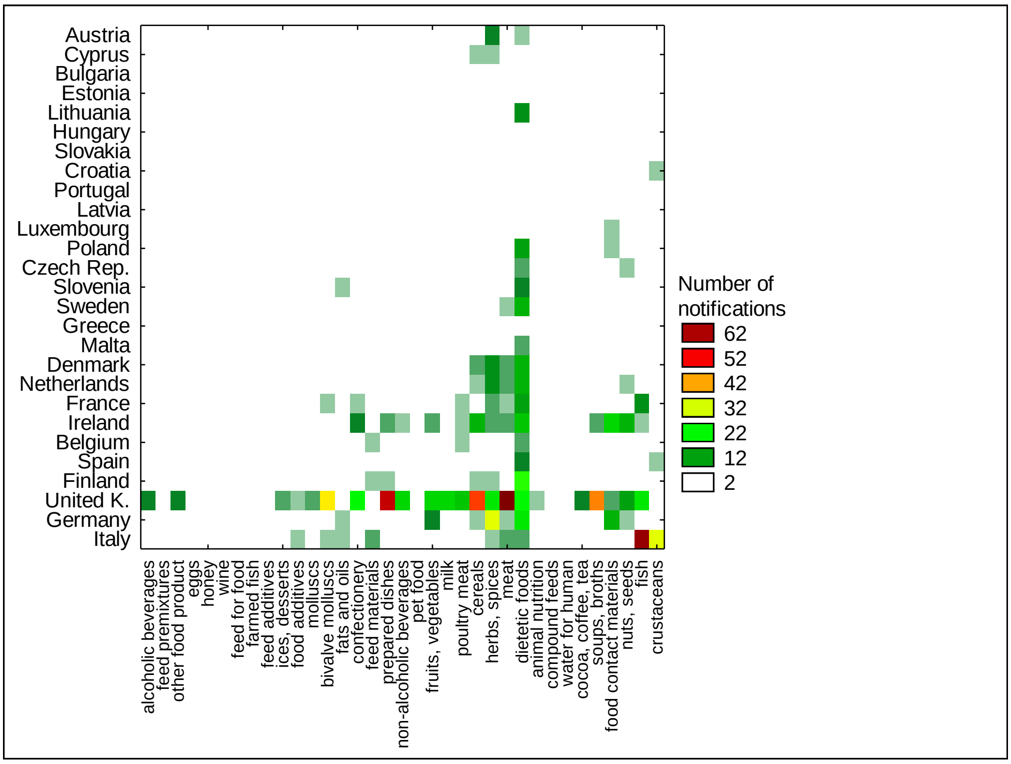

(b) product

Figure S44. Notifications on food in the RASFF in 1999-2018 originated from the United Kingdom

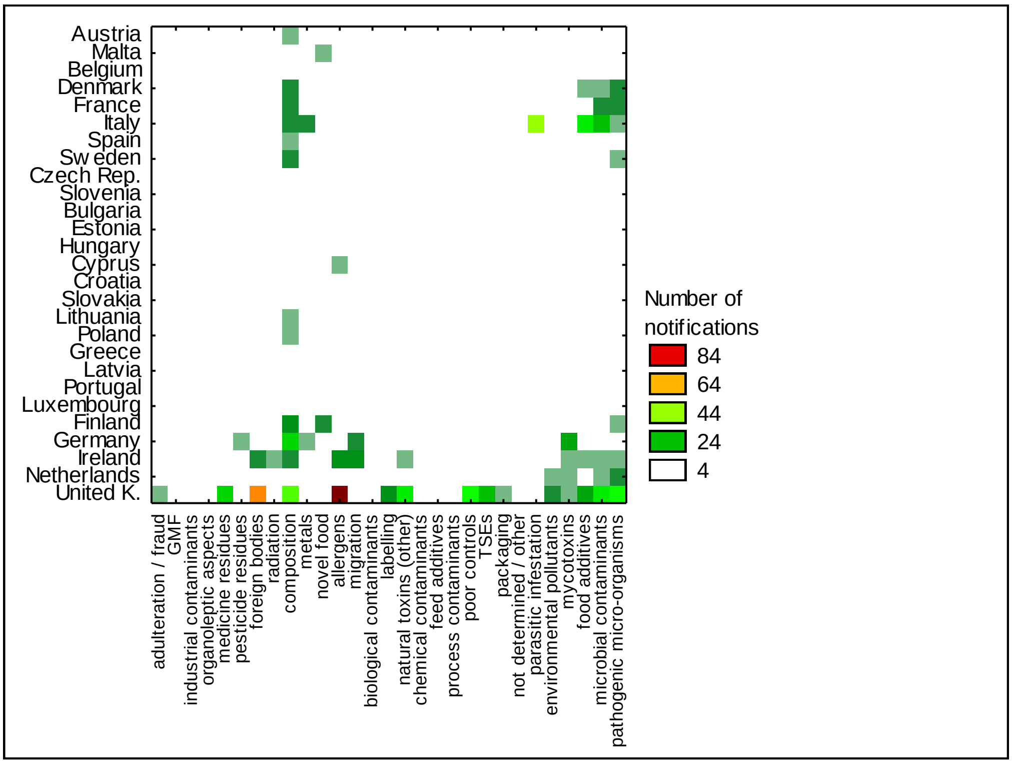

(c) hazard

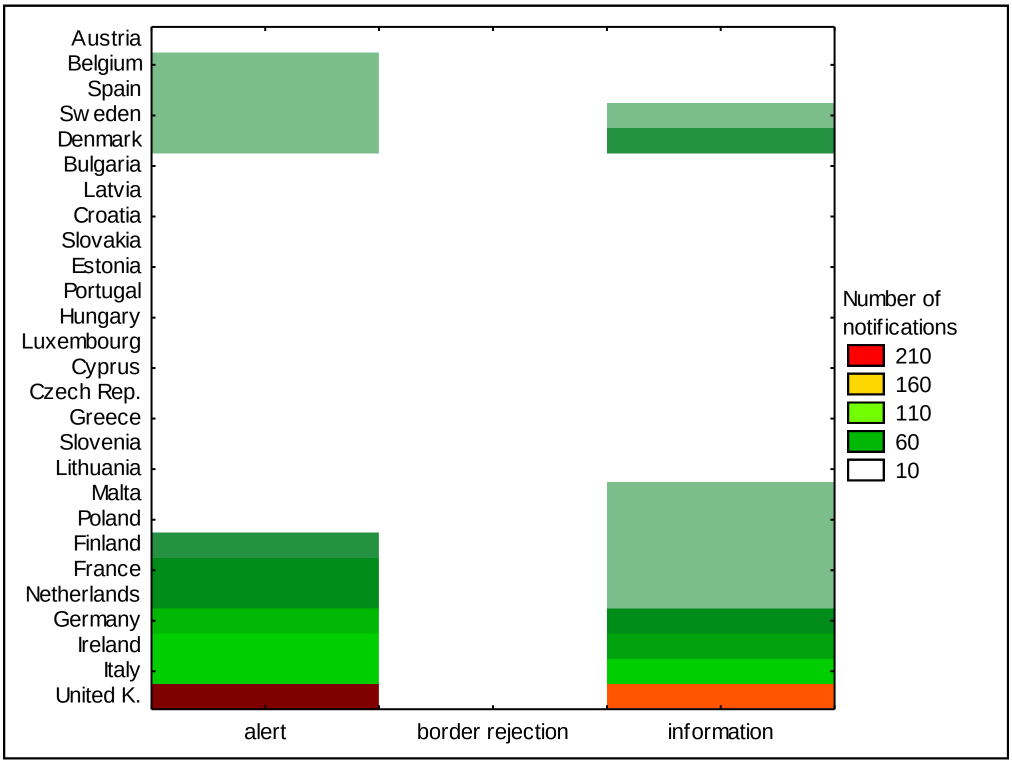

(d) notification type

Figure S44 continued. Notifications on food in the RASFF in 1999-2018 originated from the United Kingdom
